# Dancing through Life: Molecular Dynamics Simulations and Network-Centric Modeling of Allosteric Mechanisms in Hsp70 and Hsp110 Chaperone Proteins

**DOI:** 10.1371/journal.pone.0143752

**Published:** 2015-11-30

**Authors:** Gabrielle Stetz, Gennady M. Verkhivker

**Affiliations:** 1 Graduate Program in Computational and Data Sciences, Schmid College of Science and Technology, Chapman University, Orange, California, United States of America; 2 Chapman University School of Pharmacy, Irvine, California, United States of America; University of Akron, UNITED STATES

## Abstract

Hsp70 and Hsp110 chaperones play an important role in regulating cellular processes that involve protein folding and stabilization, which are essential for the integrity of signaling networks. Although many aspects of allosteric regulatory mechanisms in Hsp70 and Hsp110 chaperones have been extensively studied and significantly advanced in recent experimental studies, the atomistic picture of signal propagation and energetics of dynamics-based communication still remain unresolved. In this work, we have combined molecular dynamics simulations and protein stability analysis of the chaperone structures with the network modeling of residue interaction networks to characterize molecular determinants of allosteric mechanisms. We have shown that allosteric mechanisms of Hsp70 and Hsp110 chaperones may be primarily determined by nucleotide-induced redistribution of local conformational ensembles in the inter-domain regions and the substrate binding domain. Conformational dynamics and energetics of the peptide substrate binding with the Hsp70 structures has been analyzed using free energy calculations, revealing allosteric hotspots that control negative cooperativity between regulatory sites. The results have indicated that cooperative interactions may promote a population-shift mechanism in Hsp70, in which functional residues are organized in a broad and robust allosteric network that can link the nucleotide-binding site and the substrate-binding regions. A smaller allosteric network in Hsp110 structures may elicit an entropy-driven allostery that occurs in the absence of global structural changes. We have found that global mediating residues with high network centrality may be organized in stable local communities that are indispensable for structural stability and efficient allosteric communications. The network-centric analysis of allosteric interactions has also established that centrality of functional residues could correlate with their sensitivity to mutations across diverse chaperone functions. This study reconciles a wide spectrum of structural and functional experiments by demonstrating how integration of molecular simulations and network-centric modeling may explain thermodynamic and mechanistic aspects of allosteric regulation in chaperones.

## Introduction

**Fig 1:**
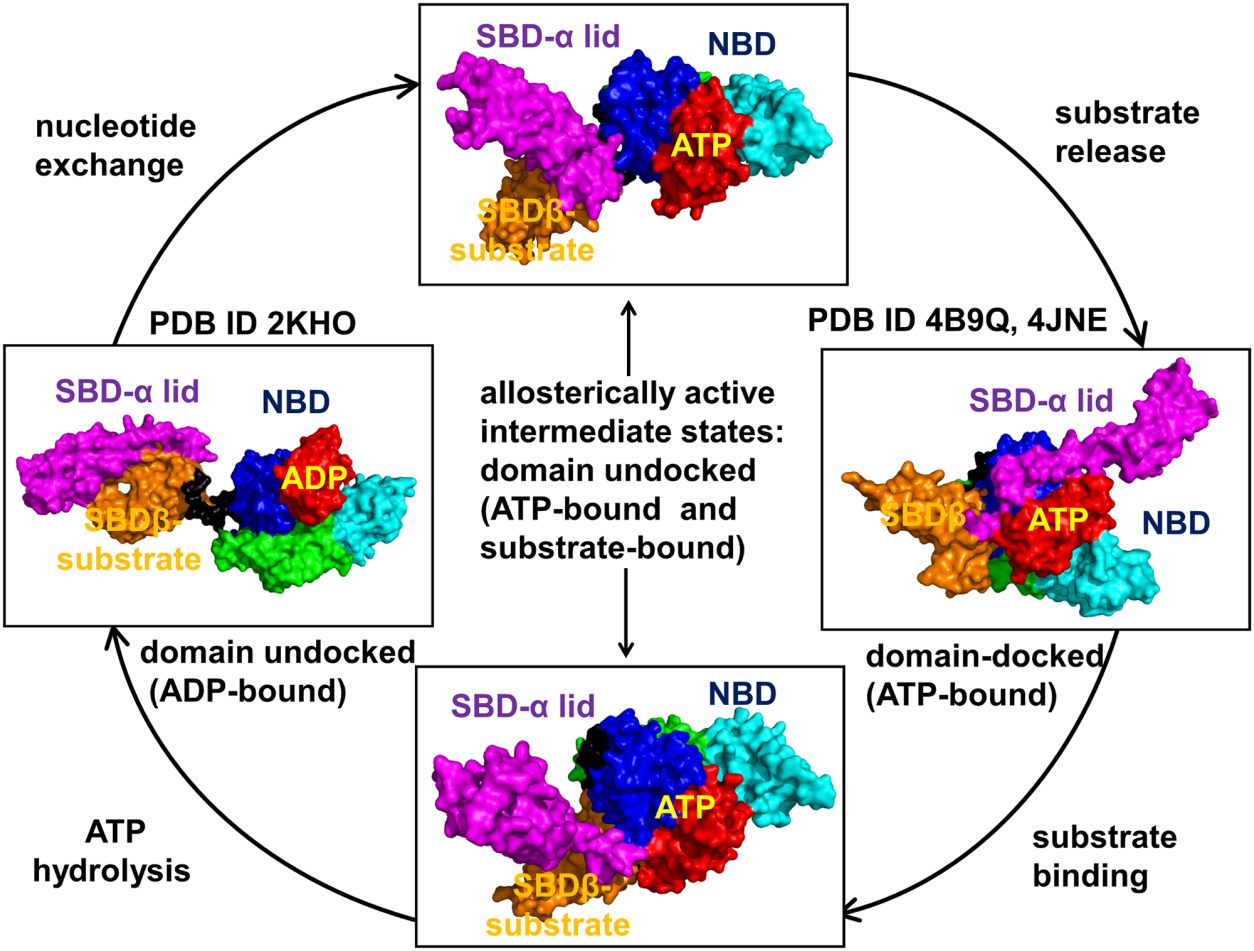
The Functional Cycle of Hsp70 Chaperones. The functional cycle of Hsp70 chaperones. The main steps of the allosteric cycle include the nucleotide exchange in the closed, ADP-bound form (pdb id 2KHO); the formation of partially undocked ATP/substrate-bound intermediate; substrate release and formation of the domain-docked ATP-bound form (pdb id 4B9Q, 4JNE); substrate binding and partial domain undocking in the ATP/substrate-bound state; ATP hydrolysis and stabilization of the domain-undocked, ADP-bound form. The structures are shown in a surface representation and the main structural elements are annotated. The NBD subdomains are colored as follows: IA (in blue), IB (in red), IIA (in green), IIB (in cyan), the inter-domain linker (in black), SBD-α (in magenta), and SBD-β (in orange). The inter-domain interfaces NBD/SBD-β, NBD/SBD-α and SBD-β/SBD-α form allosteric hotspots of the Hsp70 functional cycle that are modulated through binding of nucleotides and substrates. The Pymol program was used for rendering protein structures (The PyMOL Molecular Graphics System, Version 1.2r3pre, Schrödinger, and LLC).

The 70-kilodalton (kDa) Heat shock protein (Hsp70) belongs to a ubiquitous and abundant family of molecular chaperones that play an important role in various cellular processes that involve protein folding, protein quality control and stabilization, trafficking, and turnover [1–7]. Hsp70 proteins occur in all domains of life and are among most conserved proteins found in all organisms. Deregulations of signal transduction pathways are often linked to the Hsp70-regulated processes, and viability of Hsp70 as an attractive and validated drug target has been firmly established [8–12]. Each Hsp70 has two functional domains: a nucleotide-binding domain (NBD), which binds and hydrolyzes ATP, and a substrate-binding domain (SBD), which binds extended polypeptides and client proteins [5–7]. Hsp70 functions are governed by the nucleotide-dependent allosteric cycle between an ATP-bound (open) and ADP-bound (closed) chaperone states, in which ATP binding in the NBD dramatically reduces the affinity for peptide substrates by accelerating binding and release rates in the SBD (Fig 1). ATP hydrolysis can restore Hsp70 to the high-affinity state, but is slow in the absence of substrates and co-chaperone proteins. This biochemical cycle involves a cascade of allosteric conformational changes (Fig 1), in which ATP binding and hydrolysis regulate binding thermodynamics and kinetics of substrate peptides with the SBD [5–7].

The Hsp70 cycle is also regulated by cooperative actions of co-chaperones that tune enzymatic activity of Hsp70 and guide its interactions with protein clients. The J-domain proteins bind to Hsp70 in a region between the NBD and SBD, thus stimulating ATP hydrolysis and client binding [13, 14]. Substrate binding to the SBD region and J-domain protein interactions with the NBD can synergistically stimulate the ATPase activity and increase ATP hydrolysis rate by more than 1,000-fold [13,14]. The nucleotide exchange factors (NEFs) bind the NBD of Hsp70 and catalyze the release of ADP and client proteins [15, 16]. Crystal structures of the isolated NBDs [17, 18] and SBDs [19–21] have provided an early insight into Hsp70 functions by revealing molecular details of the allosteric binding sites and substrate-protein interactions. Remarkably, the inter-domain coupling and chaperone activity can be sustained upon removal of the lid subdomain SBD-α, since such DnaK construct retained the ATP-induced substrate release and reciprocal stimulation of the ATP hydrolysis [21]. X-ray and nuclear magnetic resonance (NMR) studies of the SBD complex with the peptide substrate NRLLLTG [19, 22] provided first important insights into the structural basis of substrate specificity and recognition with Hsp70. The initial crystallographic and solution NMR studies of two-domain Hsp70 constructs obtained for *Thermus thermophilus E*. *coli* Hsp70 (DnaK) [23], *Bos taurus* Hsc70 [24], and *Geobacillus kaustophilus* DnaK [25] have puzzled researchers by revealing striking inconsistencies in these structures, particularly differing in the inter-domain arrangement of the NBD and SBD. In the absence of nucleotide, NMR chemical shifts and hydrogen-deuterium exchange of SBD and NBD have shown no changes in a two-domain construct [26]. Subsequent NMR and biophysical investigations of the functional two-domain DnaK construct [27] have confirmed that the NBD and SBD could behave largely independently in the ADP-bound and nucleotide-free states, representing a domain-undocked, linker-unbound structural form of the chaperone (Fig 1). In contrast, ATP binding can stabilize a domain-docked DnaK structure, in which the inter-domain linker binds to a hydrophobic cleft between NBD subdomains IA and IIA, thus promoting the inter-domain communication (Fig 1). This seminal NMR study has offered a molecular view of the biochemical cycle in Hsp70 [27], reconciling inconsistencies seen in the earlier structural studies [23–25]. In the proposed mechanism, the inter-domain linker could act as a main allosteric trigger that is structurally decoupled from the NBD in the ADP-DnaK, while a linker-bound conformation in the ATP-DnaK may stimulate the ATPase activity. Amide hydrogen exchange and mass spectrometry (HX-MS) experiments have explored conformational dynamics of the full-length DnaK and the individual domains in the nucleotide-free and ATP-bound conformation [28]. This study has revealed that global dynamic changes can be characterized by a shearing movement of the four NBD subdomains, high mobility of the SBD-α lid associated with the lid opening/closure, and the increased flexibility of the substrate binding outer loops. Despite a mutual stabilization of the NBD and SBD in the ATP-bound state, the substrate-enclosing loops of SBD can undergo fast exchange with the solvent in the presence of ATP, but not in the ADP-bound state. Similar conclusions have been independently reached by the NMR spectroscopy studies of the ATP-bound DnaK [27]. Collectively, biophysical characterizations of the DnaK dynamics using NMR [27,28], limited trypsinolysis [29], and difference infrared spectroscopy [30] have reaffirmed that ATP binding may promote stabilization of the NBD-SBD interface and ensure protection the inter-domain linker, while inducing the enhanced flexibility in the SBD.

**Fig 2:**
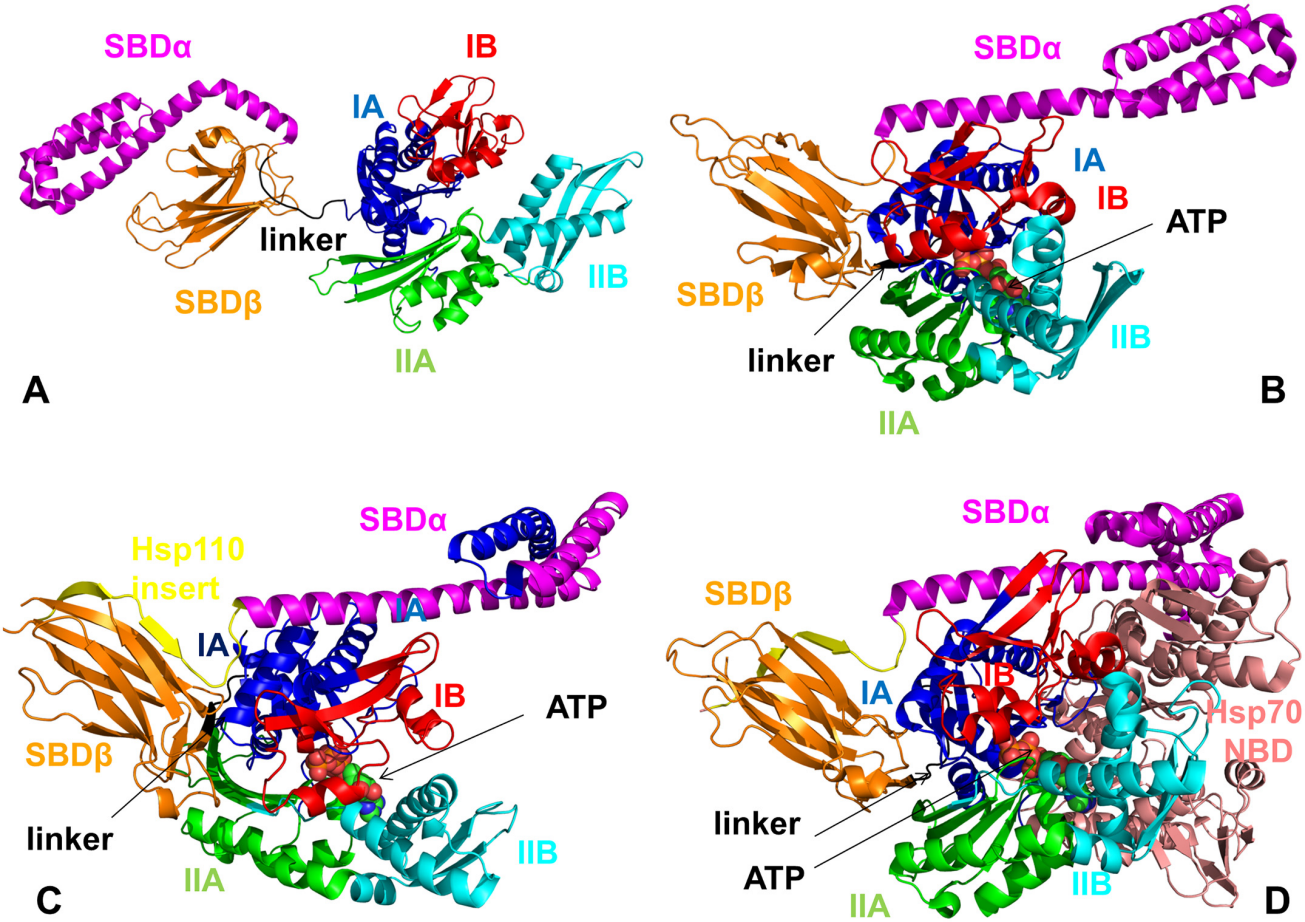
The Structures and Domain Organization of Functional States in DnaK and Sse1p Chaperones. A solution structure of an ADP-bound DnaK (pdb id 2KHO) (A) and the crystal structure of an ATP-bound DnaK (pdb id 4B9Q) (B). The structures are shown in a ribbon representation and main structural elements are annotated. The NBD subdomains are colored as follows: IA (in blue), IB (in red), IIA (in green), IIB (in cyan), the inter-domain linker (in black), SBD-α (in magenta), and SBD-β (in orange). (C) The crystal structure of the yeast Hsp110 (Sse1p) in a complex with ATP (pdb id 2QXL). (D) The crystal structure of Sse1p in a complex with the NBD of hHsp70 (pdb id 3D2F). The structures are shown in a ribbon representation and main structural elements are annotated and colored as in DnaK. The Sse1p insert in Sse1p-ATP structure is shown in yellow (C) and the NBD of hHsp70 in the Sse1p-Hsp70 complex is shown in pink (D).

A significant breakthrough in mechanistic characterization of the Hsp70 chaperone cycle has been made in a series of pioneering structure-functional studies of DnaK that have unveiled for the first time the molecular details of the full-length Hsp70 constructs in the major functional states: nucleotide-free and ADP-bound [31–33], ATP-bound [34,35], and ATP/substrate-bound [36]. The solution NMR structure of the full-length DnaK in the ADP-bound and substrate-bound state (Fig 2A) has confirmed that the NBD, SBD and the inter-domain linker are only weakly bound and move independently, engaging in random collisions on surfaces of the subdomains IA and IIA [31]. NMR studies have also elucidated how ATP binding can facilitate conformational transitions and allosteric communication between radically different functional states. Chemical-shift perturbation patterns for different DnaK states (ligand-bound and apo) and two different inter-domain linker constructs have shown that ATP binding may cause global structural reorganization of the NBD conformation via subtle subdomain motions, bringing the SBD and NBD in a close proximity to enable the inter-domain communication [32]. A solution NMR study of the Hsp70-NBD from *Thermus thermophilus*, in the ADP and AMP-PNP states has identified similar cooperative rotations of the NBD subdomains upon nucleotide exchange [33]. The crystal structures of a full-length two-domain DnaK in the ATP-bound, domain-docked conformation (Fig 2B) were solved independently using different protein engineering strategies to reduce the intrinsic flexibility of Hsp70 and induce favorable crystallization conditions [34,35]. By introducing a disulfide bridge between the SBD-α and NBD, the crystal structure of the ATP-bound DnaK was obtained, in which the SBD-β and the SBD-α subdomains are docked to the NBD [34]. Another crystal structure of an ATP-bound DnaK has been determined with a shortened L_3,4_ loop of the SBD-β [35]. Despite these differences in constructs, the underlying molecular details of the ATP-bound DnaK structures are extremely similar (Fig 2B).

These studies have demonstrated that the extensive NBD-SBD contacts in the ATP-bound state can be accompanied by significant conformational changes in the NBD and SBD regions. The proposed mechanistic model suggested that ATP binding could trigger the insertion of the inter-domain linker into a hydrophobic groove between NBD subdomains IA and IIA, followed by subsequent docking and stabilization of the SBD-β interface with NBD interface, and dynamic binding of the SBD-α lid onto the NBD. NMR experiments combined with substrate-binding anisotropy assays have further probed molecular mechanisms of DnaK by trapping the intermediate (domain-undocked, linker-bound) conformation, that along with the undocked (ADP-bound) and docked (ATP-bound) structures were proposed to constitute major states along the functional Hsp70 cycle [36]. Solution NMR and computational modeling have joined efforts in the most recent *tour-de-force* investigation by unveiling how changes in the internal conformational dynamics of the SBD-β subdomain can play a profound role in the allosteric communication mechanism of the full length DnaK [37]. According to this study, an entropy-driven allosteric mechanism, determined by conformational flexibility of the SBD-β loops, may enable an efficient communication between the SBD-NBD interface and the substrate-binding site.

In the early biochemical studies of Hsp70 chaperones, Mayer and colleagues have first demonstrated that ATP-bound DnaK could be highly dynamic, alternating between a fully open and partially closed conformations in which SBD-α lid may undergo docking and undocking [38,39]. Electron paramagnetic resonance spectroscopy [40] and single molecule fluorescence spectroscopy experiments [41] have confirmed and expanded these findings by unveiling a more detailed and refined picture of the conformational landscape for Hsp70 chaperones. According to these studies, the NBD and SBD were largely flexible in the ADP-bound DnaK and mostly separated, while both domain-docked and the domain-separated states can be sampled in the ATP-bound state. These experiments have shown that at least three conformational states of the SBD (closed, open and intermediate open) may coexist in the presence of ADP. Similar conformational flexibility has been shown for the SBD of BiP, the Hsp70 chaperone in the endoplasmic reticulum [42]. Conformational dynamics studies of the yeast mitochondrial Hsp70 (Ssc1) and DnaK probed by single-pair Förster Resonance Energy Transfer (spFRET) [41,43] have shown that ATP binding may produce a dynamic conformational ensemble in which long-lived, domain-docked conformations may coexist with short-lived, partially undocked intermediates. Isothermal titration calorimetry has elucidated the thermodynamics and energetics of functional DnaK states [44]. This important study has demonstrated a conformational equilibrium between two major conformational ensembles: an ATP-bound state (T), with low affinity for peptide substrates and fast exchange kinetics, and an ADP-bound conformation (R), with high affinity and slow binding and release kinetics. Importantly, an ADP-bound DnaK form is stabilized by both enthalpy and entropy contributions, while ATP binding proceeds with an unfavorable enthalpy change and may be largely driven by the entropic term.

Structural and functional studies have widely employed site-directed mutagenesis and examined the role of specific residues in allosteric regulation of DnaK [45–51]. It has been found that mutations of the NBD residues (P143G, Y145A, N147A, D148A, R151A, R51K, K155D and R167D) can yield DnaK variants that hydrolyze ATP and bind substrate, but lack the inter-domain communication. Mutations of the proline switch residue P143 appeared to increase the rate of transitions between the ATP-like and the ADP-like states, while R151 mutation could completely disrupt the allosteric communication [49]. Mutations in the linker motif 389-VLLL-392 could also impair the inter-domain allostery in DnaK since linker binding to the hydrophobic cleft is critical in the formation of the domain-docked state [50]. A number of functionally important for allosteric regulation sites were discovered in the SBD regions, as mutations of the SBD residues K414I, N415G, P419A and D326V may completely eliminate or significantly reduce allostery [47, 51]. Recent experimental studies systematically analyzed a number of DnaK mutants that could inhibit or alter different steps of allosteric communication, revealing direction-specific pathways and clarifying distinct functional role of critical residues in allosteric regulation [52]. This illuminating study has dissected the previously unrecognized dichotomy of the allosteric control in DnaK by showing that ATP-stimulated substrate release may be more important *in vivo*.

The crystal structure of the yeast Hsp110 (Sse1p), a distant relative of Hsp70s, was solved in a complex with ATP, revealing a similar NBD-SBD docked conformation (Fig 2C) [53]. Sse1 is larger than Hsp70 as the result of insertions within the SBD and a C-terminal extension. The hydrogen-deuterium exchange analysis and site-specific cross-linking analysis of the yeast Hsp110 complex with its yeast Hsp70 partner (Ssa1) have shown that the ATP-induced stabilization of the Hsp110-NBD was considerably stronger than in DnaK, and that stable Sse1p-Ssa1 interactions trigger release of bound ADP from Ssa1[54,55]. The crystal structure of Sse1p in a complex with the NBD of human Hsp70 (hHsp70) has detailed the molecular basis for nucleotide exchange (Fig 2D), in which the ATP-bound NBD and helical subdomain of Hsp110 form a stable interface with the NBD of hHsp70, inducing opening and the release of bound ADP from hHsp70 [56]. In this structure, the hHsp70-NBD is embraced by the NBD and SBD-α of Sse1, leading to the opening of the Hsp70 nucleotide-binding cleft. Unlike Hsp70, the Sse1p subdomain SBD-α does not form a lid over the substrate binding pocket, but instead preferentially interacts with the NBD. This study has confirmed that ATP hydrolysis in Sse1p is not accompanied by significant conformational changes, suggesting that functional cycle of Hsp110 does not employ a population-shift allostery. Fluorescence anisotropy binding assays and binding kinetics experiments have recently shown that unique substrate preferences and extremely fast kinetics for the peptide substrate binding/release in Hsp110 may determine the distinct chaperone activity [57, 58]. It was argued that substrate binding in Hsp110 could still be controlled by ATP, and an allosteric coupling between two functional domains is maintained, albeit in the absence of global conformational changes [57]. These investigations have strengthened the argument that an entropy-driven allostery may represent a primary regulatory mechanism that operates during the Hsp110-Sse1p functional cycle. In this allosteric scenario, the nucleotide binding event is coupled with an efficient substrate binding/release through extensive dynamics changes in the SBD-β domain, and without eliciting large structural changes.

In recent years, computational studies have attempted to quantify mechanistic aspects of allosteric regulation in the Hsp70 chaperones. In early studies, all-atom molecular dynamics (MD) simulations of Hsp70 NBDs have explored conformational changes induced by ATP binding [59]. It was shown that the nucleotide-free NBD could explore a range of subdomain conformations, while the ATP-bound Hsp70-NBD revealed a more restricted conformational ensemble that is reminiscent of the crystallographic structures of ATP-complexes NBDs. Elastic network models and sequence analysis tools have examined structural properties of the Hsp70 complexes with NEFs from different organisms [60]. Mechanistic aspects of allosteric interactions and conformational transitions in Hsp70 and Hsp110 chaperones have been investigated using all-atom MD simulations and elastic network approaches [61], uncovering differences in conformational dynamics of specific protein segments in response to ATP or ADP binding. Nonetheless, the proposed models of signal propagation could not explain the role of conformational dynamics in the substrate binding region as a driver of allosteric changes in Hsp70 and Hsp110. Simulations of the nucleotide-induced conformational transitions in Hsp70 using coarse-grained simulations have directly observed two different types of closing-opening events that involve dissociation and binding of the SBD-α subdomain [62]. Conformational heterogeneity of Hsp70 was also evident in microsecond MD simulations of full-length human Hsp70 in explicit solvent, reproducing major conformational states observed in single-molecule Förster resonance energy transfer (FRET) experiments and small-angle X-ray scattering (SAXS) data of Hsp70 homologs [63]. Unbiased MD simulations and free energy landscape analysis were performed for Hsp70-DnaK in nucleotide-free and nucleotide-bound states, revealing potential conformational pathways and a significant number of mediating residues through which the ATP binding may propagate an allosteric signal to SBD [64]. MD simulations were also combined with mutagenesis, and enzymatic assays to identify functional residues that govern conformational motions in the apo, ATP-bound, and ADP-bound states of DnaK [65]. This study has identified several evolutionally conserved residues that may function as central hinge sites for global motions and whose mutations resulted in a markedly reduced allosteric activities of DnaK *in vitro* and *in vivo*. Using a combination of experimental and computational methods, including *in vivo* functional assays, sequence- and structure-based analyses and perturbation response scanning of DnaK states, a network of conserved residues and interactions in subdomain IA of the NBD was shown to play a critical role in propagating signals between the ATP-binding and substrate-binding sites [66]. Collectively, structural, biophysical and computational studies have demonstrated that nucleotide binding can allosterically affect global conformational rearrangements in the NBD and SBD through modulation of conformational populations and a delicate enthalpy and entropy redistribution.

Protein allostery is determined by the underlying thermodynamics of a system, where ligand-induced redistribution of conformational fluctuations in regions that are linked by cooperative interactions may determine allosteric control and signal transmission [67–73]. Cooperative effects can be positive or negative, depending on whether the binding of the first ligand increases or decreases the affinity for subsequent ligands. Although positive cooperativity is a fairly common mechanism for increasing the binding potential in allosteric systems [74], there have been numerous examples of negative cooperativity in protein systems [75–77]. Negative cooperativity in proteins is mainly entropy-driven and occurs when successive binding to a regulatory site leads to a decrease in affinity and significant redistribution of conformational entropy [71,76,77]. Recent experiments have argued that allosteric regulation of DnaK and Sse1p chaperones may exhibit signs of entropy-driven negative cooperativity, where ATP binding promotes low affinity and fast kinetics of substrate release and association [37,56,57].

The rapidly growing body of biochemical and structural studies has produced a wide array of various hypotheses about nature of allosteric mechanisms that require a critical assessment and should be reconciled in light of most recent experimental developments. Allosteric regulation in Hsp70-DnaK and Hsp110-Sse1p chaperones may couple the intra-domain and inter-domain communications in a complex interaction network that involves a significant number of potentially important functional sites, whose specific roles are not fully understood. Although many aspects of the allosteric regulatory mechanisms in Dnak and Sse1p chaperones have been extensively studied, the atomistic picture of signal propagation and energetics of local conformational ensembles that mediate the allosteric signal still remain unresolved. In particular, it is of significant interest to obtain molecular details of a mechanism by which ATP-induced structural changes could promote the increased dynamics of the substrate binding regions in the SBD-β domain. Conversely, it is not entirely clear at the atomic level how fast substrate release and binding could control energetics of allosteric interactions in the SBD to ensure efficient and robust delivery of the substrate signal to the nucleotide-binding site.

We present a computational framework that combines biophysical simulations, structural stability analysis and network modeling of the DnaK and Sse1p functional states to address some of these questions and obtain an atomistic view of the allosteric mechanisms. We employ an ensemble-based statistical model of allosteric regulation, according to which the dynamics of conformational states and allosteric interaction networks can be modulated and redistributed upon nucleotide and ligand binding [78–83]. This statistical view of protein allostery was recently expanded to studies of allosteric protein interactions in signaling pathways and disease states [84–87], investigations of disordered proteins [88], modeling of molecular networks [89], and mechanisms of allosteric protein inhibition [90]. For network-based analysis of allosteric mechanisms in Dnak and Sse1p chaperones, we employed a graph-based representation of protein structures that can yield a convenient description of residue interaction networks [91–94] and provide a robust framework for understanding allosteric communications. Topology-based network parameters describing node centrality (degree, closeness, and betweenness) have been exploited to predict protein-protein interactions [95,96], ligand binding site [97,98], and catalytic residues in enzymes [99]. These studies have linked organization of protein structure networks with stability and high connectivity of functional residues, particularly indicating that high centrality residues could mediate signaling [100].

In the current study, the molecular basis of allosteric mechanisms in DnaK and Sse1p is elucidated through computational studies of conformational dynamics, protein stability, and residue interaction networks of the chaperone structures. Using structure-based network analysis of residue interactions [93,94] and the force constant analysis of protein stability [101,102], we characterize organization and energetics of the residue interaction networks in functional chaperone states. We show that high centrality residues can be organized in stable communities that mediate allosteric interactions and transmit structural changes in DnaK states. By analyzing the energetics and network properties of the DnaK and Sse1p structures, we show that nucleotide-induced redistribution of local conformational ensembles in the NBD-SBD interface and the SBD-β domain may determine allosteric mechanisms in these systems. The proposed computational approach identifies allosteric hotspots of the chaperone functional cycle that can control the thermodynamics and kinetics of nucleotide-mediated allosteric changes. The results of our study present evidence of highly cooperative interactions and a broad communication network in DnaK structures, while a smaller allosteric network is uncovered in Sse1p structures, which may reflect an entropy-driven mechanism occurring in the absence of global structural changes. By combining MD simulations and binding free energy calculations, we also examine the dynamics and energetics of the peptide substrate interactions with the DnaK structures, revealing key residues involved in control of negative cooperativity between the nucleotide-binding site and the substrate binding region. This study can reconcile a broad range of structural and functional experiments by showing how integration of molecular simulations and network-centric protein modeling may explain thermodynamic and mechanistic aspects of allosteric regulation in the Dnak and Sse1p chaperones.

## Results and Discussion

### Conformational Dynamics and Collective Motions of DnaK and Sse1p Crystal Structures

Crystal structures and NMR studies of DnaK have enabled characterization of major steps and three distinct conformational states (ADP-bound, ATP-bound, and ATP/substrate-bound) of the Hsp70 functional cycle (Fig 1). Allosteric changes between these functional states are reversible and the resulting cycle involves steps of ADP/ATP nucleotide exchange, substrate release, substrate binding, and ATP hydrolysis. The crystal structures of the open and closed DnaK forms have different arrangements of the NBD, SBD-β, SBD-α lid and the inter-domain linker (Fig 2A and 2B). In the ATP-bound DnaK, the NBD rotations of the subdomains I and II induce an opening of the NBD crevice and subsequent insertion of the linker between subdomains IA and IIA. These rearrangements are accompanied by separation and simultaneous docking of the SBD-β and SBD-α subdomain on the NBD, leading to stabilization of the domain-docked conformation and causing a significant increase in rates of substrate association and dissociation (Fig 2B). We simulated DnaK crystal structures to characterize functional dynamics and energetics underlying the inter-domain changes and allosteric interactions during the functional cycle (Fig 1). The objectives of these simulations were a) to describe principal features of the conformational ensembles and characterize the distribution of structurally rigid and mobile regions in allosteric states; and b) to analyze how conformational variability in the ADP- and ATP-bound DnaK structures could induce docking/undocking of the SBD-α and SBD-β subdomains and thus promote global structural changes.

**Fig 3:**
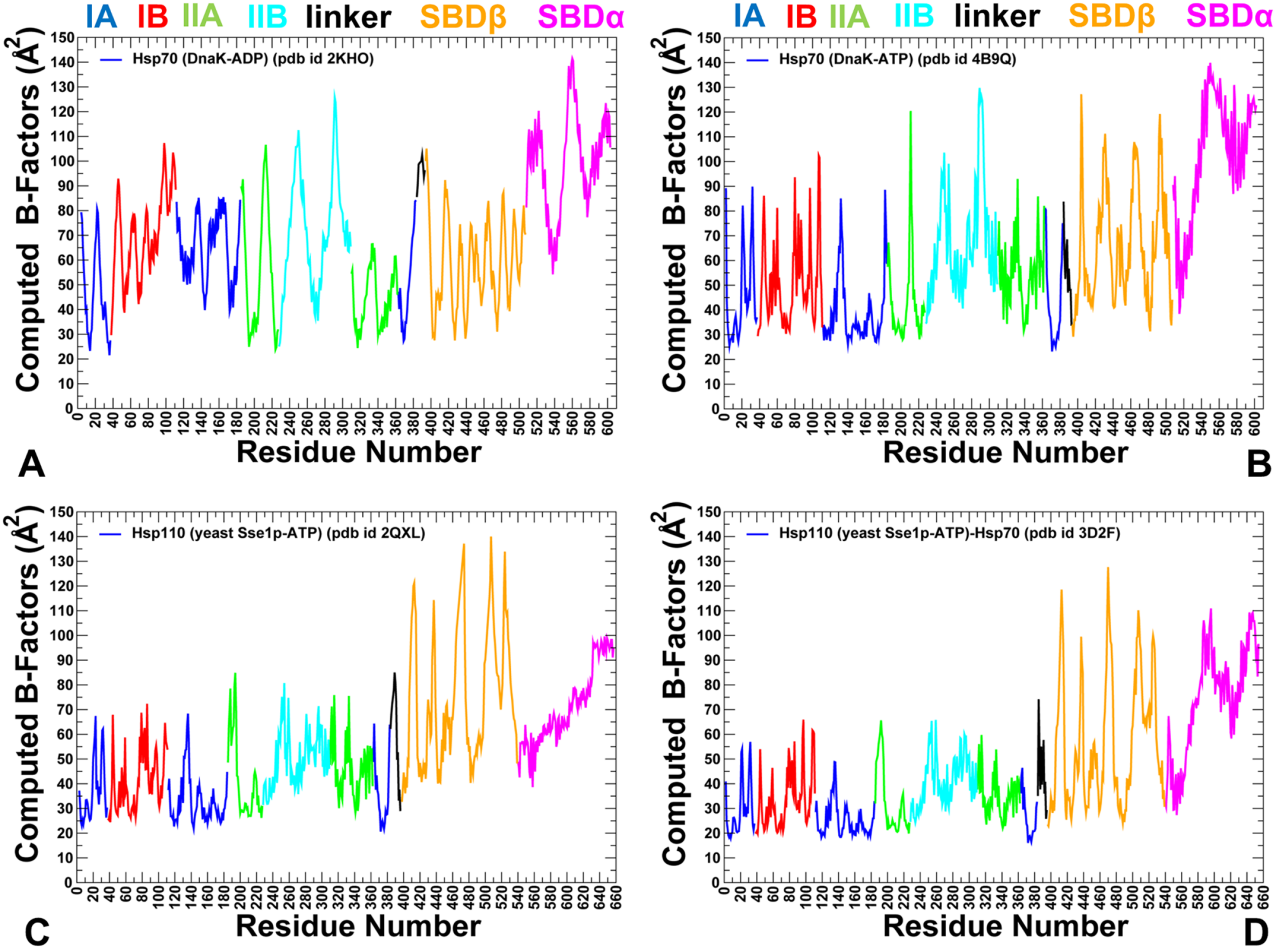
Conformational Dynamics of DnaK and Sse1p Functional States. The computed B-factors obtained from 500 ns MD simulations of the solution structure of an ADP-bound DnaK (pdb id 2KHO) (A); the crystal structure of an ATP-bound DnaK (pdb id 4B9Q) (B); the crystal structure of a Sse1p-ATP (pdb id 2QXL) (C); and the crystal structure of Sse1p in a complex with the NBD of hHsp70 (pdb id 3D2F) (D). The thermal fluctuations are shown only for DnaK and Sse1p residues (B-factors of the hHsp70-NBD counterpart of Sse1p are omitted for clarity and uniformity of presentation). Equilibrium residue fluctuations are annotated and colored according to the adopted coloring scheme of the chaperone subdomains: IA (in blue), IB (in red), IIA (in green), IIB (in cyan), the inter-domain linker (in black), SBD-α (in magenta), and SBD-β (in orange).

All-atom MD simulations of DnaK in the ADP-bound (Fig 2A) and ATP-bound forms (Fig 2B) (500 ns for each system) were combined with the structural stability analysis to quantify conformational changes induced by the nucleotide binding. To provide a direct comparison of conformational ensembles for DnaK and Sse1p chaperones, we also conducted 500 ns MD simulations of the crystal structures of the ATP-bound Sse1p [53] and ATP-bound Sse1p complex with the NBD of human Hsp70 [56]. In simulations of the ADP-DnaK, we observed a significant conformational heterogeneity that was manifested across all subdomains (Fig 3A). Conformational fluctuations were observed in the NBD subdomains, with especially large movements in the subdomain IIB and the SBD-α subdomain (often termed as the α-helical “lid”). Although the SBD-α lid remained stably bound to the SBD-β subdomain during MD simulations of the ADP-DnaK, we detected appreciable, though rather short-lived excursions of the lid, largely involving rigid body motions around the SBD-β (Fig 3A). In these conformations, the SBD-α explored different lid orientations with respect to the SBD-β, probing intermediate states that were previously observed in solution NMR studies [31], electron paramagnetic resonance spectroscopy [40] and single molecule fluorescence spectroscopy [41]. Conformational dynamics of the ADP-DnaK also revealed larger movements of subdomains IIB and SBD-α as evident from the computed B-factors (Fig 3A). In the ATP-bound DnaK, conformational dynamics was characterized by smaller fluctuations of the NBD residues, reflecting structural tightening of the ATP-binding site and the NBD core (Fig 3B).

Conformational dynamics of the ADP-DnaK revealed shear movements [103] of the SBD-α lid around the SBD-β, as these subdomains displayed a relative movement along the plane of their original interface (with some minor twisting of ~ 10–12 degrees) (S1 Fig). However, the time scale of simulations was insufficient to observe large-scale movements seen in the NMR studies [37]. The experimentally observed see-saw and shear motions would likely to yield large conformational transitions between the DnaK structures on a longer time scale, since residual dipolar coupling obtained in NMR studies of DnaK showed that the NBD and SBD can move in cones of ±35° with respect to each other [31]. We also observed that rotations of the subdomain IIB in the NBD may be accompanied by shear and sliding-twist movements of the SBD-α lid around the SBD-β (S1 Fig). These results are consistent with chemical shift perturbations data that detected rotational dynamics of subdomain IIB and partial opening of the nucleotide-binding cleft in the ADP-bound DnaK [32]. The relevance of subdomain IIB motions for DnaK allostery was stressed in various experiments [32–34] and computational studies [60, 64]. Our simulations depicted a considerable heterogeneity of the lid conformations and the entire conformational ensemble of the ADP-DnaK (S1 Fig). Both crystallographic and intermediate-closed states were sampled on the simulation time scale, confirming that docking of the SBD-α with the NBD may be highly dynamic. These results are consistent with the recent electron paramagnetic resonance spectroscopy analysis of DnaK transformations [40] and FRET measurements of the conformational dynamics in mitochondrial Hsp70-Ssc1 [41]. According to these experiments, the ADP-DnaK state can be rather heterogeneous with respect to the domain-domain interaction distances and the degree of the SBD opening.

Despite the prevalence of the domain-docked conformations in the ATP-DnaK ensemble, we observed dynamic movements of the SBD-α that may deviate from its docked position and weaken contacts with the NBD. Although the ATP-Dnak structure exhibited smaller fluctuations in the NBD, the subdomain IIB and SBD-β loops L_1,2_ (residues 404–406), L_3,4_ (residues 428–434), and L_5,6_ (residues 458–473) surrounding the substrate-binding site (S2 Fig) showed a considerable flexibility in the ATP-bound form. At the same time, L_2,3_ loop (residues 412–420), a portion of the L_4,5_ loop (residues 439–457) and L_6,7_ loop (residues 479–482) displayed moderate thermal fluctuations. These results supported experimental observations [36,37], suggesting that the ATP-DnaK form may be quite dynamic, especially near the L_1,2_ and L_3,4_ loops, allowing for undocking movements even in the absence of substrate. Furthermore, according to the NMR data [36, 37], simultaneous action of ATP and substrate binding may stabilize a population of domain-undocked, allosterically active states. Despite different structural arrangements of ADP-bound and ATP-bound DnaK forms, conformational dynamics of these allosteric states revealed a comparable degree of intrinsic mobility. These results are consistent with biophysical studies of DnaK structures [44], in which the differences in conformational stability and dynamics between allosteric DnaK states were found to be relatively small, indicating that these conformations must be thermodynamically and kinetically accessible during the functional chaperone cycle.

Conformational dynamics of the DnaK structures showed a considerable heterogeneity that could arguably promote structural transitions between allosteric states and determine a population-shift allosteric mechanism. Due to the ensemble nature of allostery [104–107], a rigorous description of the population-shift mechanism and a quantitative characterization of the nucleotide-induced redistribution of states require full exploration of the conformational ensembles and statistically significant free energy landscapes. Despite limitations associated with the time scales of all-atom MD simulations for large biological assemblies, we found that molecular simulations captured fairly well conformational heterogeneity of DnaK states. The density of states profiles that were obtained from conformational ensembles of the ADP-DnaK (S3 Fig) and the ATP-DnaK structures (S4 Fig) provided evidence of a population-shift between the thermodynamically stable crystal structures and functionally significant intermediates. This analysis confirmed that each nucleotide-bound form may populate diverse conformational ensembles [38,39] in which the interfaces between the SBD-α lid and the SBD-β (for ADP-DnaK) and between the SBD-α and the NBD (for ATP-DnaK) can be perturbed and trigger a cascade of allosteric transformations. In the ADP-DnaK ensemble, a population of states was detected for a partially undocked intermediate ensemble in which the SBD-α lid may depart from the SBD-β–SBD-α interface (S3 Fig). In these intermediate states, the movements of the α-helical lid may induce the initial formation of the SBD-β-NBD interfacial contacts and trigger remodeling of the inter-domain linker. In the ATP-bound DnaK, we also observed elements of a population-shift between the crystallographic domain-docked structure and a domain-undocked intermediate. In these conformations, the SBD-α could partly undock from the NBD, which is accompanied by rotation of the NBD subdomains from each other, remodeling of the inter-domain cleft, and weakening of the linker-NBD contacts (S4 Fig). These results reproduced several key elements of a population-shift mechanism in DnaK, confirming that the allosteric structures at both ends of the functional cycle could be dynamic and populate domain-undocked intermediate conformations independent of the nucleotide status. These findings are consistent with the NMR studies [38, 39] and biophysical measurements [40–42] that demonstrated a mobile nature of the ATP- DnaK.

Conformational dynamics of ATP-bound Sse1p (Fig 3C) and Sse1p-hHsp70 structures (Fig 3D) highlighted important differences in the mobility of DnaK and Sse1p chaperones. Although crystal structures of ATP-Dnak and ATP-Sse1p are very similar, the dynamics signatures of these forms appeared to be noticeably different. All NBD subdomains in Sse1p, including subdomain IIB, remained stable in MD simulations. Notably, conformational flexibility of the SBD-α lid was considerably reduced in the ATP-bound Sse1p (Fig 3C and 3D). According to our results, conformational dynamics of ATP-Sse1p is characterized by dense regions of high structural stability (NBD and the NBD-SBD interface) and isolated “islands” of conformational mobility in the substrate binding loops. In Sse1p, the NBD subdomains form tight rigid modules with only minor relative motions. Intriguingly, conformational dynamics of the SBD-β subdomain showed significant fluctuations (Fig 3C and 3D). In Sse1p structures, the substrate binding loops L_1,2_ (407-DKQVEDE-413) and L_3,4_ (436-TGD-438) have vastly different sequences and length as compared to DnaK, where these loops have three (404-MGG-406) and seven (428-TAEDNQS-434) residues respectively. These significant differences in loop sequences may contribute to chaperone-specific dynamic changes that were observed in simulations. The distribution of rigid and flexible regions in Sse1p structures showed an excessive exchange of local flexibility in the SBD regions that may be contrasted with the lack of global structural changes. Hence, differences in the conformational dynamics of DnaK and Sse1p chaperones may be associated with the redistribution of rigidity and flexibility in key functional regions, which may ultimately determine the allosteric mechanism in these systems.

**Fig 4:**
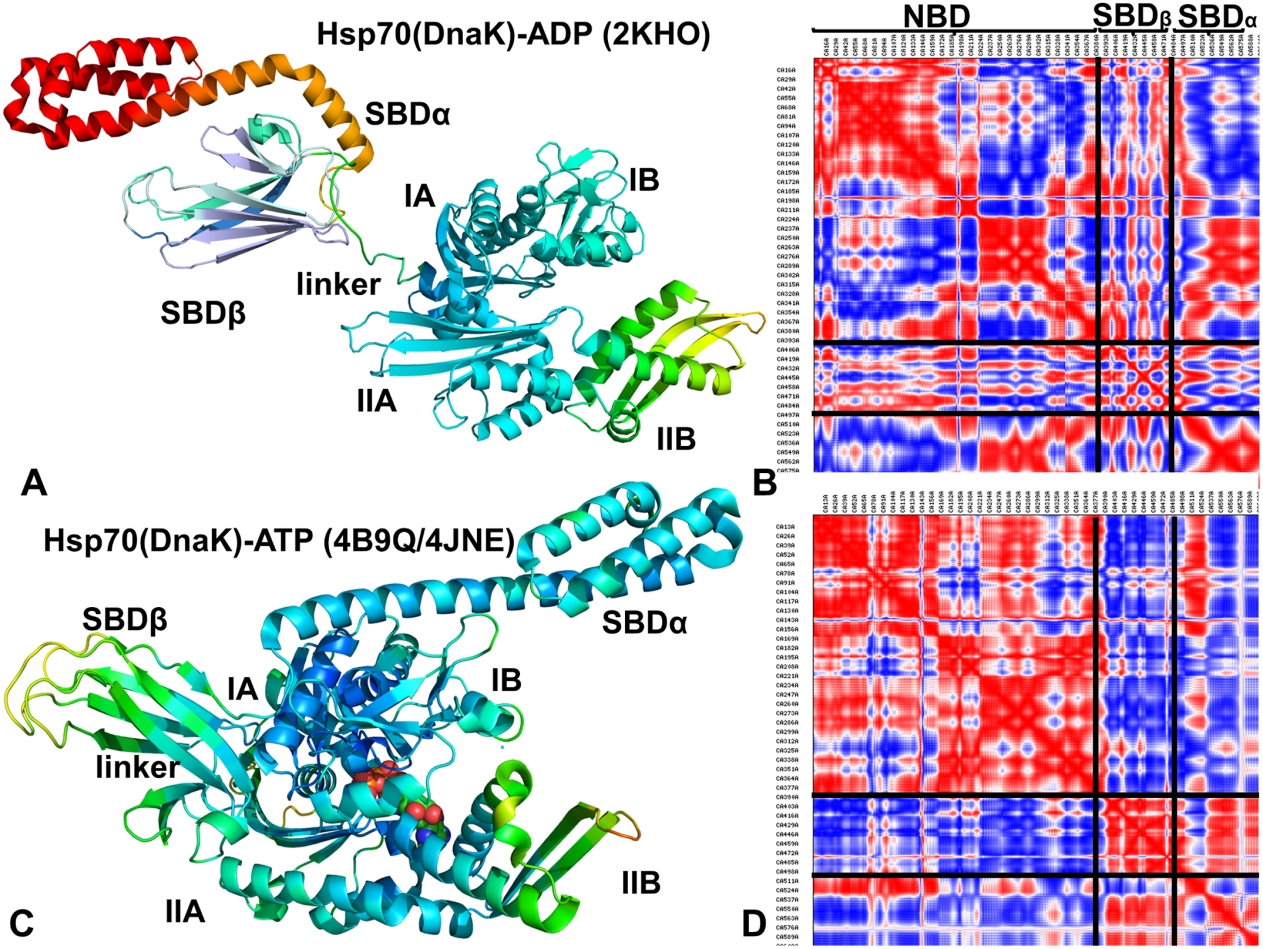
Analysis of Essential Motions in the Closed and Open DnaK Forms. Functional dynamics maps and cross-correlation matrices of residue fluctuations for the ADP-bound DnaK structure (A, B) and ATP-bound DnaK form (C, D). Conformational dynamics profiles were computed by averaging protein motions in the space of three lowest frequency modes. The color gradient from blue to red indicates the decreasing structural rigidity of the protein residues. PCA computations are based on the Cα atoms. The axes denote Cα atoms of the protein residues in sequential order. Cross-correlations of residue-based fluctuations vary between +1 (fully correlated motion; fluctuation vectors in the same direction, colored in red) and -1 (fully anti-correlated motions; fluctuation vectors in the same direction, colored in blue). The residue ranges corresponding to the NBD, SBD-α, and SBD-β regions are highlighted.

**Fig 5:**
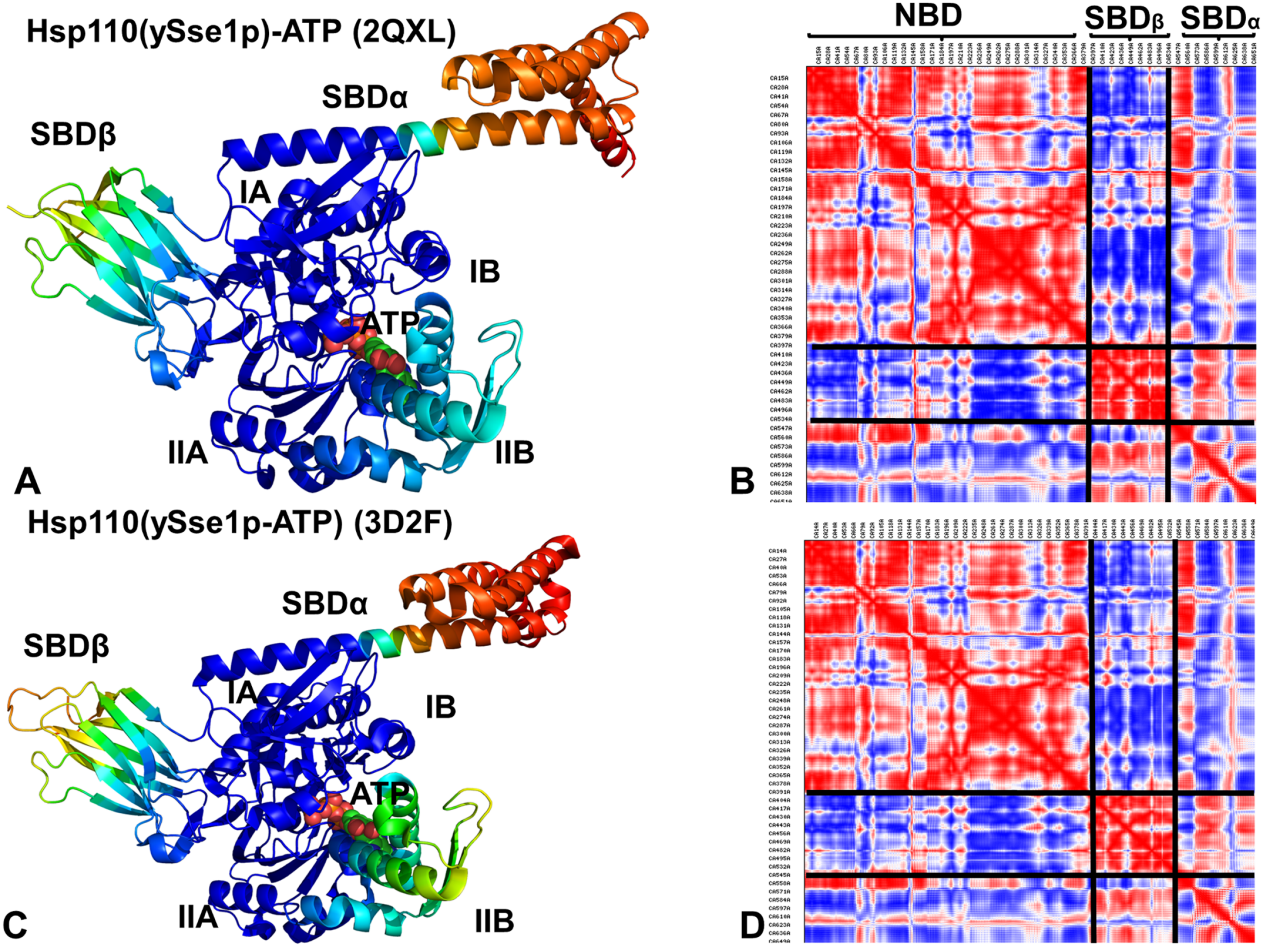
Analysis of Essential Motions in the Sse1p Structures. Functional dynamics maps and cross-correlation matrices of residue fluctuations for the Sse1p-ATP structure (A, B) and Sse1p-hHsp70 structure (C, D). Conformational dynamics profiles of Sse1p were computed by averaging protein motions in the space of three lowest frequency modes. In (C, D) the plots depict the dynamics profiles for Sse1p structure only. The corresponding profile of the hHsp70-NBD counterpart of Sse1p in the complex is omitted for clarity of presentation. The color gradient from blue to red indicates the decreasing structural rigidity of the protein residues. PCA computations are based on the Cα atoms.

Principal component analysis (PCA) of simulation trajectories in the essential space of low frequency modes clarified differences in global collective motions of these chaperones. In the DnaK structures, the NBD subdomains were not excessively rigid, and the subdomain IIB along with the SBD-β substrate binding loops was quite flexible (Fig 4A and 4B). Functional dynamics maps of residue cross-correlations showed an appreciable coupling of the NBD subdomains induced by ATP binding (Fig 4C and 4D). Another interesting observation was the emergence of positive cross-correlations between the SBD-α and SBD-β residues in the ATP-bound DnaK. As a result, cooperative motions of the SBD subdomains may promote stability of the NBD-SBD interactions and enable nucleotide-dependent allostery in DnaK. Subtle but important differences in functional dynamics emerged from simulations of the ATP-bound Sse1p structures (Fig 5). We noticed that structural core of Sse1p, most notably the NBD subdomains, may become exceedingly rigid. Furthermore, movements of the SBD-α and SBD-β subdomains may become increasingly decoupled, owing to a contrasted pattern of rigidity and flexibility of stably bound SBD-α and highly flexible substrate binding loops of the SBD-β (Fig 5A and 5B). A similar picture was observed in the Sse1p-hHsp70 complex, where the presence of bound hHsp70-NBD did not change the dynamics map of Sse1p (Fig 5C and 5D). We argue that differences in collective movements may be associated with allosteric scenarios that are adopted by DnaK and Sse1p chaperones.

We also analyzed conformational flexibility of DnaK and Sse1p structures by constructing residue-based deformability profiles. From a perspective of rigidity theory [108–110], protein flexibility can be associated with the ability to readily deform residues near functional regions that enables global structural transitions between allosteric states. Using the normal mode analysis (NMA) in internal dihedral coordinates and all-heavy atoms representation [111,112], we generated residue-based deformability profiles for DnaK and Sse1p structures with the aid of iMODS server [113] (S5 Fig) The distribution of high deformability peaks pointed to locations of hinge sites or “molecular joints” that could transmit the collective motions and allosteric signal. In these profiles, hinge sites may be attributed to regions where high mobility residues are located next to structurally rigid segments. Interestingly, the distribution of hinge positions was quite different in DnaK and Sse1p. A significant number of local hinge sites that are broadly distributed across all regions, including both NBD and SBD subdomains, were found for both closed and open forms of DnaK (S5 Fig). This distribution is reflective of a large and robust allosteric interaction network with multiple hinge sites, in which long-range signal can be propagated through cooperative structural changes. In contrast, for Sse1p structures, only a small number of such hinge-like positions were found and they were located in the NBD subdomain IB and near the NBD-SBD-α interface. As a result, collective motions in Sse1p structures may be somewhat restricted and rely on a small number of hinge points. Since motions that transmit allosteric signal in Sse1p structures are relatively minor, the lack of diverse network of “molecular joints” may produce a smaller allosteric network and preclude nucleotide-dependent allostery.

Noteworthy, the time scale of all-atom MD simulations employed in the current study is still insufficient to fully explore conformational ensembles of the chaperones and reconstitute the complete free energy landscapes of allosteric transitions between fully open and closed DnaK structures. Nonetheless, the results of simulations provided evidence of a population-shift between the DnaK crystal structures and functionally relevant meta-stable intermediates. Recent computational studies of ATPase motors have employed a battery of sophisticated and powerful sampling tools to model allosteric function of these proteins that translocate a long substrate through their central pore powered by ATP hydrolysis [114]. By utilizing advanced path sampling techniques, such the string method with swarm of trajectories [115,116], and milestoning analysis [117] in combination with all-atom MD simulations, this study has produced accurate free energy profiles that depicted a population-shift in conformational states of homohexameric helicase Rho that is coupled to translocation of the mRNA substrate. Conceptually similar to the allosteric mechanisms of Hsp70 chaperones, allosteric hotspot residues involved in the subunit−subunit interactions in this system can modulate the relative thermodynamic stability of conformational states and thus control translocation of the mRNA substrate. The free energy landscapes of allosteric proteins can be also evaluated using simplified protein models [118] and metadynamics sampling approach [119,120] that allows for accelerated observation of rare events by choosing effective path collective variables. This technique was successfully applied to study ligand-induced allosteric transitions in protein kinases [121] and conformational changes in drug-protein complexes [122]. We are currently exploring the feasibility of these sampling techniques for all-atom molecular simulations of molecular chaperones. However, this work extends beyond the scope and objectives of the current study and will be presented elsewhere.

### Protein Stability Analysis of the DnaK Structures: Functional Residues Allosterically Modulate Stability and Dynamics of the SBD-β Domain and Substrate Binding Loops

**Fig 6:**
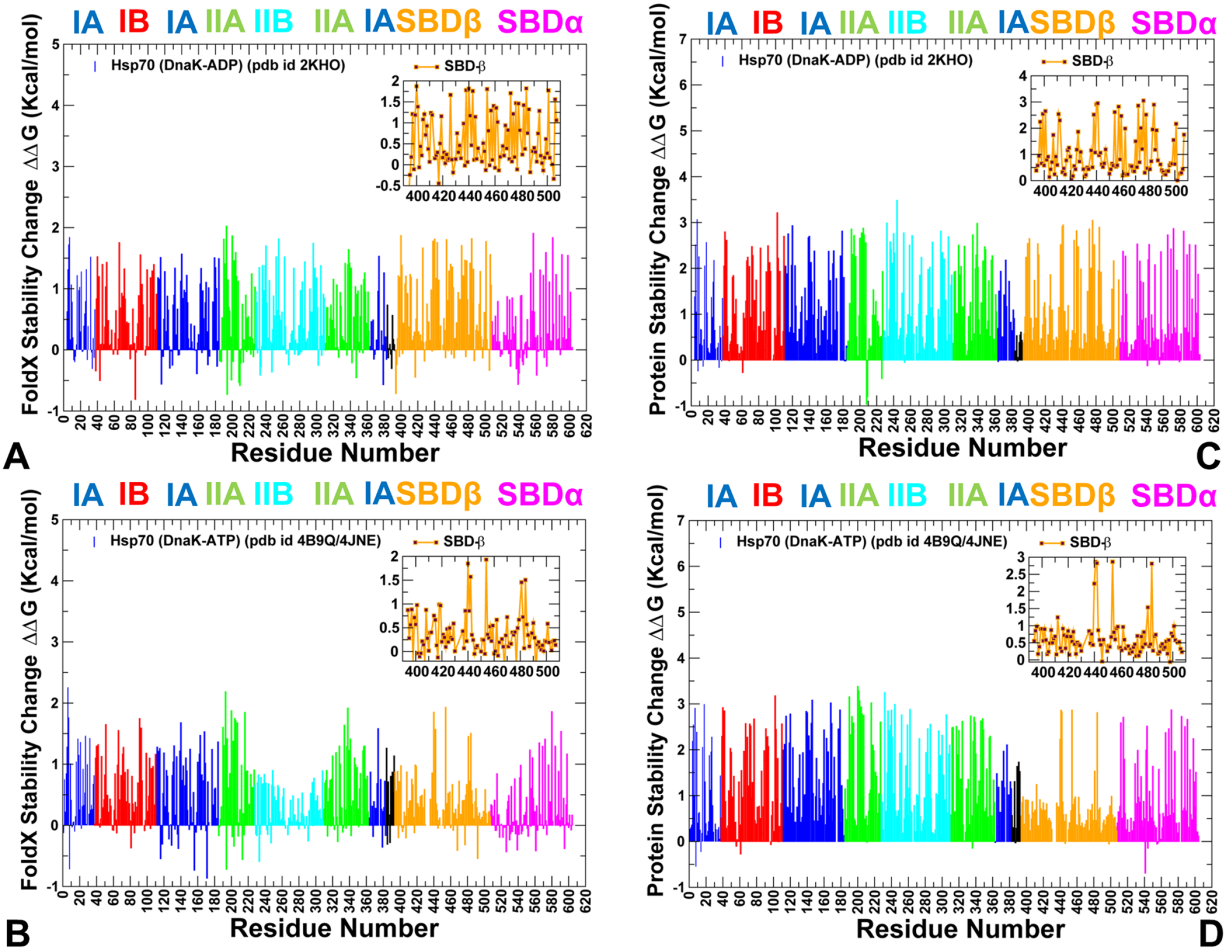
The Protein Stability Analysis of DnaK Structures. Protein stability changes ΔΔG are computed using a systematic alanine scanning. The protocol involved modification of the protein residues to alanine and computing the effect of each mutation on protein stability using FoldX (A,B) and DUET (C,D) methods respectively. The profiles are annotated using residue numbering in the solution structure of the ADP-bound DnaK (pdb id 2KHO) for (A, C) and the crystal structure of an ATP-bound DnaK (pdb id 4B9Q) for (B, D). The profiles are shown as bars colored according to the adopted scheme: IA (in blue), IB (in red), IIA (in green), IIB (in cyan), the inter-domain linker (in black), SBD-α (in magenta), and SBD-β (in orange). The inset shows protein stability changes of the SBD-β residues (orange-colored lines with marron-colored filled squares).

We analyzed allosteric interactions in the DnaK structures from two different but complementary points of view. According to a mechanistic-based model, allosteric mechanism is described as nucleotide-induced signal propagation from one site to another via a series of conformational changes and intermediate structures. In the ensemble-based model of allostery [104–107], ligand binding or mutations capable of altering the energetic hierarchy of states in the thermodynamic ensemble can change the allosteric coupling between two allosteric sites, even when the network of allosteric residues that physically connects these two sites remains largely unaffected. To explore the energetic determinants of the ensemble-based allostery, we computed protein stability changes by using a systematic alanine scanning of the DnaK structures, in which the free energy changes were averaged over MD-based conformational ensembles. Of particular interest was to quantify the energetics of allosteric coupling between the NBD-SBD interface and highly flexible substrate binding loops in the ATP-DnaK, which is arguably central to the allosteric structural changes [37]. We also examined how fast substrate binding in the ATP-DnaK could control energetics of allosteric interactions to ensure efficient transmission of the substrate signal to the NBD-SBD interface and the nucleotide-binding site. The protein stability changes in DnaK structures were computed using the FoldX approach [123,124]. In a systematic alanine scanning of DnaK residues we utilized a graphical user interface for the FoldX force field calculations [125] that was implemented as a plugin for the YASARA molecular graphics suite [126]. If a free energy change between a mutant and the wild type (WT) proteins ΔΔG = ΔG (MT)-ΔG (WT) > 0, the mutation is destabilizing, while when ΔΔG <0 the respective mutation is stabilizing. FoldX could be sensitive to conformational changes in the MD ensembles, and the crystal structure is typically more suitable for stability predictions that any single snapshot. To ensure reproducibility of the free energy computations and still consider functionally important dynamic changes, we computed the average ΔΔG values using multiple samples (~100–200) from stable equilibrium ensembles using a modified FoldX protocol [127,128]. Moderate destabilizing free energy changes were distributed across all subdomains, with a relatively small number of sites producing a significant destabilizing effect upon mutation (ΔΔG >1.5–2.0 kcal/mol) (Fig 6A and 6B). Moreover, the pattern of protein stability changes in the open and closed DnaK structures was quite similar, which is consistent with similar free energies associated with these allosteric states [44]. These results are consistent with the experimental observations in which melting temperatures of the WT DnaK and a number of important DnaK mutants were rather similar [52], indicating that even mutations of functionally critical for allosteric communication residues could not cause global structural defects and markedly impair stability of the protein fold. Noteworthy, however, mutations of allosterically important residues in the NBD (K71, E171, P143, Y145, F146, D148A, R151, K155 R167, I168) and SBD-β (I438, V440, L454, L484) may still incur a moderate but noticeable destabilizing effect (Fig 6A and 6B). Accordingly, structural perturbations exerted on these residues could redistribute local conformational ensembles and alter energetics of functional regions that are involved in ligand-based allostery. Notably, these changes would not compromise structural integrity of DnaK folds or significantly affect the total energy, as the ligand-induced cooperativity is different from cooperativity related to folding/unfolding transitions. We also computed the protein stability changes (Fig 6C and 6D) using an alternative computational approach DUET [129]. This machine learning method produces a consensus prediction by integrating the results of mutation Cutoff Scanning Matrix (mCSM) algorithm that relies on structural signatures of protein residue environments [130] and Site Directed Mutator (SDM) method that is based on a statistical potential energy function [131]. Despite differences between the FoldX and DUET approaches, these methods revealed comparable protein stability changes, though DUET predictions resulted in the larger free energy differences. More importantly, both approaches displayed similar patterns of protein stability changes in the DnaK regions.

We found that mutations of functional residues may weaken local interactions between allosterically coupled elements involved in cooperative changes. In particular, mutations of functional residues may alter stability of such important interaction clusters as K70-E171-P143, N170-R151-D481, R167-I168-K155-D481, Q442-D148-L454-L484 that are involved in the NBD-SBD interactions and inter-domain communication (S6 Fig). Consequently, local structural perturbations in these sites could modulate conformational ensembles around pivotal points of allosteric coupling without compromising thermodynamic stability of the protein fold. Importantly, alanine mutations of the hydrophobic residues in the SBD-β subdomain: I438 (strand β4), V440 (strand β4), L454 (strand β5), L484 and V486 (strand β7) could lead to significant stability changes, whereas other SBD-β residues remained less susceptible to such modifications (Fig 6). The importance of these hydrophobic residues was noted in the NMR studies [37], where it was suggested that the SBD-β hydrophobic core (L454, L484 and also I501 from β8) forms a critical allosteric hotspot for propagating dynamic changes through the entire SBD-β domain. Consistent with these experimental revelations, we found that the relative energetic contribution of the β4/β5/β7 core residues was considerably more important in the ATP-DnaK structure (Fig 6B). At the same time, mutations in the substrate binding loops L_1,2_ (residues 404–406), L_3,4_ (residues 428–434), and L_5,6_ (residues 458–473) produced only small changes, reflecting the enhanced conformational variations and instability of these regions. In the ATP-DnaK ensemble, the hydrophobic residues V440, L454, L484 form a stable interacting cluster that is supported by the contacts with Q442 and D148 (S6 Fig). These residues form an indispensable core of the SBD-β that is linked via Q442 and D148 with the NBD interface and controls thermodynamics of allosteric coupling with the substrate binding loops. Indeed, even small hydrophobic changes L454I and L484I could redistribute the conformational ensemble of the ATP-DnaK and significantly increase the population of the partially undocked conformations with the addition of the substrate [27]. The protein stability analysis is also consistent with the functional studies [52] that detected the loss of substrate-induced stimulation of the ATPase activities in V440A, L484A and D148A mutants.

In the ATP-DnaK, the SBD-β core is directly linked with a pair of structurally stable residues from the substrate binding region (I438 and F426) that form a hinge region that allows L_1,2_ and L_3,4_ loops to move freely and enjoy the increased flexibility (S6 Fig). The substrate binding loop residues involved in the “hydrophobic arch” over the substrate site (typically measured by the distance between M404 from L_1,2_ and A429 from L_3,4_ [37]) could sample multiple orientations and tend to favor wide-open arch orientations. In the ADP-DnaK, the hydrophobic SBD-β cluster is structurally rearranged and weakened, while the I438 and F426 residues become partially separated (S6 Fig). These structural changes may contribute to redistribution of the conformational ensemble and the reduced mobility of the substrate binding loops. Noteworthy, the L_3,4_ loop segment (428-TAEDNQS-434) in one of the ATP-DnaK structures (pdb id 4B9Q) is longer than in the other structure (pdb id 4JNE) where the L_3,4_ loop is replaced by a short construct (428-MGG-430). Consequently, the L_1,2_ and L_3,4_ loops are more flexible in the first structure, whereas in the second construct a wide-open substrate binding cavity may be enforced by steric constraints imposed on a shorter L_3,4_ loop (S6 Fig).

To summarize, these results suggested that nucleotide-induced allosteric changes in DnaK could be controlled by the stability of the hydrophobic SBD-β core (V440, L454, L484) that is linked with the NBD-SBD interface (Q442,D148) and the substrate binding loops (F426, I438). The protein stability analysis showed that structural rigidity of the SBD-β hydrophobic core in the ATP-DnaK may be linked with the dynamic changes in the substrate binding loops, and not only influenced by the absence/presence of substrate. These findings can be interpreted in the context of the ensemble-based model, where allosteric effects of structural perturbations between remote protein regions could emerge due to redistribution of the conformational ensemble [104–107]. In the thermodynamic ensemble of the SBD-β conformations, structural stabilization of the SBD-β core is negatively coupled with the dynamics and energetics of the substrate binding loops, which may provide a mechanism for fast substrate release and binding.

**Fig 7:**
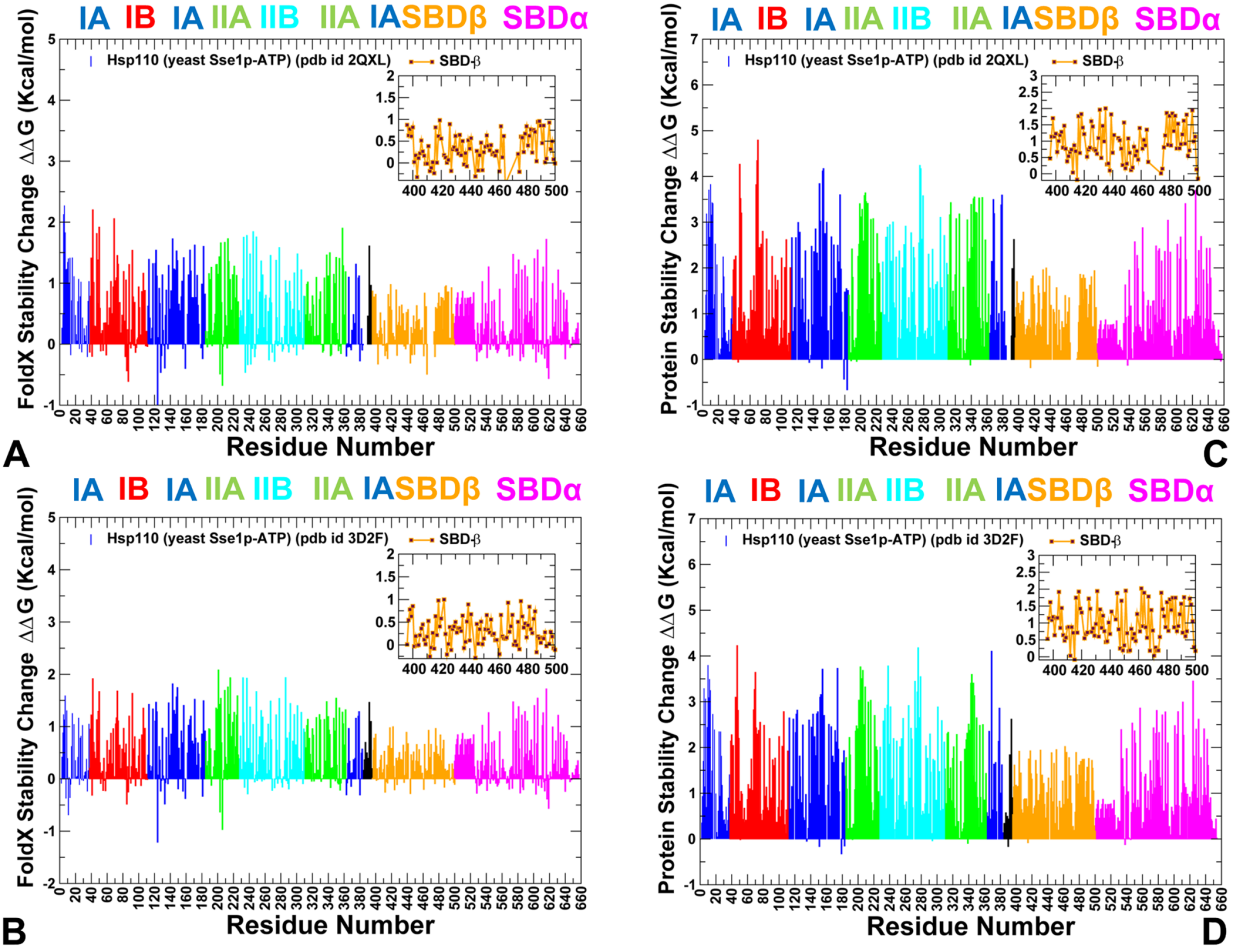
The Protein Stability Analysis of Sse1p Structures. Protein stability changes ΔΔG are computed using a systematic alanine scanning. The protocol involved modification of the protein residues to alanine and computing the effect of each mutation on protein stability using FoldX (A,B) and DUET (C,D) methods respectively. The profiles are annotated using residue numbering in the crystal structure of ATP-Sse1p (pdb id 2QXL) for (A,C) and the crystal structure of ATP-Sse1p in a complex with the NBD of hHsp70 (pdb id 3D2F) for (B,D). The profiles are shown as bars colored according to the adopted scheme as in Fig 6.

Similarly to Dnak, mutations of functional residues in the Hsp110 (Sse1p) produced rather moderate changes in protein stability using both FoldX (Fig 7A and 7B) and DUET methods (Fig 7C and 7D). This is consistent with the experiments in which the CD spectra and thermal melting curves of the Sse1p mutants were similar to the Sse1p-WT in structure and stability [53]. Nonetheless, protein stability residue scanning in the Hsp110 (Sse1p) ATP-bound structures showed noticeably smaller free energy changes for the SBD-β residues as compared to other regions. This energetic analysis reflected the markedly enhanced dynamics of the substrate binding loops in Sse1p and the increased levels of structural rigidity in the SBD-β hydrophobic core residues. Our results also supported previous experimental hypotheses [57, 58], according to which nucleotide-based allosteric communication in Sse1p could be a primary example of entropy-driven cooperativity. This mechanism occurs through redistribution of local conformational ensembles and extensive dynamics exchanges in the SBD-β domain regions, without producing significant structural changes [94].

### Molecular Dynamics Simulations and Free Energy Calculations of Substrate Binding to DnaK Structures: Allosteric Hotspots of Negative Cooperativity

Negative cooperativity can underlie nucleotide exchange in DnaK, in which ATP binding induces a significant reduction in the substrate affinity and leads to high rates of substrate release and association, while ATP hydrolysis restores the high-affinity substrate state [37]. To determine the energetic determinants and allosteric hotspots of negative cooperativity, we conducted MD simulations of the DnaK structures in the presence of 7-residue peptide substrate NRLLLTG [19, 22]. These simulations examined the effect of peptide binding on conformational flexibility of the substrate binding site and energetics of the substrate-DnaK interactions. Using MD simulations of the ADP- and ATP-bound states in the presence of the NRLLLTG peptide, we performed binding free energy calculations and alanine scanning of the substrate binding site residues. Computational alanine scanning approach first proposed by late Peter Kollman [132,133] can estimate the energetic contribution of each residue to the total binding energy through systematic alanine modifications by employing the molecular mechanics (MM) force field [134] combined with the generalized Born and solvent accessible surface area (GB/SA) solvation model [135,136]. Using this protocol, we evaluated the energetic role of the substrate-interacting residues on binding affinity using MD trajectories of the WT complexes and MM-GBSA calculations.

**Fig 8:**
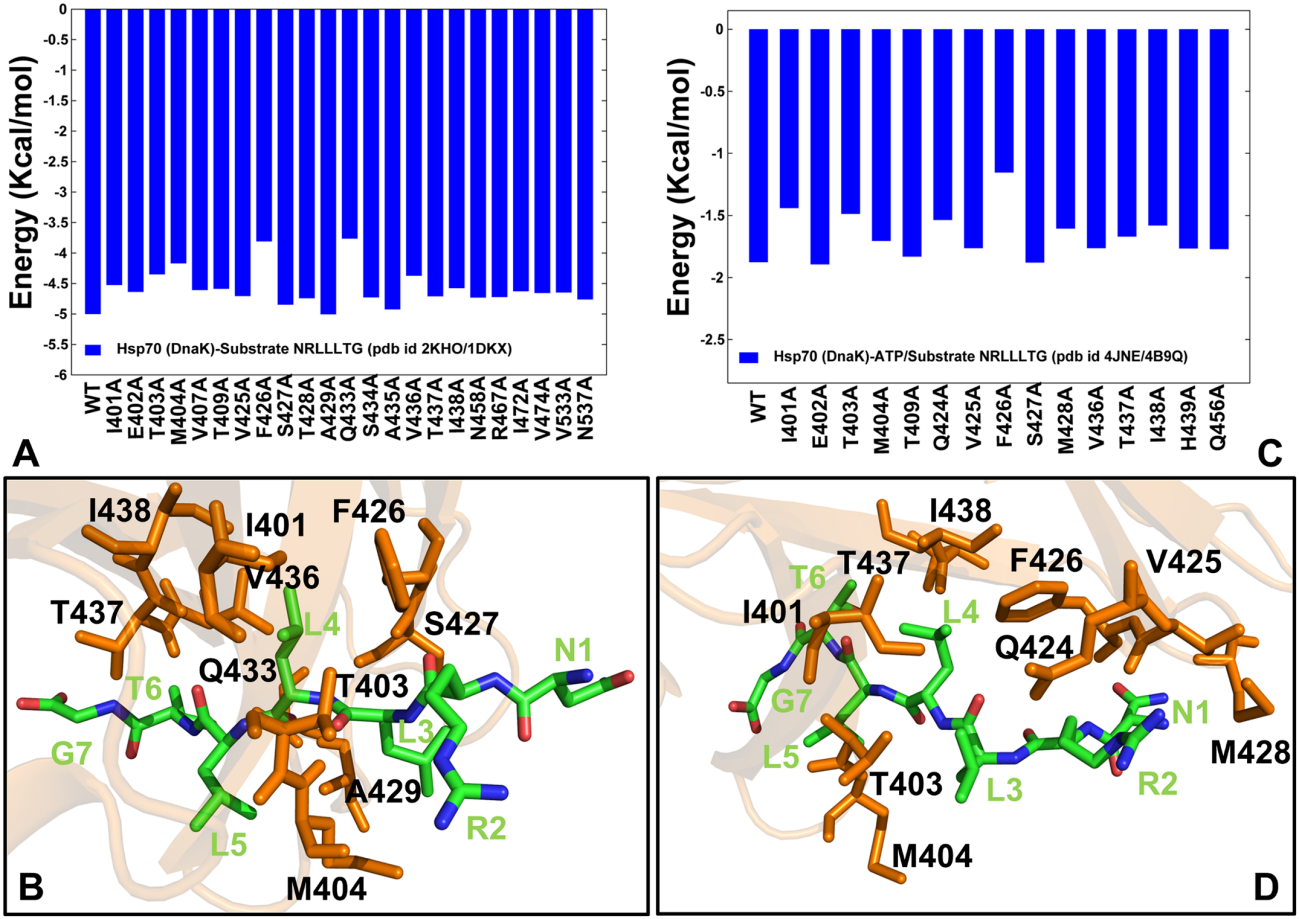
Free Energy Calculations of the Substrate Binding with the DnaK Structures. Binding free energies and alanine scanning of the NRLLLTG substrate-interacting residues with the ADP-DnaK (A) and ATP-DnaK (C). Computational alanine scanning employed MM-GBSA calculations to evaluate the effect of alanine mutations for the substrate binding site residues on binding affinity using MD trajectories of the nucleotide/substrate-bound WT structures. The protocol involved a systematic modification of the inhibitor-interacting residues to alanine by eliminating side-chain atoms beyond C_β_, and measuring the effect of each mutation on binding affinity. The close-up of the NRLLLTG substrate binding mode and interacting residues is shown for ADP-DnaK (B) and ATP-DnaK (D). The peptide is shown in atom-colored sticks and annotated. The substrate binding site residues from the SBD-β subdomain are shown in orange sticks and annotated.

According to the crystallographic and NMR structure of the substrate-bound DnaK-SBD [19, 22], in the high affinity state the peptide binds in an extended conformation interacting with the SBD-β and SBD-α subdomains. The substrate binding mode and interactions are largely retained in the solution structure of complete DnaK construct complexed with [31]. We initiated simulations of the ADP/substrate-bound DnaK using the SBD-bound crystallographic conformation of the NRLLLTG peptide [19]. In the course of simulations, the substrate maintained the stable bound conformation locked in the crystallographic position through a dense network of van der Waals interactions and hydrogen bonds with the SBD domain (Fig 8A and 8B). The NRLLLTG peptide binding pocket is formed by residues from loops L_1,2_ and L_3,4_, but also stabilized by additional contacts with L_4,5_ and L_5,6_. The computed binding free energies registered an appreciable substrate affinity, also revealing the key contributions of I401, T403, M404, F426, A429, Q434, and V436 residues that could act as energetic hotspots of the substrate binding (Fig 8A). A significant role of these residues in determining binding energetics reflected substrate-induced stabilization of the L_1,2_ (residues 404–406) and L_3,4_ (residues 428–434) loops. These results are consistent with biochemical studies of substrate-DnaK binding in which M404A, V436F, and A429W mutants displayed > 10-fold loss of substrate binding affinity [38,39]. Mutagenesis studies have also shown that two single-site mutations, F426S and S427P could lead to the weakened peptide binding affinity [47]. Hence, binding free energy calculations reaffirmed that the substrate binding affinity may be predominantly determined by the interactions with specific hydrophobic residues in the L_1,2_ and L_3,4_ loops.

We also examined the role of the SBD-β hydrophobic core residues on substrate binding. While F426 is a critical hotspot of substrate binding in the ADP-DnaK state, I438 may have only small effect on binding affinity (Fig 8A). The contributions of the SBD-α residues V533 and N537 were also relatively minor. This may explain why the removal of the helical subdomain in DnaK could produce a construct that retains the ATP-induced peptide substrate release [21]. Our findings also confirmed the key role of hydrophobic Leu3 and Leu4 residues in the peptide substrate (Fig 8B). Of special importance is Leu4 residue that forms hydrophobic contacts with F426, V436, I401, T403, and I438 that are further supported by hydrogen bonds with A429 and Q433. Additionally, Leu3 makes contacts with M404, A429 and S427 residues in the substrate binding site. Hence, the binding site residues interacting with the Leu3 and Leu4 of the peptide could determine the substrate binding affinity, confirming DnaK preference for the aliphatic residues in these positions [57].

Simulations of the substrate binding with the ATP-bound DnaK structure revealed a considerable peptide mobility and suggested a plausible binding mode of substrate recognition (Fig 8C and 8D). Although the substrate contacts with the L_1,2_ and L_3,4_ loops were dynamic, the substrate recognition mode may still rely on Leu3 and Leu4 positions to form initial contacts with I401, T403, Q434, F426, and I438 residues in the SBD-β (Fig 8D). The observed similarities in the peptide binding modes and interaction patterns may be determined by sequence-specific substrate recognition requirements. However, the free analysis revealed weaker substrate-DnaK interactions and a reduced binding affinity of the substrate to the ATP-DnaK (Fig 8C and 8D). This may reflect negative cooperativity in DnaK in which ATP binding can allosterically induce the decreased affinity of substrate binding. Intriguingly, these results may provide some useful insight into a mechanism by which substrate recognition may trigger fast and robust signal transmission to the NBD-SBD interface and nucleotide-binding site. In particular, we observed that the Leu4 substrate position may establish recognition contacts with F426 and I438 residues (Fig 8D). In the previous section it was suggested that this pair of residues may contribute to the hinge in the SBD-β that controls the mobility of the substrate binding loops. Furthermore, F426 and I438 are directly connected with the hydrophobic core of the SBD-β (V440, L454, and L484) that is central for allosteric coupling between SBD-β and the nucleotide-binding site (S6 Fig). We argue that the substrate recognition could send signal through contacts with the F426/I438 residues that may be propagated via allosteric cascade provided by the SBD-β core, and leading to an efficient signal transmission with the NBD interface. This mechanistic picture that is based on the binding free energy analysis is consistent with the recently proposed mechanism of substrate-induced stimulation of the ATPase activity [52]. Based on mutagenesis experiments, this study suggested that allosteric communication from the substrate binding pocket to the NBD may be triggered by initial substrate contacts with I438, and subsequent signal transmission through V440, L484 and D148 residues. The binding free energy analysis confirmed that the peptide contacts (Leu4) with F426 and I438 residues may be critical for the initial substrate recognition. It may be also argued that structural stability of the SBD-β core residues would ensure the efficiency and robustness of signal propagation from the substrate recognition site to the NBD-SBD interface. Hence, substrate binding may be linked with specific DnaK residues and well-defined communication routes for allosteric signaling. These findings reconcile important experimental studies [37,52], confirming an important role of the SBD-β core as a central hub of allosteric interactions between the NBD and substrate binding site.

### Nucleotide-Induced Modulation of Conformational Stability in DnaK and Sse1p Chaperones: A Comparison with Hydrogen Exchange Experiments Suggests Entropy-Driven Allosteric Changes

Conformational stability profiles of allosteric states in DnaK and Sse1p chaperones were also compared with the hydrogen deuterium exchange (HDX) experiments. In these experiments, the amide groups that are most protected from solvent exchange display the largest stabilities. Residues with low protection (or stabilities) may readily undergo amide exchanges with solvent due to local fluctuations and structural changes in the native structures. Experimental measurements of hydrogen exchange rates are usually confined to the backbone amide protons that also depend on hydrogen bonding strength and local dynamics, and therefore slowly exchanged amide groups may not necessarily be deeply buried. Hence, the experimentally observed changes in the HDX profiles may depend on both structural stability and the extent of residue burial. Residue depth (RD) is a measure of solvent exposure that can determine not only whether a residue is exposed or buried, but can also quantify the depth of a residue from the protein surface [137–139]. Previous studies have established that RD values correlated with the extent of conformational fluctuations and protein stability changes, residue packing density and measured HDX profiles [140–142]. To characterize energetics of ligand-induced structural changes and facilitate comparison with the HDX data, we combined protein stability analysis with the ensemble-based RD profiling of chaperone structures.

**Fig 9:**
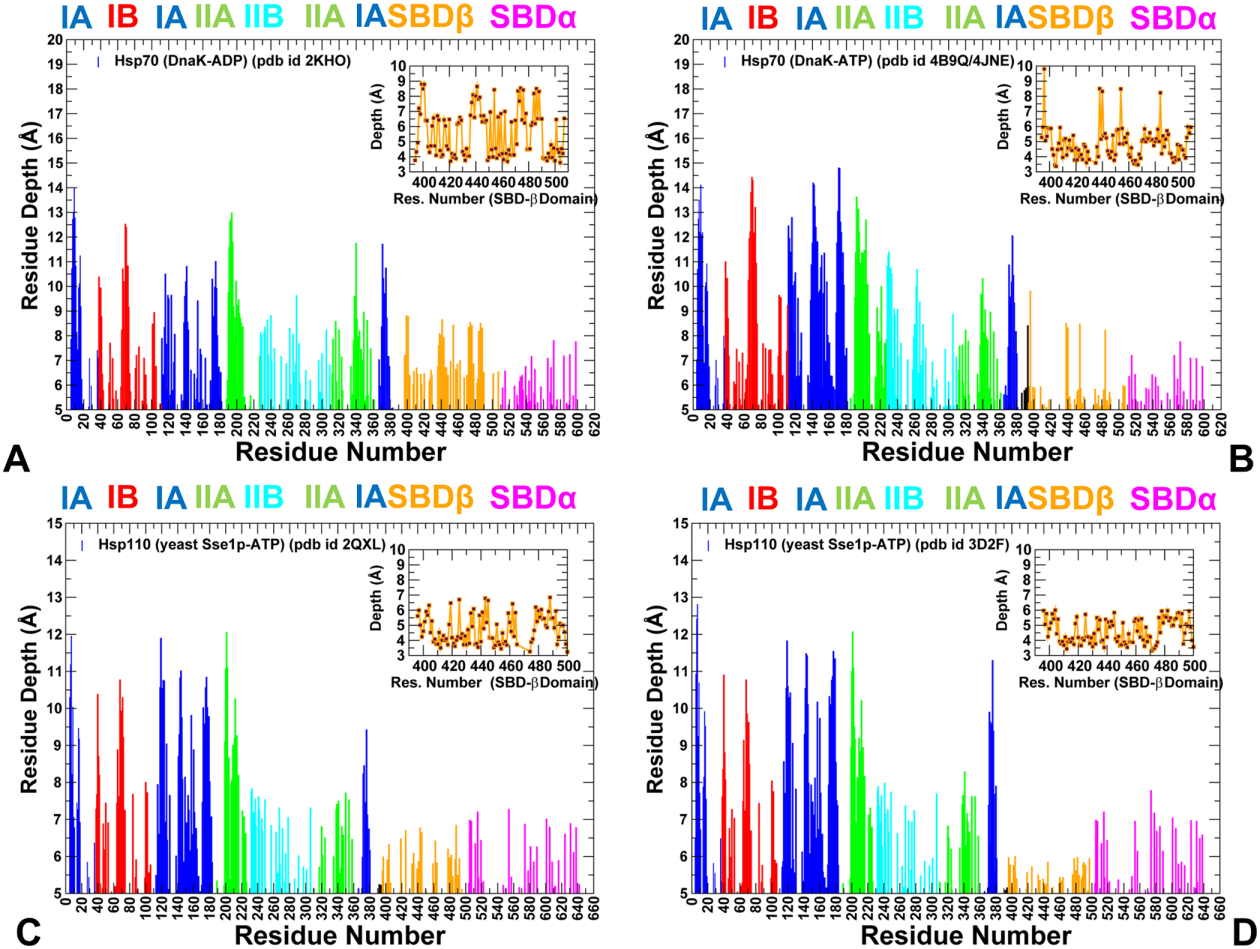
The Residue Depth Profiling of DnaK Structures. The RD profiles are shown for ADP-DnaK (A), ATP-DnaK (B), and ATP-Sse1p structures (C, D). The RD values were obtained by averaging computations over the respective equilibrium ensembles. The profiles are annotated using residue numbering in the solution structure of the ADP-bound DnaK, (pdb id 2KHO) (A) the crystal structure of an ATP-bound DnaK, (pdb id 4B9Q) (B), the crystal structure of ATP-Sse1p (pdb id 2QXL) (C) and the crystal structure of ATP-Sse1p in a complex with the NBD of hHsp70 (pdb id 3D2F) (D). The profiles are shown as bars colored according to the adopted scheme: IA (in blue), IB (in red), IIA (in green), IIB (in cyan), the inter-domain linker (in black), SBD-α (in magenta), and SBD-β (in orange). The inset in each panel shows protein stability changes of the SBD-β residues (orange-colored lines with marron-colored filled squares).

**Fig 10:**
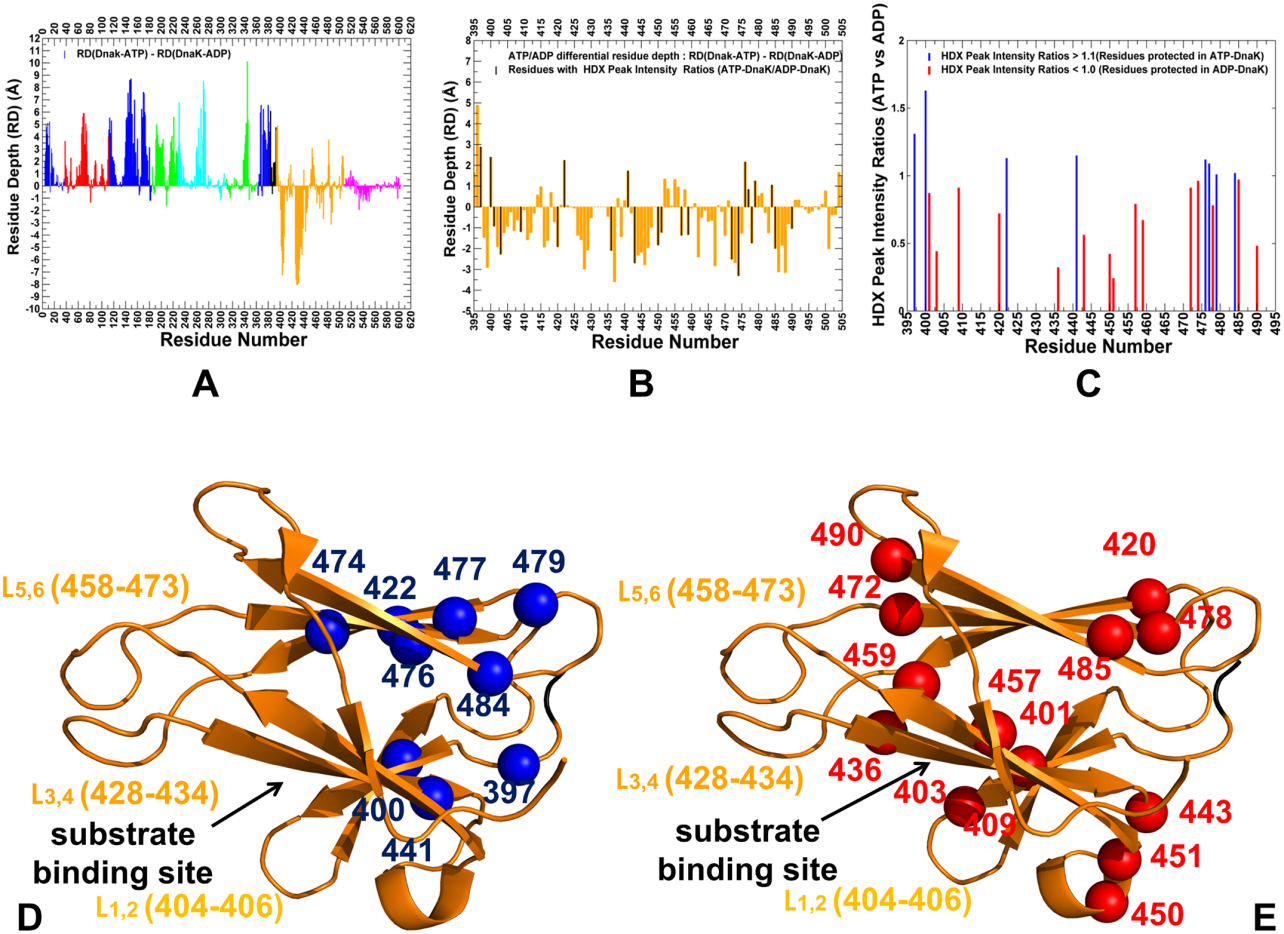
The Residue Depth Profiles in the DnaK States: Structural Mapping and Comparison with the HDX Experiments. The computed RD profiles are compared with the HDX experiments, showing nucleotide-induced protection of functional regions. The effect of nucleotide binding is evaluated using differential plots of the RD profiles between the ATP-DnaK and ADP-DnaK (A). The profiles are shown as bars and colored according to the adopted scheme as in Fig 8. (B) The differential RD values are shown only for the SBD-β subdomain (orange bars) and RD values for residues with the experimentally known HDX peak intensity ratios between ATP-DnaK and ADP- DnaK are highlighted in marron bars. (C) The experimental values of the HDX peak intensity ratios between ATP-DnaK and ADP-DnaK [27]. Residues that are more protected in the ATP-DnaK (HDX peak ratio > 1.1) are shown in blue bars, and residues that are more protected in the ADP-DnaK form (HDX peak intensity ratio < 1.0) are shown in red bars. This annotation is based on the HDX peak assignments as prescribed in [27]. (D). Structural mapping of the ATP-DnaK protected residues depicted in blue spheres and annotated. (E) Structural mapping of the ADP-DnaK protected residues shown in red spheres and annotated. The SBD-β subdomain is shown in orange ribbons. The substrate binding loops L_1,2_ loop (residues 404–406), L_3,4_ loop (residues 428–434), and L_5,6_ loop (residues 458–473) are indicated.

The RD profiles in DnaK structures (Fig 9A and 9B) showed the larger values and greater protection level for subdomains IA, IB, and IIA, mostly for stable regions near the nucleotide binding site and the inter-domain interfaces. According to this analysis, the average RD values of the SBD-β residues were generally smaller, especially in the domain-docked ATP-DnaK (Fig 9B) reflecting the enhanced dynamics and structural changes of substrate binding loops of the SBD-β. Instructively, the RD values of the hydrophobic core residues I438, V440, L454 and L484 were much larger, indicating that these residues remained structurally stable and protected from solvent exchange. To provide a comparison with the HDX data [27, 28], we analyzed the differential RD profiles that reveal which residues could be more protected in the ATP-bound or ADP-bound forms. The high RD values (greater protection) of the NBD residues and small RD values (low protection) of the substrate binding loops was observed in the ATP-DnaK (Fig 10A). According to the HDX experiments, the SBD fragments 413–437 and 439–457 exchanged amide protons more rapidly in the ATP-bound state as compared with the nucleotide-free DnaK form [28].

To make a residue-specific comparison with the HDX experiments, we mapped differential RD values with the residue peak intensities between ATP- and ADP-pulsed samples of DnaK. In the HDX experiments, this metric quantified the relative amount of hydrogen exchange in the presence ATP versus ADP [27]. The RD average values for individual residues in the SBD-β domain accurately reproduced the experimentally observed protection (stabilization) changes induced by the nucleotide binding (Fig 10B and 10C). In particular, the ATP/ADP differential depth values were positive for L397, G400, H422, V440, L441, F476, D477, D479, L484 residues (Fig 10B). These residues displayed the larger average depth in the ATP-bound DnaK, suggesting that they would be better protected from solvent and more stable. Accordingly, these residues produced experimental resonances that were more intense after a pulse of ATP as compared to ADP (Fig 10C and 10D), i.e. these sites become more protected in the ATP-DnaK form [27]. Interestingly, these results also confirmed structural stability of the SBD-β residues (V440, L454, and L484) in the ATP-DnaK. Structural stability of the surrounding residues (L441, F476, D477, and D479) could provide an additional layer of protection for the hydrophobic core. Hence, functional residues that are important for allosteric signaling could be shielded by stable neighboring residues that could ensure the robustness of signal transmission in the dynamic protein environment.

On the other hand, our analysis revealed negative ATP/ADP depth differences for I401, T403, T420, V436, G443, D450, N451, F457, L459, I478, and D490 residues (Fig 10B). These residues are situated near the substrate binding loops L_1,2_, L_3,4_ and L_5,6_ substrate binding sites (Fig 10E) that are subjected to significant structural fluctuations and therefore exhibited the reduced protection in the ATP-DnaK. Indeed, in the HDX experiments, these residues were less intense in the ATP-pulsed sample (Fig 10C and 10E), that reflected their destabilization and low protection in the ATP-bound state [27]. To summarize, the residue-based depth profiling correctly detected the protection patterns in the DnaK structures, and closely mapped these changes onto the respective residues. Computational predictions supported the notion that significant structural fluctuations and low protection status of the substrate binding loops in the ATP-DnaK may be a consequence of entropy-driven allosteric changes. More broadly, our results suggested that the nucleotide-induced redistribution of conformational ensembles in DnaK structures could be controlled through negative cooperativity and enthalpy-entropy exchange between rigid and flexible regions, provided that the protein structure tends to restore the global balance between rigidity and flexibility within the native ensemble [143,144]. According to this scenario, the enhanced stabilization of the SBD-NBD interface and the SBD-β hydrophobic core in the ATP-DnaK may elicit the greater flexibility of the substrate binding loops. This mechanism is also reminiscent of an entropy–enthalpy transduction (EET) concept [145], in which conformational shifts between allosteric states may promote measurable enthalpy-entropy exchanges in regions that are remote from the perturbation site.

The residue-based depth profiling of the Sse1p structures (Fig 9C and 9D) revealed high RD values of the NBD core residues (i.e. high protection level and stability) that can be contrasted with much smaller RD values of the SBD-β residues, indicative of significant structural fluctuations in this region. Similarly to our analysis of DnaK, we mapped the RD values against the HDX measurements of Sse1p [54, 55]. While the NBD exhibited strong protection in the ATP-Sse1p, the nucleotide presence had a small deprotection effect in the SBD-β segments 395–403 to 521–533 [55]. At the same time, the α-helical lid showed local stabilization of segments 534–550, 551–563, and 577–585 that are involved in the interfacial contacts with the NBD [55]. Significant differences in the average residue depth for the NBD and SBD-β regions reflected a more pronounced segregation of rigid and flexible regions in the Sse1p structures. In particular, we detected a low level of solvent protection (i.e. small RD values) for L_1,2_ (residues 407–413) and L_3,4_ loops (residues 436–438) and L_5,6_ substrate binding sites. Additionally, a significant number of highly flexible SBD-β residues with low solvent protection level were detected in the “rift” between the NBD and SBD-β domains (S7 Fig) that is uniquely characteristic of the Sse1p structures. In this context, it is worth noting that the precise location of the peptide binding site in Sse1p is still unresolved. In fact, besides binding cavity between the L_1,2_ and L_3,4_ loops, another substrate binding site may be located in the NBD-SBDβ inter-domain “crack” [56, 57, 146]. These findings indicated that the substrate binding regions in Sse1p may be characterized by the increased dynamics and low protections (or stabilities). Hence, this computational analysis supported the notion that entropy-driven allosteric mechanism in Sse1p may occur due to redistribution of local conformational ensembles and significant entropy exchanges in the SBD-β regions.

### Probing Protein Mechanics of DnaK States: A Balance of Structural Rigidity and Flexibility Dictates Allosteric Mechanism

Conformational dynamics of functional chaperone states provided a platform for an in-depth analysis of conformational populations and identification of conserved hinge regions and deformation patterns. A balance of rigidity and flexibility in functional states is required for efficient transmission of conformational changes, as excessively rigid systems have difficulties to adapt and largely flexible systems may be unable to maintain robustness of the allosteric communication. By synergistically combining the force constant approach [101,102] and network centrality analysis [93, 94], we conducted a comprehensive analysis of rigidity and flexibility in the DnaK and Sse1p structures. In the force constant approach, the fluctuations of the mean distance between each residue and the rest of the protein are converted into force constants to measure the energy cost of the residue deformation during simulations. Residues exhibiting minimal displacements along low frequency motions are considered as hinge sites. According to previous studies [147–149], an efficient allosteric communication may be determined by rigid coupling of local hinge sites that propagate cooperative protein movements and transmit allosteric changes. In the dynamics-based network analysis, we employed residue betweenness as a global centrality measure of the residue interaction networks. The betweenness (centrality) of a residue node is defined as the number of shortest paths that pass through that node in the protein structure network, representing a global measure of the node contribution to the communication within the network. Throughout the text, we interchangeably use terms “residue centrality” and “residue betweenness”. The residue centrality can characterize and differentiate highly connected residues that mediate stable interaction networks and allosteric communications in protein structures [95–100].

**Fig 11:**
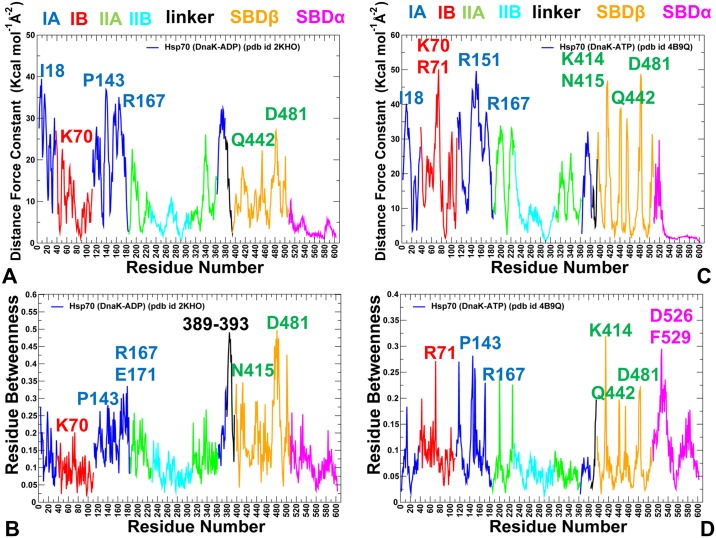
Force Constant and Network Centrality Profiles of DnaK Forms. Residue-based force constant profiles and network centrality distributions for an ADP-bound DnaK form (A, B) and ATP-bound Dnak state (C, D). The profiles are annotated and colored according to the adopted scheme: IA (in blue), IB (in red), IIA (in green), IIB (in cyan), the inter-domain linker (in black), SBD-α (in magenta), and SBD-β (in orange). The residue-based dynamic profiles are annotated using the residue numbering in the solution structure of an ADP-bound DnaK, (pdb id 2KHO) and the crystal structure of an ATP-bound DnaK, (pdb id 4B9Q). The peaks of the force constant and centrality profiles corresponding to functionally important residues are indicated by arrows and annotated.

**Fig 12:**
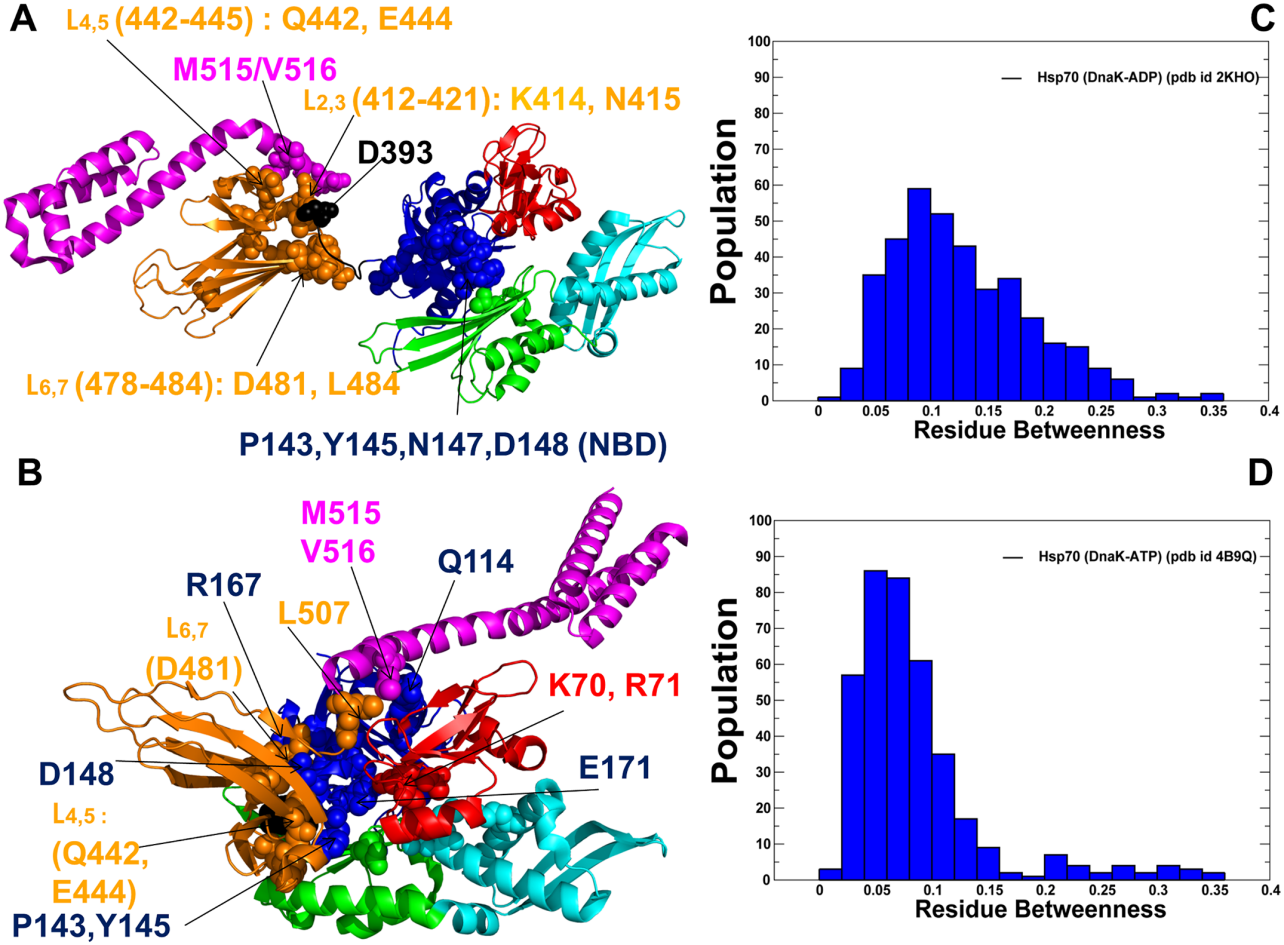
Analysis of High Centrality Residues in DnaK States. Structural mapping of common peaks in the force constant and network centrality distributions onto DnaK conformations (A, B). The structures are shown in a ribbon representation and main structural elements, including subdomains and functional residues are annotated and colored according to the adopted scheme. The functional residues of high centrality are shown in spheres and colored according to their respective subdomains. Structural positions of high centrality functional residues are indicated by arrows. The probability distributions of residue centrality in the ADP-bound (C) and ATP-bound DnaK forms (D). These profiles were obtained from MD trajectories by averaging computations of residue centrality over all protein residues in the conformational ensembles.

Using conformational ensembles, we computed the average betweenness indices and considered sharp peaks in the residue centrality profiles as a guiding indicator for the identification of functional residues critical for allosteric regulation. Strikingly, the force constant and centrality profiles in the DnaK forms (Fig 11) revealed generally similar distribution patterns, with the corresponding peaks often pointing to the same residues. Moreover, functional residues that were characterized by high force constants typically exhibited high network centrality. Structural mapping of high force constant (high centrality) residues onto DnaK conformations (Fig 12A and 12B) further clarified their functional role. We observed that hinge sites with high network centrality may be involved in key inter-domain contacts and form an interaction network that may propagate allosteric signals. In the ADP-bound DnaK, both the force constant profile (Fig 11A) and the centrality distribution (Fig 11B) featured numerous local peaks that were broadly distributed across domains. The main peaks corresponded to residue clusters 140–151 (subdomain IA), residues 371–373 (subdomain IA3), linker residues 393–394, residues 413–417 (L_2,3_ loop), residues 440–444 (L_4,5_ loop) and residues 479–482 (L_6,7_ loop) (Fig 11A and 11B). These distributions pointed to two potential hinge sites: the first corresponded to the interface between IA and IIA subdomains, while the second one was situated at the border between structurally rigid subdomain IA and a more flexible linker region.

In the ATP-bound DnaK, we observed a greater number of high peaks in the subdomain IA, IB, IIA of NBD and the SBD-β subdomain (Fig 11C and 11D). The multiple peaks included subdomain IA residues 7–15, 140–154, 161–175 (Fig 12B) that displayed high force constant values, which is consistent with a complete protection of these regions in HX-MS experiments [27,28]. One of the major peaks corresponded to the nucleotide binding site residues (K70, R71, L72, I73), suggesting that this NBD region could become increasingly rigid in the ATP-bound DnaK form. Other major peaks corresponded to R151, K155, R167, and I168 residues in the subdomain IA (Fig 11C and 11D). We also noticed that the high force constants were associated with functionally important residues K414 and N415 (L_2,3_ loop), 442-QGE-444 (L_4,5_ loop), and residues D481, G482 (L_6,7_ loop) (Fig 12B). Similarities between force constant and residue centrality distribution peaks suggested that a group of conserved residues in these positions may serve as local hinges that control conformational transitions and propagate allosteric changes in the residue interaction network. In particular, our results pointed to the increased centrality of the inter-domain SBD-β residues in the ATP-bound docked state. At the same time, loops L_1,2_ (residues 404–406), L_3,4_ (residues 428–434), and L_5,6_ (residues 458–473) surrounding the substrate-binding site showed distinctly lowered force constants and low centrality, indicative of the increased flexibility in this region. These results complemented the energetic analysis of protein stability and residue depth computations, unveiling the network-related consequences of the SBD-β dynamics in the substrate-binding loops. The probability distributions of residue centralities (Fig 12C and 12D) revealed a relationship indicative of a small-world network topology [95–100], that is typically linked with highly connected functional residues that mediate allosteric signaling. A significant number of high centrality sites that correspond to the distribution tail are indicative of a broad allosteric network and an ensemble of diverse communication pathways that could connect regulatory sites.

### High Centrality Nodes Define Functional Sites and Form Interacting Communities Required for DnaK Allostery

We also characterized the organization of local interaction communities that are critical for transmission of allosteric interactions and conformational changes in DnaK states. In the ADP-bound DnaK, of particular importance are communities between SBD-β and SBD-α (R445-N451-M515) and (L397-E444-M515) (S8 Fig). These communities link together the L_4,5_ loop of SBD-β (E444, R445) with the linker region (L397), and SBD-α lid (M515). Other communities may bridge the L_6,7_ loop of SBD-β (D481) with the linker (K387, D385). A tight community (K70-E171-P143) in NBD may be important for stability of the binding site and allosteric communication by coupling K70 and E171 with a proline switch residue P143 (S8 Fig). Some other local communities included (F476-V440-V486) that coupled together residues from the hydrophobic core of the SBD-β. In the ATP-bound DnaK, local communities (K70-E171-P143) and (N170-T173-D393) linked the nucleotide-binding site residues and the inter-domain linker (S9 Fig). In these communities, E171 is hydrogen-bonded to K70 and even conservative mutations of E171 reduce considerably ATP hydrolysis rates [47]. The hydrogen bonding interactions between D393 (inter-domain linker) and side-chains of N170 and T173 (subdomain IA) are coupled with a module (K70-E171-P143) that together enable efficient communication between the nucleotide site, subdomains IA and IIA, and the linker. Another stable community (N170-R151-D481) is based on N170-R151 and R151-D481 hydrogen bonding that links critical functional residues in NBD with D481 from the SBDβ subdomain (L_6,7_ loop), forming a stable inter-domain bridge. These residues may not affect the ATP hydrolysis and substrate binding but experimentally known to modulate or alter the inter-domain allosteric communication [45–51].

According to our analysis, D481 could play a special integrating role in stabilization of the NBD-SBD interface and allosteric regulation, as this residue is involved in multiple partially overlapping communities of functional residues. Indeed, another stable community (R167-I168-K155-D481) couples the subdomain IA with SBD-β subdomain through direct contacts between D481 and side-chain of R167 along the hydrogen bond between D481 and the backbone amide of I168 (S9 Fig). This stable module of high centrality sites couples D481 and I483 in the L_6, 7_ loop with highly conserved NBD residues (R151, K155 and I168). Another important line of communication is provided by the local community (Q442-D148-L454-L484) (S9 Fig) that links high centrality sites Q442 (L_4, 5_ loop) with D148 (subdomain IA, NBD), and hydrophobic residues in SBD-β: L454 (β5 strand), and L484 (β7 strand). In this community, D148 forms hydrogen bonds with Q442 and to the backbone amide of L484. Together, these residues enable the inter-domain coupling between NBD and the β4/β5/β7 hydrophobic core of SBD-β. We also detected an interacting community (K414-N415-D326-T221) that involved K414 and N415 (L_2, 3_ loop) coupled with T221 and D326 from subdomain IIA (S9 Fig). Another community (A111-Q114-L507-M515) was formed at the NBD-SBD-α interface and included high centrality resides L507, M515 (SBD-α) along with A111, Q114 (subdomain IA). The dominant contribution of the inter-domain communities to the organization of the residue interaction networks has emerged as an important factor responsible for allosteric regulation and structural changes in DnaK. Mutations of several residues in the L_2,3_ loops (K414, P419) can detrimentally affect the allosteric function DnaK [46, 47]. The importance of NBD-SBD-α contacts in allosteric coupling was also revealed in mutagenesis experiments where L507A and M515D compromised the *in vivo* activity of DnaK and showed defects in ATP-induced allosteric coupling [35]. The network-based community analysis indicated that mutations of functional residues could simultaneously weaken multiple interactions in local communities and thus severely compromise the inter-domain signaling. This approach recognized the importance of Q442 (L_4,5_ loop) and D481 (L_6,7_ loop) in organizing stable inter-domain interfaces, suggesting that network properties of these functional sites may be associated with their central role in allosteric regulation of DnaK [52]. To summarize, our results showed that functional residues are integrated into local communities that collectively form a diverse and large allosteric network. As a result, allosteric structural changes in DnaK may be mediated by stable local communities that connect the ATP binding site with the substrate-binding loops via multiple communication routes. The determined network signatures of DnaK states are characteristic of a highly cooperative allostery with a broad communication network that can tolerate random mutations without severely impairing regulatory functions.

### Different Modularity of the Residue Interaction Network in Sse1p Can Elicit an Entropy-Driven Allostery

**Fig 13:**
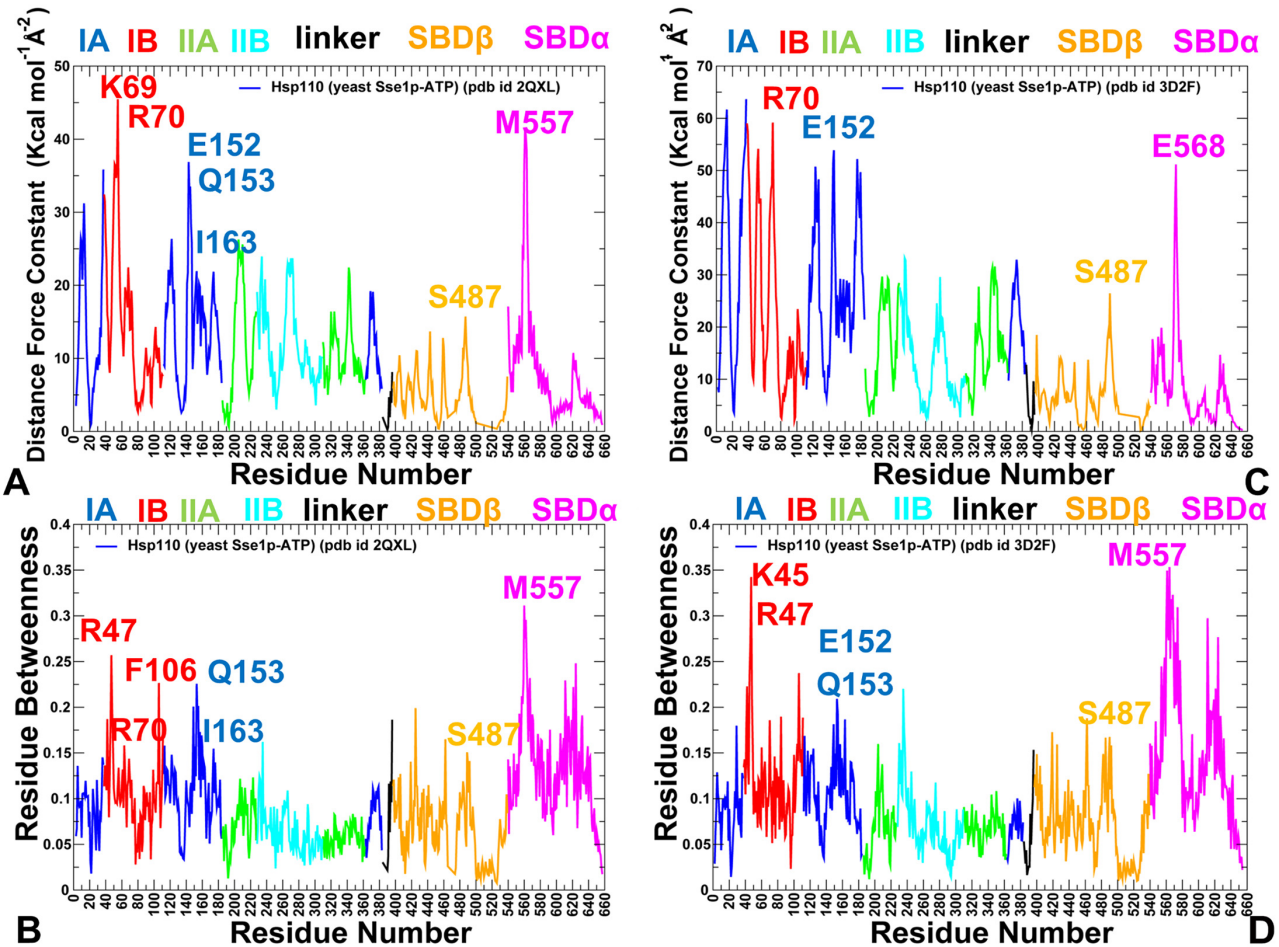
Force Constant and Network Centrality Profiles of Sse1p Structures. Residue-based force constant profiles and network centrality distributions for Sse1p-ATP (A, B), and Sse1p in a complex with the NBD of hHsp70 (C, D). The profiles are shown only for Sse1p residues. The respective profiles for the hHsp70-NBD counterpart of Sse1p in the complex are omitted for clarity of presentation. The profiles are annotated and colored according to the adopted scheme. The residue-based dynamic profiles are annotated using the residue numbering in the crystal structure of Sse1p-ATP conformation (pdb id 2QXL) and the crystal structure of Sse1p-Hsp70 complex (pdb id 3D2F). The profile peaks corresponding to functionally important residues are indicated by arrows and annotated.

**Fig 14:**
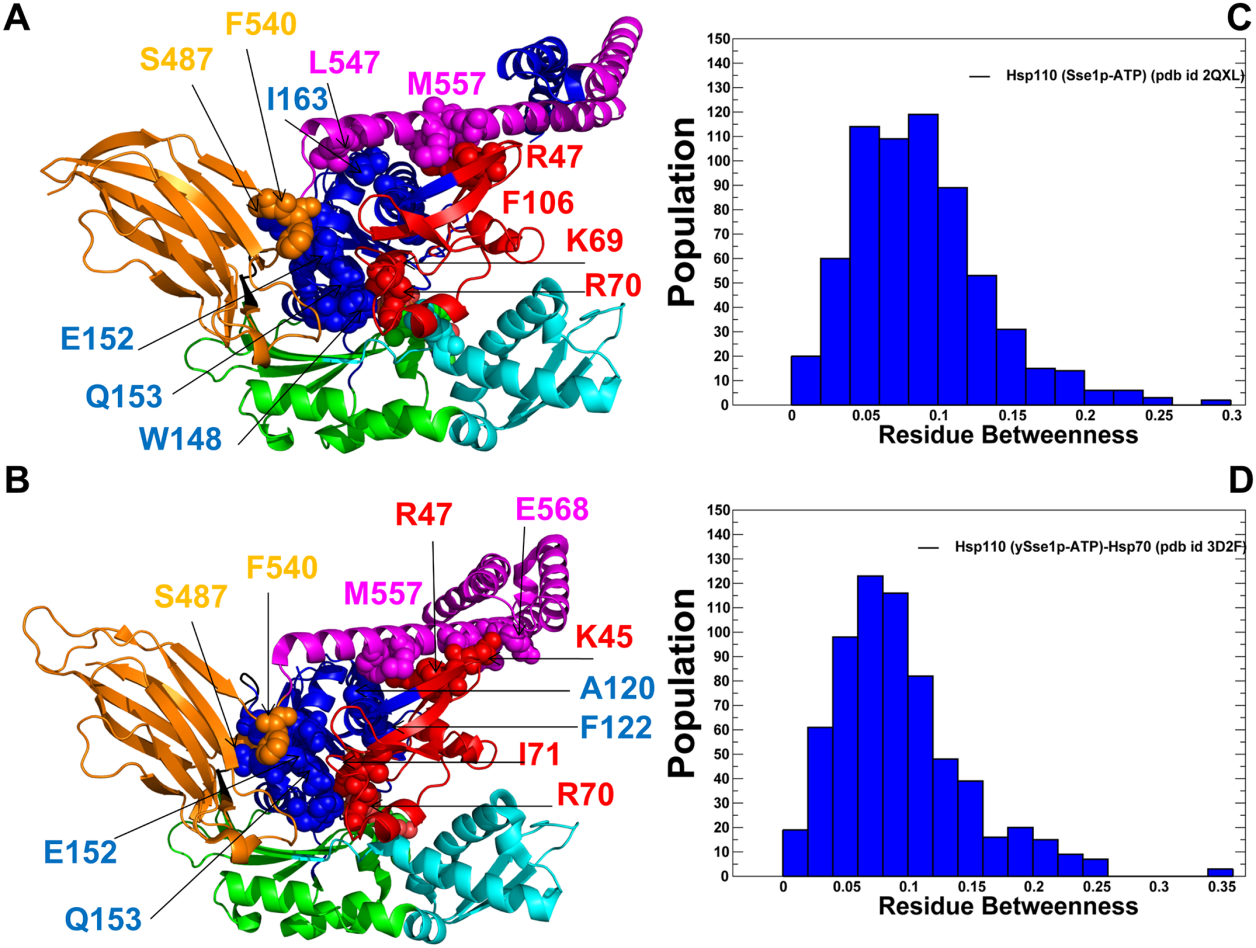
Analysis of High Centrality Residues in Sse1p Structures. Structural mapping of common peaks in the force constant and network centrality distributions onto Sse1p conformations (A, B). The structures are shown in a ribbon representation and main structural elements, including subdomains and functional residues are annotated and colored according to the adopted scheme. The functional residues of high centrality are shown in spheres and colored according to their respective subdomains. Structural positions of high centrality functional residues are indicated by arrows. The probability distributions of residue centrality in the Sse1p-ATP (C) and Sse1p in a complex with the NBD of hHsp70 (D). These profiles were obtained from MD trajectories by averaging computations of residue centrality over all protein residues in the conformational ensembles.

Using the results of MD simulations, we also constructed the force constant and residue centrality profiles for ATP-bound Sse1p (Fig 13A and 13B) and Sse1p-hHsp70 complex (Fig 13C and 13D). Despite seemingly similar distributions obtained for DnaK and Sse1p structures, a close inspection revealed subtle but important differences that may be associated with variations of allosteric mechanisms adopted by these chaperones. The central observation of this analysis was the markedly reduced residue centrality and disappearance of hinge sites in the SBD-β subdomain, which reflected the increased conformational flexibility in this region. At the same time, subdomains in the NBD appeared to be increasingly rigid, which may affect global movements in the IA-IIA cleft and rotational motions of subdomain IIB, which are required for nucleotide-dependent allostery. Interestingly, the distribution peaks migrated to residues from subdomain IA, IB and SBD-α (Fig 13). Structural mapping of high centrality residues on Sse1p conformations (Fig 14A and 14B) could illustrate a noticeable shift in the allocation of mediating sites. Among high centrality residues in Sse1p structures were K69, R70 from the nucleotide-binding site (subdomain IB); residues from subdomain IA that are important for catalytic activity (P146, E152, Q153, R154, W148,Y149); A120, F122, I163 (subdomain IA) and a group of SBD-α residues (L747, I557, M558, Q560,D561). These residues are often interconnected through hydrogen bonding and specific interactions. In particular, hydrogen bonding between catalytic residue K69 and Q153 bridges subdomains IA and IB. Some of these residues (R47, I163, and M557) are located at the NBD-SBDα interface. These results supported the notion that the integrity of the NBD-SBDα interface may be vital for Sse1p function as mutations in these positions I163D, M557S, M557D can abolish chaperone activity. [53,56].

Importantly, among multiple mediating centers of allosteric communications found in DnaK only a few critical sites sustained their high centrality in Sse1p structures. One of these residues is S487 from the L_6, 7_ loops (D481 in DnaK) that hydrogen bonded with I171 (subdomain IA). The neighboring L489 (I483 in DnaK) is located in the hydrophobic pocket formed by high centrality residues E151, R154 and Y155. Indeed, these positions are sensitive to mutations that may impair Sse1p activity [53]. The strategic role of the L_6, 7_ loop and the inter-domain interactions in this region may be common to DnaK and Sse1p chaperones. However, residue L451 from the L_4,5_ loop (Q442 in DnaK) does not make any contacts with NBD. Similarly, there are no contacts between NBD and residues corresponding to K414 and N415 from the loop L_2, 3_ (P399 and Y400 in Sse1p). Finally, the linker between SBDβ and SBDα, which contacts subdomain IB in DnaK does not interact with NBD in Sse1p. In Sse1p, the presence of stable communities formed by the NBD and SBD-α regions (F42-F106-R47-M557) and (F113-K553-M557) may be important for stabilization of the domain-docked conformation (S10 Fig). In contrast to DnaK, the allosteric interaction network in Sse1p may be relatively small because the local motions that transmit the allosteric signal are more limited. Consistent with this analysis, the probability distributions of residue centrality in Sse1p structures showed a more gradual decay and a shorter tail, with fewer high centrality mediating residues (Fig 14C and 14D). A smaller allosteric network and dislocation of mediating centers in Sse1p structures may promote an entropy-driven allostery that operates via localized changes in protein motions rather than cooperative structural changes.

**Fig 15:**
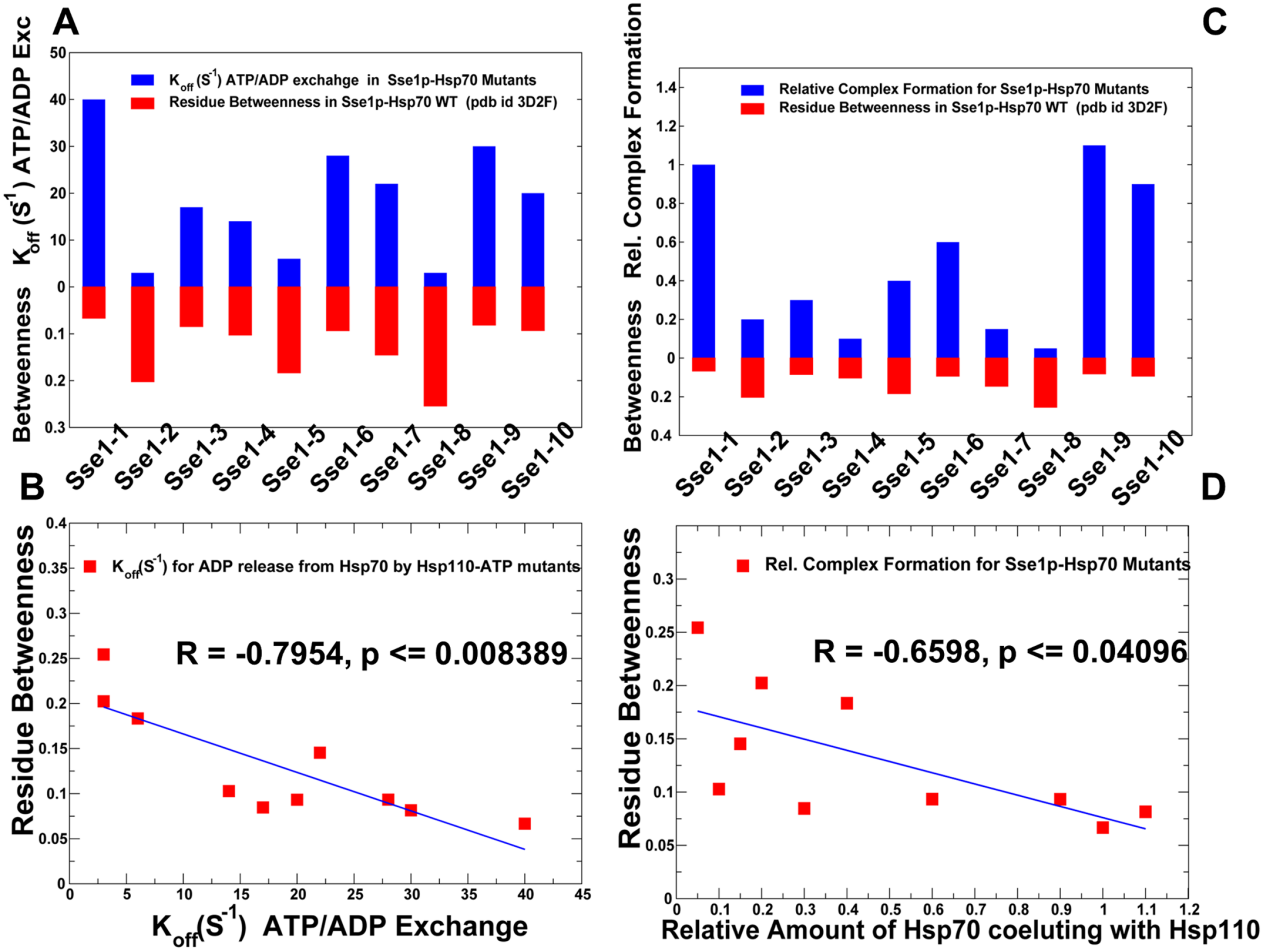
Network Analysis of Functional Effects in Sse1p Mutants. Correlations between residue centrality and different functional effects caused by clusters of mutations in Sse1p. (A, B) The relationship between residue centrality in the Sse1p complex with Hsp70 (pdb id 3D2F) and rates of the nucleotide exchange induced by Sse1p mutants. (C, D) The relationship between residue centrality in the Sse1p complex and binding affinities of Sse1p mutants measured in [53]. The clusters of mutations are annotated as in the original experimental study [53]: Sse1-1 (K69M); Sse1-2 (N572Y,E575A); Sse1-3 (A280T,N281A); Sse1-4 (T365V, N367S); Sse1-5 (F392A, F394A); Sse1-6 (D396A); Sse1-7 (L489A, H490A); Sse1-8 (E554A, M557S, L558S); Sse1-9 (L433A, N434P); Sse1-10 (F439L, M441A). To account for clusters of mutations, used in the experiments, we computed the average betweenness value over all residues in a given cluster.

To compare force constant and network-based predictions of regulatory residues with the experiments, we utilized a significant body of mutational data obtained for Sse1p-Ssa1 complex analyzed them in different functional assays [56]. These mutations were originally designed to target the nucleotide binding pocket, the inter-domain surface areas and the substrate-binding site. Instead of single substitutions, these experiments probed clusters of mutations in a given region to disrupt the interaction sites. A comprehensive analysis would require conducting independent MD simulations for all studied mutants. We elected to simplify our task and perform a direct mapping of mutational sites onto the residue centrality profile of the Sse1p-hHsp70-NBD complex (Fig 15). These network indices were correlated against various functional measurements [56]. In particular, we analyzed how network properties of functional residues may be related to the nucleotide exchange induced by Sse1p mutant proteins in Ssa1 (Fig 15A and 15B), and binding interactions of Sse1p mutants with Ssa1 (Fig 15C and 15D). Strikingly, it appeared that residue network centrality may be associated with the susceptibility of various Sse1p functions to mutations. For instance, high network centrality was found for residues in the Sse1-2 cluster (N572, E575) and Sse1-8 cluster (E554, M557, L558). Accordingly, mutations of these residues could lead to a pronounced growth defect and impaired nucleotide exchange on Hsp70, which is a central function of Sse1p [56]. A simple mapping of functional changes caused by Sse1p mutations against network properties produced a reasonable correlation and identified functionally important residue clusters. These results indicated that residue centrality may be used as a metric for differentiating functional importance of mutational effects across various chaperone functions.

Recent functional studies of Sse1 chaperones [57,58] indicated that a number of chaperone mutants could evolve new chaperone functions and employ different allosteric mechanisms, while retaining their primary function in the cell. In the context of recent structural and functional experiments [37,52], we argue that entropy-driven allostery in Sse1p could be altered or adjusted through properly engineered mutations that would reorganize the residue interaction network and activate different communication pathways that were originally suppressed. It is possible that Sse1 mutants may evolve their allosteric mechanisms by using intermediate scenarios between a less cooperative, entropy-driven allostery and a highly cooperative population-shift mechanism.

### Probing Conformational Transitions between DnaK Structures: Mechanistic Drivers of Allosteric Changes

Recent kinetic measurements of biochemical cycle in DnaK indicated that ATP binding triggers the following sequence of events: the inter-domain linker inserts first into the hydrophobic groove between subdomains IA and IIA of the NBD. This may be followed by SBD-β docking with NBD, and subsequently SBD-α binds NBD [34]. By using discrete molecular dynamics (dMD) simulations [150,151] in the framework of GOdMD approach [152,153] we probed conformational transitions between closed and open forms of DnaK to determine molecular catalysts of allosteric changes. In the basic dMD formalism particles move in the ballistic regime under constant velocity until a collision between a pair of particles occurs. In the absence of any collision, the particles move linearly with constant velocity. Residue-residue interaction potentials are defined for the particles at a distance smaller than a cut-off *r*_*c*_ in the native conformation. Otherwise, particles only interact via a hardcore repulsive potential that avoids steric clashes. In this approach, the interaction potentials include hydrogen bonding, van der Waals potentials, and a simplified solvation term. The two-step potentials that define square wells are used: a hardcore barrier preventing steric collisions at short distances and a single potential step at the interaction cutoff distance *r*_*c*_ [151]. According to this implementation of the dMD approach [152,153], secondary structure elements were reinforced by using finite square wells between hydrogen-bonded residues with a depth of 10 kcal/mol, centered at the C_α_-C_α_ distance in the native conformation and with a width corresponding to 10% of this distance. We assumed that conformational mobility in the SBD-β subdomain may provide an important factor that could facilitate structural transitions, thus providing thermodynamic and kinetic means for allosteric communication in DnaK. The computed pathways followed pattern of easiest protein deformations from the reference structure and should be considered as a hypothesis generator rather than a rigorous computational experiment.

In these simulations, we monitored various structural and network parameters: root mean square deviation (RMSD) between the initial structure (closed ADP-bound DnaK conformation) and targeted structure (ATP-bound DnaK) the evolution of residue hubs, cliques, and communities (S11 Fig). Despite a relatively simple model, simulations reproduced a first order-like transition between DnaK states with a characteristic sigmoidal curve. These observations reflect a highly cooperative nature of allosteric structural changes in DnaK, which is consistent with the structural and functional studies. We characterized a range of intermediate structures near the hypothetical transition state region to describe major topological features of these conformations and identify key interactions. We first inspected more closely structures that appeared immediately before the “transition state” as judged by the RMSD curve (S6 Fig). A distinct feature of these transient conformations is that the inter-domain linker may be “pushed” towards NBD by the L_2,3_ loop residues. In these structures, functionally important N415 (L_2,3_ loop) makes contacts with the linker residue D393, while D393 appeared to approach K155 from subdomain IA of NBD. This transient arrangement may navigate the inter-domain linker closer to NBD, forcing linker to slide along subdomain IA towards the hydrophobic groove. Although these structures and interactions may be transient in nature and could be highly hypothetical, it is interesting to notice that key regulatory residues seem to be involved in thermodynamic and kinetic aspects of allosteric changes. Noteworthy, these conformations depicted that the linker docking may represent the first step of the reaction. Also, the observed structural changes required a considerable remodeling of the SBD-β subdomain, suggesting that conformational flexibility in this region may be a prerequisite for proper progression along the reaction coordinate. By analyzing conformational states that corresponded to “post-transition” ensemble (S7 Fig), we observed that linker binding may be followed by simultaneous docking of the SBD-β and SBD-α subdomain. In this conformation, the SBD-α docked position is native but not fully optimized with some inter-domain interactions out of register. A complete remodeling and repositioning of the SBD-β loops into targeted structure occurred only at the later stages of the reaction (S12 Fig). At this point, L_6,7_ and L_2,3_ loops switched their positions with respect to NBD, so D481 (L_6,7_ loop) may regain its integrating role in stabilizing the NBD-SBD interface. According to kinetic experiments [34], three major events of the reaction followed double-exponential kinetics with a fast phase rate of 107 s^-1^ (linker binding), 27.9 s^-1^ (SBD-β docking with NBD) and 8.3 s^-1^ (SBD-α lid docking). In other words, linker binding clearly precedes subsequent docking of the SBD subdomains that may occur more cooperatively. Despite considerable simplification of the conformational transitions in our modeling, we correctly predicted the early formation of a linker-docked intermediate, while simultaneous docking of the SBD-β and SBD-α may reflect a highly cooperative nature of allosteric changes in DnaK. Obviously, the observed pre-transition and post-transition conformations along the simulated reaction pathway are highly hypothetical and may only approximately reflect topological rearrangements that occur during allosteric transitions. However, our results demonstrated that kinetics of allosteric changes may require significant structural remodeling of the SBDβ subdomain. As a result, conformational flexibility of the SBDβ may be a necessary thermodynamic and kinetic factor driving propagation of allosteric structural changes in Dnak. Mechanistic insights obtained from this simplified model offered an interesting and plausible rationale, suggesting that key functional residues and structural segments may be involved in both thermodynamic and kinetic aspects of allosteric regulation in DnaK.

## Conclusions

By combining molecular simulations, protein stability analysis and network-based modeling, the reported study provided a comprehensive computational analysis of allosteric mechanisms in DnaK and Sse1p chaperones. We showed that allosteric regulation of these chaperones may be determined by nucleotide-induced redistribution of local conformational ensembles in the inter-domain regions and the substrate binding domain. Our results also revealed how organization of the residue interaction networks in stable local communities of functional residues are linked with the allosteric mechanisms. We found that global mediating residues with high network centrality may be organized in stable local communities that are involved in the efficient allosteric communications. The results of this study suggested that allosteric structural changes in DnaK may be highly cooperative and conducive to a population-shift allosteric mechanism. In this mechanism, a number of high centrality DnaK residues form a broad allosteric network that can link the nucleotide-binding and the substrate-binding regions. Conformational dynamics and energetics of the peptide substrate binding with the DnaK structures, that was examined using free energy calculations, identified allosteric hotspots of negative cooperativity between regulatory binding sites. A highly cooperative allosteric mechanism in DnaK could ensure a proper balance of the network efficiency and robustness required to maintain resilience against random mutations and structural perturbations without compromising critical functions. In contrast, the allosteric interaction network in Sse1p may be smaller, leading to an entropy-driven allostery that occurs in the absence of structural changes. These results indicated that organization of the residue interaction networks in DnaK and Sse1p may be among determining factors of their allosteric mechanisms that have evolved to achieve a trade-off between structural stability, the efficiency of allosteric communications and resilience against mutations. We also demonstrated that network centrality of functional residues could be linked with their sensitivity to mutations and may explain functional importance of mutational effects across a diverse spectrum of chaperone functions. The association of network properties with chaperone regulation suggested that residue interaction networks may be specifically tailored through protein engineering or therapeutic intervention that is informed by knowledge of high centrality network nodes. Integration of genetic, biochemical and structural data in the network-centric framework may help to understand the complex relationships between robustness of chaperones and their functional specificity. These system-based approaches can exploit advances in biology and network science to develop novel symbiotic strategies for understanding complex protein systems and their interconnectivity in biological networks.

## Materials and Methods

### MD Simulations

MD simulations of the DnaK and Sse1p structures (500 ns for each structure) were performed for an ADP-bound DnaK, pdb id 2KHO [31], the crystal structure of an ATP-bound DnaK, pdb id 4B9Q [34]; the crystal structure of a Sse1p-ATP [53], and the crystal structure of Sse1p in a complex with the NBD of hHsp70 [56]. All crystal structures were obtained from the Protein Data Bank (RCSB PDB www.rcsb.org) [154]. All crystallographic water molecules were removed and missing hydrogen atoms of the protein were added. All ionizable residues were considered in the standard ionization state at neutral pH condition. The missing residues, unresolved structural segments and disordered loops were modeled and evaluated with the ModLoop server [155,156] and the ArchPRED server [157]. The unresolved portions were assembled and energetically refined using the ArchPRED server. MD simulations were carried out using NAMD 2.6 package [158] with the CHARMM27 force field [159] and the explicit TIP3P water model. The employed MD protocol is consistent with the overall setup that was described in details in our earlier studies [160–162]. The following protocol preceded the production stage of MD simulations. All atoms of the complex were first restrained at their crystal structure positions with a force constant of 10 Kcal mol^-1^ Å^-2^. The system was subjected to the following simulation annealing to ensure the proper equilibration. The temperature was increased from 0K to 500K at a rate of 1K per 1ps and was kept at 500K for 500ps. The temperature was then decreased from 500 K to 300K at a rate of 1K per 1ps and was kept at 300K for additional 500ps. An NPT production simulation was then run on each of the equilibrated structures keeping the temperature at 300 K and constant pressure of 1 atm. PCA of the MD conformational ensembles was based on the set of backbone heavy atoms (N, Cα, Cβ, C, O) and on the Cα atoms only to determine the essential dynamics of the protein systems. The calculations were performed using the CARMA package [163]. The frames are saved every 5 ps, and a total of 10,000 frames were used to compute the correlation matrices for each simulation. For comparison, we also employed the elastic network model (ENM) and computed ENM-based lowest normal modes using the Anisotropic Network Model web server [164].

### Force Constant Analysis of Residue Deformations

Mechanical properties of the DnaK and Sse1p structures are analyzed using the force constant approach [101,102]. In this model, the fluctuations of the mean distance between a given residue and all other residues in the protein structure are evaluated by computing the force constant profile that measures the energy cost of displacing a given residue during MD simulations. We computed the fluctuations of the mean distance between each atom within a given residue and the atoms that belong to the remaining residues of the protein. The force constant for each residue is computed as the average of the force constants for all its atoms. Alternatively, the mean fluctuations of a given residue can be also characterized using only *C*_*α*_ atom positions. In our model, the force constant for each residue is calculated by averaging the distances between the residues over the MD trajectory using the following expression:

$$k_{i}=\frac{3k_{B}T}{\langle {\left(d_{i}-\langle d_{i}\rangle \right)}^{2}\rangle }$$ (1)

$$d_{i}={\langle d_{ij}\rangle }_{j*}$$ (2)

where *d*_*ij*_ is the instantaneous distance between residue *i* and residue *j*, *k*_*B*_ is the Boltzmann constant, *T* = 300K. 〈〉 denotes an average taken over the MD simulation trajectory and *d*_*i*_ = 〈*d*_*ij*_〉_j*_ is the average distance from residue *i* to all other atoms *j* in the protein (the sum over j* implies the exclusion of the atoms that belong to the residue *i*). The interactions between the *C*_*α*_ atom of residue *i* and the *C*_*α*_ atom of the neighboring residues *i*-1 and *i*+1 are excluded in the calculation since the corresponding distances are nearly constant. The inverse of these fluctuations yields an effective force constant *k*_*i*_ that describes the ease of moving an atom with respect to the protein structure. The residue-based force constant profiles are used to characterize structural stability and conformational flexibility of protein residues.

### Protein Structure Network Analysis

In the protein structure network analysis, a graph-based representation of proteins was used in which amino acid residues were considered as nodes connected by edges corresponding to the nonbonding residue-residue interactions. The details of the construction of such a graph at a particular interaction cut-off (*I*_min_) were extensively discussed [93,94]. Here, we describe the main steps in the construction of protein structure networks pertinent to the present study. The interactions between side chain atoms of amino acid residues (nodes) define edges of the protein structure network and are evaluated from the normalized number of contacts between nodes. The non-covalent interactions between sequence neighbors are ignored in the graph construction. The interaction between two residues *i* and *j* is measured as

$$I_{ij}=\frac{n_{ij}}{\sqrt{\left(N_{i}\times N_{j}\right)}}\times 100$$ (3)

where *n*_*ij*_ is number of distinct atom pairs between the side chains of amino acid residues *i* and *j* that lie within a distance of 4.5 Å. *N*_*i*_ and *N*_*j*_ are the normalization factors for residues *i* and *j* respectively. We have determined the normalization factors *N*_*i*_ for all 20 residue types as was described in previous studies [93]. The number of interaction pairs including main-chain and side-chain made by residue type *i* with all its surrounding residues is also evaluated. The normalization factors take into account the differences in the sizes of the side chains of the different residue types. The pair of residues with the interaction *I*_*ij*_ greater than a cut-off (*I*_min_) are connected by edges and produce a protein structure network graph for a given interaction cutoff *I*_min_. According to the analysis of a large number of protein structures, *I*_min_ values could vary from 1% to 15%, where the lower *I*_min_, the higher is the graph connectivity. The optimal interaction cutoff was determined as the transition point for the largest connected cluster. According to this definition, the *I*_min_ value often lies in the range 2–4% for a diverse spectrum of protein systems [93,94]. A similar analysis was conducted in our study. In the graph-based analysis performed in the present study, at *I*_min_ = 1%, all residue nodes are connected by edges, while at *I*_min_ = 10%, there are typically very few residue nodes connected by non-covalent edges. We found that the appropriate transition value for the cut-off *I*_min_ = 2.0%-2.5%. Hence, in the present study, any pair of residues are connected in the protein structure graph if *I*_min_ = 2.5%.

### Network Centrality and Community Analysis

A weighted network representation of the protein structure is adopted in this study that includes non-covalent connectivity of side chains and residue cross-correlation fluctuation matrix [165]. In this model of a protein network, the weight *w*_*ij*_ of an edge between nodes *i* and *j* is determined by the dynamic information flow through that edge as measured by the correlation between respective residues. The weight *w*_*ij*_ is defined as w_*ij*_ = −log(|*C*_*ij*_|) where *C*_*ij*_ is the element of the covariance matrix measuring the cross-correlation between fluctuations of residues is *i* and *j* obtained from MD simulations. The shortest paths between two residues are determined using the Floyd–Warshall algorithm [166] that compares all possible paths through the graph between each pair of residue nodes. To select the shortest paths that consist of dynamically correlated intermediate residues, we considered the short paths that included correlated (*C*_*ij*_ = 0.5–1.0) intermediate residues. Using the constructed protein structure networks, we computed the residue-based betweenness parameter. The betweenness of residue *i* is defined to be the sum of the fraction of shortest paths between all pairs of residues that pass through residue *i*:

$$C_{b}(n_{i})=\overset{N}{\underset{j<k}{\sum }}\frac{g_{jk}(i)}{g_{jk}}$$ (4)

Where *g*_*jk*_ denotes the number of shortest geodesics paths connecting *j* and *k*, and *g*_*jk*_(*i*) is the number of shortest paths between residues *j* and *k* passing through the node *n*_*i*_. Residues with high occurrence in the shortest paths connecting all residue pairs have a higher betweenness values. For each node *n*, the betweenness value is normalized by the number of node pairs excluding *n* given as (*N*-1)(*N*-2)/2, where *N* is the total number of nodes in the connected component that node *n* belongs to. The normalized betweenness of residue *i* can be expressed as follows:

$$C_{b}(n_{i})=\frac{1}{\left(N-1)(N-2\right)}\overset{N}{\underset{\begin{array}{l} j<k\\ j\neq i\neq k \end{array}}{\sum }}\frac{g_{jk}(i)}{g_{jk}}$$ (5)

*g*_*jk*_ is the number of shortest paths between residues *j* and k; *g*_*jk*_(*i*) is the fraction of these shortest paths that pass through residue *i*.

The analysis of the interaction networks was done using network parameters such as hubs, cliques and communities. The hubs are highly connected nodes in the network. If the total number of edges incident on the node (called the degree of a node) is at least 4 the node is identified as a hub. The *k*-cliques are complete sub graphs of size *k* in which each node is connected to every other node. In our application, a *k*-clique is defined as a set of *k* nodes that are represented by the protein residues in which each node is connected to all the other nodes. A *k*-clique community is determined by the Clique Percolation Method [167] as a subgraph containing *k*-cliques that can be reached from each other through a series of adjacent *k*-cliques. We have used a community definition according to which in a *k*-clique community two *k*-cliques share *k*−1 or *k*−2 nodes. Computation of the network parameters was performed using the Clique Percolation Method as implemented in the CFinder program [168]. Given the chosen interaction cutoff *I*_min_ we typically obtain communities formed as a union of *k* = 3 and *k* = 4 cliques. The interaction cliques and communities were considered to be dynamically stable if these interaction networks remained to be intact in more than 75% of the ensemble conformations. The conformational ensemble used in the protein network analysis was obtained from MD simulations and included a total of 1,000 representative snapshots.

### Binding Free Energy Calculations

The binding free energy was calculated using MM-GBSA approach [135,136]. In this approach the binding free energy Δ*G*_*bind*_ is written as the sum of the gas phase contribution Δ*G*_*MM*_, the solvation free energy Δ*G*_*solv*_, and an entropic contribution–*T*Δ*S*

$$\Delta G_{bind}=\langle \Delta G_{MM}\rangle +\langle \Delta G_{solv}\rangle -\langle T\Delta S\rangle$$ (6)

The brackets <> denote an average of these contributions calculated over the MD trajectories. The gas-phase contribution <Δ*G*_*MM*_> to the binding free energy is the difference in the molecular mechanics energy of the complex and the isolated protein and ligand. This contribution is the sum of the differences in the internal energies Δ*E*_intra_, the van der Waals interaction energy Δ*E*_*vdw*_, and the electrostatic interaction energy Δ*E*_*elec*_:

$$\langle \Delta G_{MM}\rangle =\Delta E_{\text{intra}}+\Delta E_{vdw}+\Delta E_{elec}$$ (7)

$$E_{\text{intra}}=E_{bond}+E_{vdw}+E_{elec}$$ (8)

where *E*_*bond*_ is the energy of the bonded terms (bonds, angles, dihedral angles, and improper angles) of a given molecule; *E*_*vdw*_ is the van der Waals energy of the molecule; and *E*_*elec*_ is the electrostatic energy of the molecule. These contributions are calculated according to the molecular mechanics force field. The solvation free energy Δ*G*_*solv*_ is the difference between the solvation energy of the complex and solvation free energies of the isolated protein and ligand:

$$\Delta G_{solv}=\Delta G_{solv}^{complex}-\Delta G_{solv}^{protein}-\Delta G_{solv}^{ligand}$$ (9)

$$\Delta G_{solv}=\Delta G_{solv}^{np}+\Delta G_{solv}^{elec}$$ (10)

The solvation free energy of a molecule is given as the sum of nonpolar and polar contributions. The nonpolar contribution is computed using the solvent accessible surface are (SASA) model and give as $\Delta G_{solv}^{np}=\sigma *SASA$ where the parameter σ = 0.0072 kcal/ (mol*Ǻ^2^). The electrostatic contribution to the solvation free energy $\Delta G_{solv}^{elec}$ was calculated using the analytical generalized Born (GB) model implemented in CHARMM. This model is known to accurately reproduce the solvation free energies calculated by solving the Poisson equations. All energy terms were calculated for 10,000 frames regularly separated by 50 ps along the 500ns trajectories.

The entropy contribution consists of translational Δ*S*_*trans*_, rotational Δ*S*_*rot*_ and vibrational Δ*S*_*vib*_ components:

$$\Delta S=\Delta S_{trans}+\Delta S_{rot}+\Delta S_{vib}$$ (11)

The vibrational entropy terms were computed using normal mode analysis that yields better convergence than the quasiharmomic analysis from MD trajectories. The VIBRAN module was used to calculate and diagonalize the force constant matrix for the normal mode vectors and frequencies determination. The normal modes were calculated on minimized average structures obtained from MD simulations. The minimization was performed using the Newton–Raphson minimization algorithm, using the same cutoff scheme and constraints as for the normal mode calculations. All energy terms are calculated using single 500 ns trajectories of the peptide-DnaK complexes. This is followed by separation of the complexes into isolated protein and ligand structures and subsequent minimization of the isolated molecules without conducting additional simulations of the individual protein.

Computational alanine scanning was performed by replacing the side chain of a given residue by an alanine and recalculating the absolute binding free energy for the mutated system. The difference in the binding free energy of the wild type and alanine mutant ΔΔ*G*_*bind*_ may be evaluated as follows:

$$\Delta \Delta G_{bind}=\Delta G_{bind}^{mut}-\Delta G_{bind}^{WT}$$ (12)

$$\langle \Delta \Delta G_{bind}\rangle =\langle \Delta E_{vdw}\rangle +\langle \Delta E_{elec}\rangle +\langle \Delta \Delta G_{solv}^{np}\rangle +\langle \Delta \Delta G_{solv}^{elec}\rangle -T\langle \Delta \Delta S\rangle$$ (13)

The binding free energy of the alanine mutant is calculated using the MM-GBSA approach using the snapshots obtained from MD simulations of the WT complex. All energy terms were calculated for 10,000 snapshots along the 500ns trajectory performed for the WT complex. For each of these snapshots of the WT complex, the mutated side chain was minimized under the fixed position of the remaining system using 1000 steps of steepest decent and Newton–Raphson minimization before calculating the energy terms. A central assumption of computational alanine scanning approach is that mutations would introduce only local structural perturbations of the system that are sufficiently moderate that the effect on the binding free energy may be gleaned from MD simulations of the WT system.

## Supporting Information

S1 Fig Conformational Ensembles of Closed and Open DnaK Forms. Structurally different representative conformations extracted by clustering MD trajectories of an ADP-bound, closed DnaK form (A, B) and ATP-bound, open DnaK form (C, D). Different view angles are adopted for convenience of visualization to illustrate ADP-DnaK conformations (A, B) and ATP-DnaK conformations (C, D). The structures are shown in a ribbon representation and main structural elements are annotated as in Fig 2. The NBD subdomains are colored according to the adopted scheme: IA (in blue), IB (in red), IIA (in green), IIB (in cyan), the inter-domain linker (in black), SBD-α (in magenta), and SBD-β (in orange). Shear movements of the SBD-α around SBD-β and rotations of the subdomain IIB are observed.
(TIF)

S2 Fig Structural Annotation of the SBD Binding Loops in the Closed and Open DnaK Forms. A close-up view and annotation of the SBD-β loops in the ADP-bound (A) and ATP-bound DnaK conformations. The SBD-β loops are annotated and shown in orange ribbons. In (A), the elements of the SBD-α lid are also shown and annotated (in magenta). L_2,3_ loop (residues 412–420), L_4,5_ loop (residues 439–457) and L_6,7_ loop (residues 479–482) are involved in the inter-domain interactions. L_1,2_ loop (residues 404–406), L_3,4_ loop (residues 428–434), and L_5,6_ loop (residues 458–473) are located near the substrate-binding site.
(TIF)

S3 Fig The Density of States for the ADP-DnaK Conformational Ensemble. The density of states for the ADP-DnaK was derived from the equilibrium conformational ensemble. (A) The density distribution as a function of the force field energy and the rigid body RMSD for the SBD-α subdomain from its native position in the ADP-DnaK structure (pdb id 2KHO). (B) The domain-undocked crystallographic conformation corresponds to the dominant peak in the density distribution. (C) A representative partly undocked conformation, in which the SBD-α lid deviates from the SBD-β–SBD-α interface, corresponds to a shallow intermediate peak in the density distribution. The structures are shown in a surface representation and main structural elements are annotated. The NBD subdomains are colored as follows: IA (in blue), IB (in red), IIA (in green), IIB (in cyan), the inter-domain linker (in black), SBD-α (in magenta), and SBD-β (in orange).
(TIF)

S4 Fig The Density of States for the ATP-DnaK Conformational Ensemble. The density of states for the ATP-DnaK ensemble. (A) The density distribution as a function of the force field energy and the rigid body RMSD for the SBD-α subdomain from its native position in the ATP-DnaK structure (B) The domain-docked crystallographic conformation corresponds to the dominant peak in the density distribution. (C) A representative undocked conformation corresponds to the secondary peaks in the distribution. The structures are shown in a surface representation and main structural elements are annotated. The NBD subdomains are colored as in S3 Fig.
(TIF)

S5 Fig Residue Deformability Profiles of DnaK and Sse1p Structures. The residue-based deformability profiles of the solution structure of an ADP-bound DnaK, pdb id 2KHO (A); the crystal structure of an ATP-bound DnaK, pdb id 4B9Q (B); the crystal structure of a Sse1p-ATP (C); and the crystal structure of Sse1p in a complex with the NBD of hHsp70 (D). In (D) deformability profile is shown only for Sse1p residues. The deformability profiles are annotated and colored according to the adopted coloring scheme of the chaperone subdomains: IA (in blue), IB (in red), IIA (in green), IIB (in cyan), the inter-domain linker (in black), SBD-α (in magenta), and SBD-β (in orange).
(TIF)

S6 Fig Structural Mapping of the SBD-β Hydrophobic Core in DnaK States. The hydrophobic core residues (V440, L454, L484) are mapped along with the F426 and I438 (substrate binding site hinge points), Q442 and D148(NBD) residues in the ADP-DnaK (A, pdb id 2KHO) and the ATP-DnaK structures (B, pdb id 4B9Q), (C, pdb id 4JNE). The SBD-β domain is shown in orange ribbons and the substrate binding loops are annotated: L_1,2_ (residues 404–406), L_3,4_ (residues 428–434), and L_5,6_ (residues 458–473). In the ADP-DnaK form (A) the SBD-α lid is also shown (in magenta ribbons). Residues are shown in blue spheres and annotated. Note the formation of the interacting clusters in the ATP-DnaK structures that link the substrate binding site (F426 and I438) with the hydrophobic core (V440, L454, L484) and the inter-domain interface (Q442, D148).
(TIF)

S7 Fig Structural Mapping of Residues with Low Solvent Protection in the SBD-β Domain of Sse1p. The SBD-β residues with the small RD values (and low protection level) are shown in red spheres. The SBD-β domain is shown in orange ribbons and the substrate binding loops are annotated: L_1,2_ (residues 407–413), L_3,4_ (residues 436–438), and L_5,6_ (residues 466–475). The locations of the putative substrate recognition site are indicated by arrow.
(TIF)

S8 Fig Structural Mapping of Local Interaction Communities in the ADP-bound DnaK State. Structural mapping of principal local communities in the ADP-bound DnaK. A close-up of interactions in local communities formed between SBD-β and SBD-α residue (R445-N451-M515) (A) and (L397-E444-M515) (B). A community (D481-K387-L385) of residues from the SBD-β and the linker regions (C). A community (K70-E171-P143) connects regulatory residues in NBD. The interacting residues are shown in colored sticks, the DnaK domains are shown in ribbons with a reduced transparency. Annotation and coloring are according to the adopted scheme: IA (in blue), IB (in red), IIA (in green), IIB (in cyan), the inter-domain linker (in black), SBD-α (in magenta), and SBD-β (in orange). The residue numbering is in accordance with the solution structure of an ADP-bound DnaK, pdb id 2KHO.
(TIF)

S9 Fig Structural Mapping of Local Interaction Communities in the ATP-bound DnaK State. Structural mapping of principal local communities in the ATP-bound DnaK. A close-up view of interactions in a local community (R167-I168-K155-D481) that couples the subdomain IA with the SBD-β (A). An overview of interactions in a local community (Q442-D148-L454-L484) that links L_4,5_ loop in the SBD-β, hydrophobic core of the SBD-β and subdomain IA of NBD (B). A community (K414-N415-D326-T221) couples residues from L_2,3_ loop with the subdomain IIA (C). A close-up of interactions in a community (A111-Q114-L507-M515) formed at the NBD-SBD-α interface (D). The interacting residues are shown in colored sticks, the DnaK domains are shown in ribbons with a reduced transparency. Annotation and coloring are according to the adopted scheme. The residue numbering is in accordance with the crystal structure of an ATP-bound DnaK, pdb id 4B9Q.
(TIF)

S10 Fig Structural Mapping Local Interaction Communities in the Sse1p-ATP Structures. Structural mapping of l local communities in the Sse1p-ATP. A close-up view of interactions in the inter-domain communities involving NBD and SBD-α residues (F42-F106-R47-M557) (A) and (F113-K553-M557) (B). The interacting residues are shown in colored sticks. Sse1p domains are shown in ribbons with a reduced transparency. Annotation and coloring are according to the adopted scheme The residue numbering is in accordance with the crystal structure of an Sse1p-ATP, pdb id 2QXL.
(TIF)

S11 Fig Structural and Network Parameters of Conformational Transitions between DnaK Structures. Structural and network parameters of conformational changes between the initial (closed ADP-bound DnaK conformation) and targeted structure (open ATP-bound DnaK conformation). (A) The RMSD between the initial and targeted structures as a function of reduced simulation time units (R.T.U). The RTU parameter is defined according to [152,153] as a ratio of the number of total collisions (scaled by 0.15) to the number of residues in the system at T = 300K. In this model, RTU typically corresponds to 20–50 ps time in conventional equilibrium dynamics. (B-D) Evolution of the network parameters during conformational transitions: number of hubs (B), cliques (C), and communities (D).
(TIF)

S12 Fig A Schematic Overview of the Conformational Transition Reaction in DnaK. A schematic overview of the conformational transition between the initial (closed ADP-bound DnaK conformation). The initial structure (the solution structure of an ADP-bound DnaK, pdb id 2KHO) and the targeted structure (the crystal structure of an ATP-bound DnaK, pdb id 4B9Q) are shown in ribbons and subdomains are colored as follows: NBD (in blue), SBD (in red), and the inter-domain linker (in yellow). The high centrality inter-domain residues are shown in spheres. The depicted pre-transition state and post-transitional state are representative structures from ensembles of conformations sampled immediately prior and after a major transition. The transition region is approximately defined from the first-order sigmoidal curve. The residues that form the inter-domain contacts in these states are shown in spheres and colored according to their subdomains.
(TIF)
