# Supplementary figures and images for: Dancing through Life: Molecular Dynamics Simulations and Network-Centric Modeling of Allosteric Mechanisms in Hsp70 and Hsp110 Chaperone Proteins
Source: PLoS One. 2015 Nov 30;10(11):e0143752. doi: 10.1371/journal.pone.0143752 (PMC4664246; doi:10.1371/journal.pone.0143752)

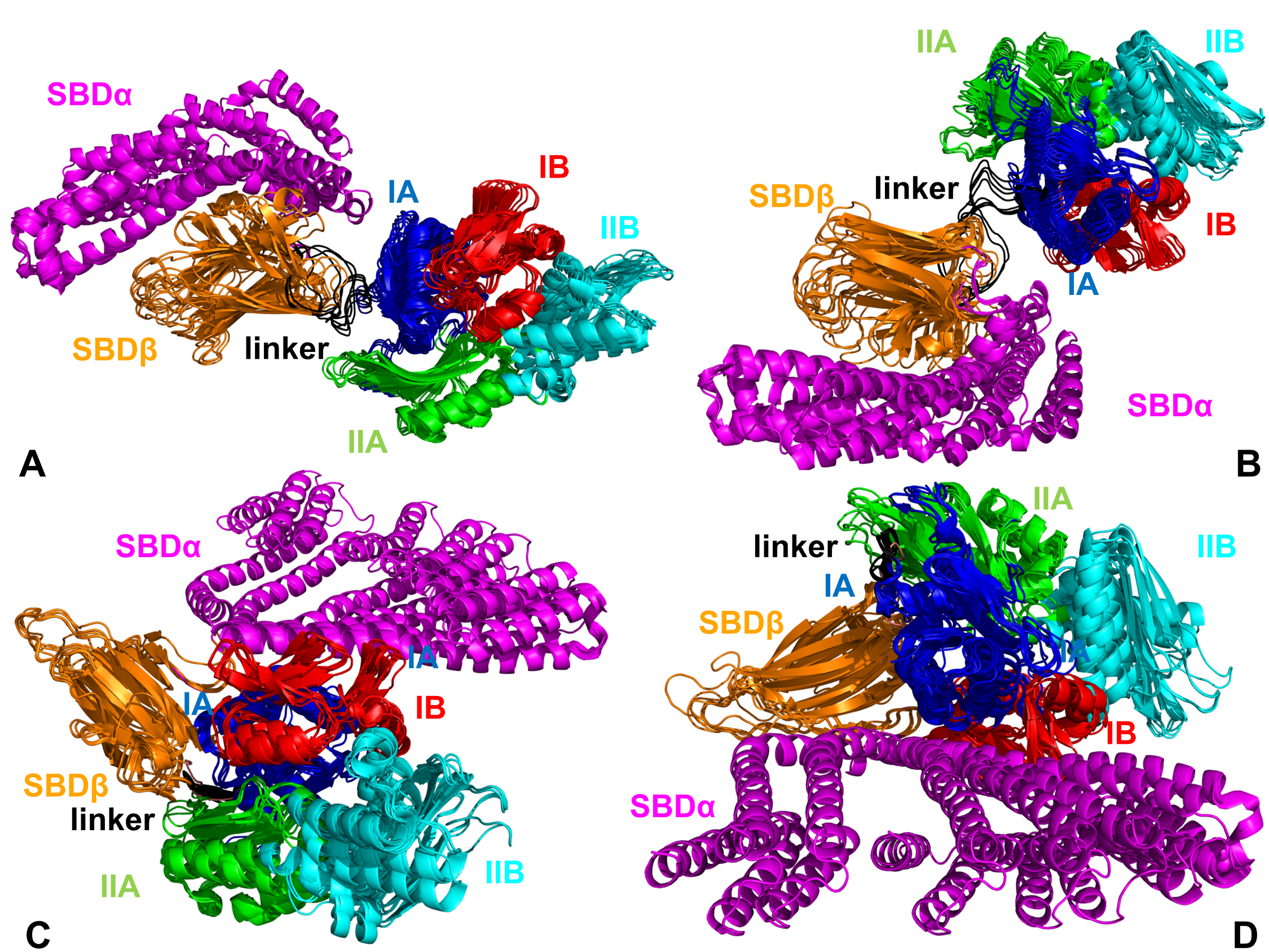

Supplement: S1 Fig — Structurally different representative conformations extracted by clustering MD trajectories of an ADP-bound, closed DnaK form (A, B) and ATP-bound, open DnaK form (C, D). Different view angles are adopted for convenience of visualization to illustrate ADP-DnaK conformations (A, B) and ATP-DnaK conformations (C, D). The structures are shown in a ribbon representation and main structural elements are annotated as in Fig 2. The NBD subdomains are colored according to the adopted scheme: IA (in blue), IB (in red), IIA (in green), IIB (in cyan), the inter-domain linker (in black), SBD-α (in magenta), and SBD-β (in orange). Shear movements of the SBD-α around SBD-β and rotations of the subdomain IIB are observed. (TIF) [file pone.0143752.s001.tif]

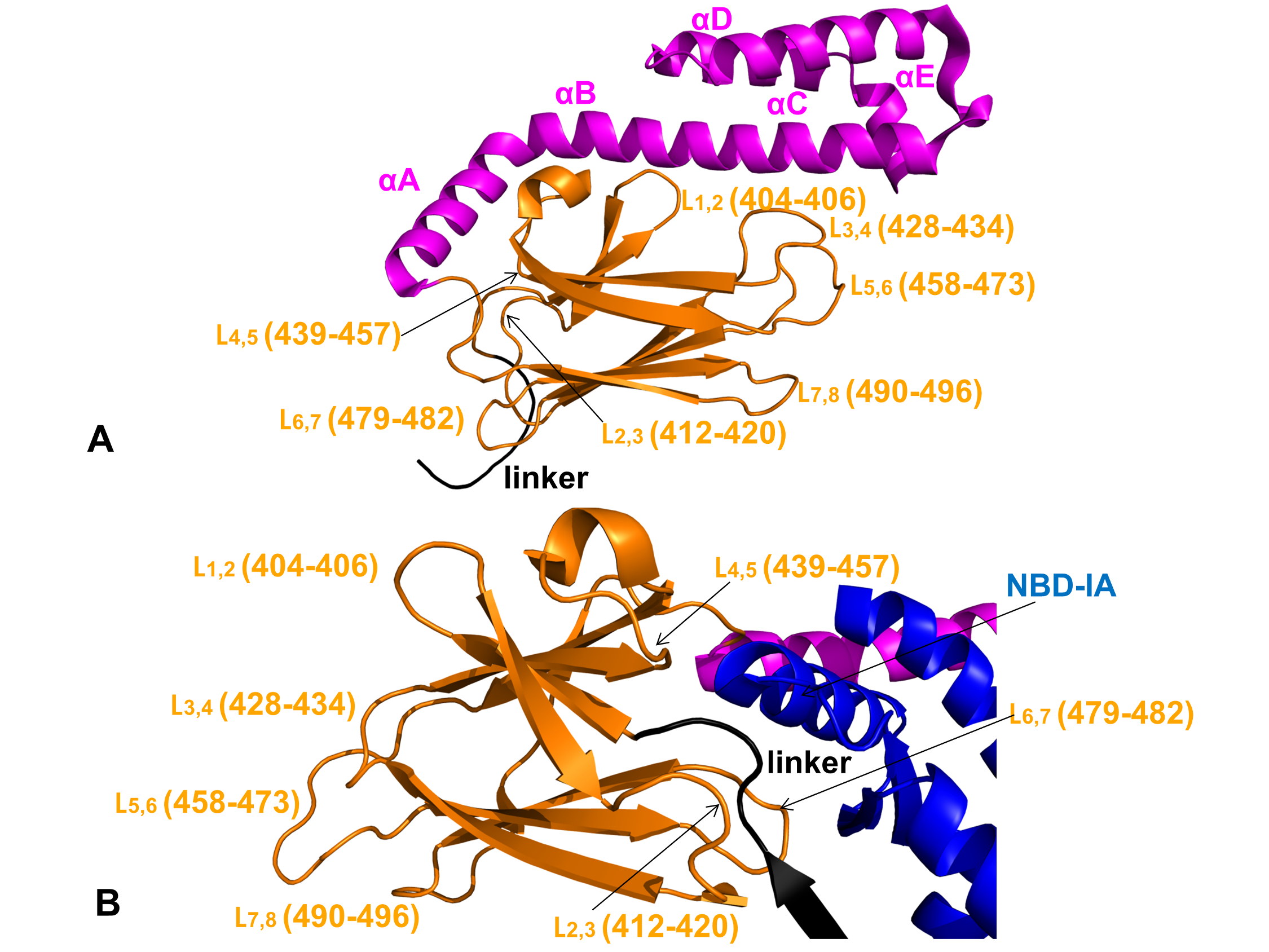

Supplement: S2 Fig — A close-up view and annotation of the SBD-β loops in the ADP-bound (A) and ATP-bound DnaK conformations. The SBD-β loops are annotated and shown in orange ribbons. In (A), the elements of the SBD-α lid are also shown and annotated (in magenta). L2,3 loop (residues 412–420), L4,5 loop (residues 439–457) and L6,7 loop (residues 479–482) are involved in the inter-domain interactions. L1,2 loop (residues 404–406), L3,4 loop (residues 428–434), and L5,6 loop (residues 458–473) are located near the substrate-binding site. (TIF) [file pone.0143752.s002.tif]

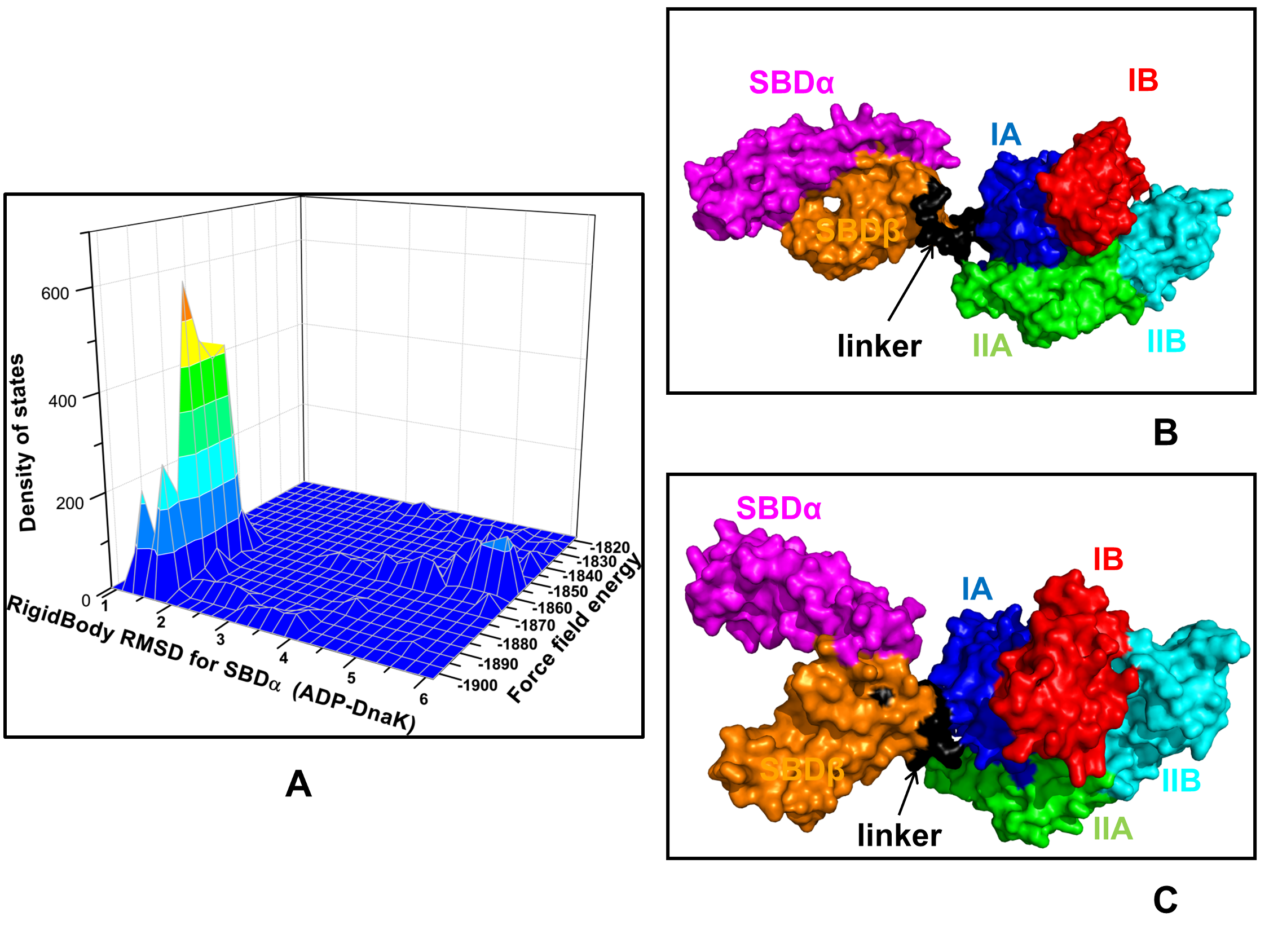

Supplement: S3 Fig — The density of states for the ADP-DnaK was derived from the equilibrium conformational ensemble. (A) The density distribution as a function of the force field energy and the rigid body RMSD for the SBD-α subdomain from its native position in the ADP-DnaK structure (pdb id 2KHO). (B) The domain-undocked crystallographic conformation corresponds to the dominant peak in the density distribution. (C) A representative partly undocked conformation, in which the SBD-α lid deviates from the SBD-β–SBD-α interface, corresponds to a shallow intermediate peak in the density distribution. The structures are shown in a surface representation and main structural elements are annotated. The NBD subdomains are colored as follows: IA (in blue), IB (in red), IIA (in green), IIB (in cyan), the inter-domain linker (in black), SBD-α (in magenta), and SBD-β (in orange). (TIF) [file pone.0143752.s003.tif]

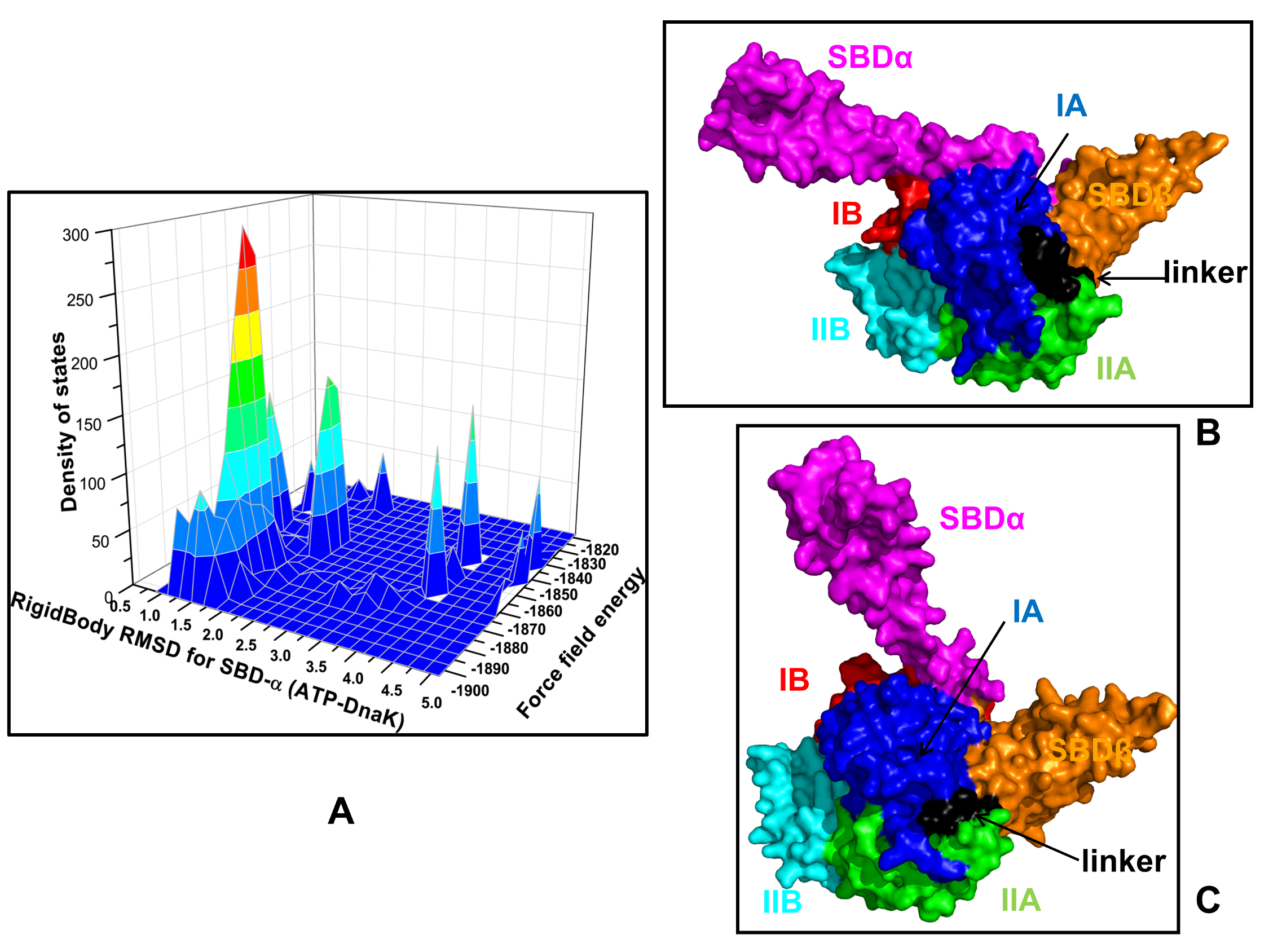

Supplement: S4 Fig — The density of states for the ATP-DnaK ensemble. (A) The density distribution as a function of the force field energy and the rigid body RMSD for the SBD-α subdomain from its native position in the ATP-DnaK structure (B) The domain-docked crystallographic conformation corresponds to the dominant peak in the density distribution. (C) A representative undocked conformation corresponds to the secondary peaks in the distribution. The structures are shown in a surface representation and main structural elements are annotated. The NBD subdomains are colored as in S3 Fig. (TIF) [file pone.0143752.s004.tif]

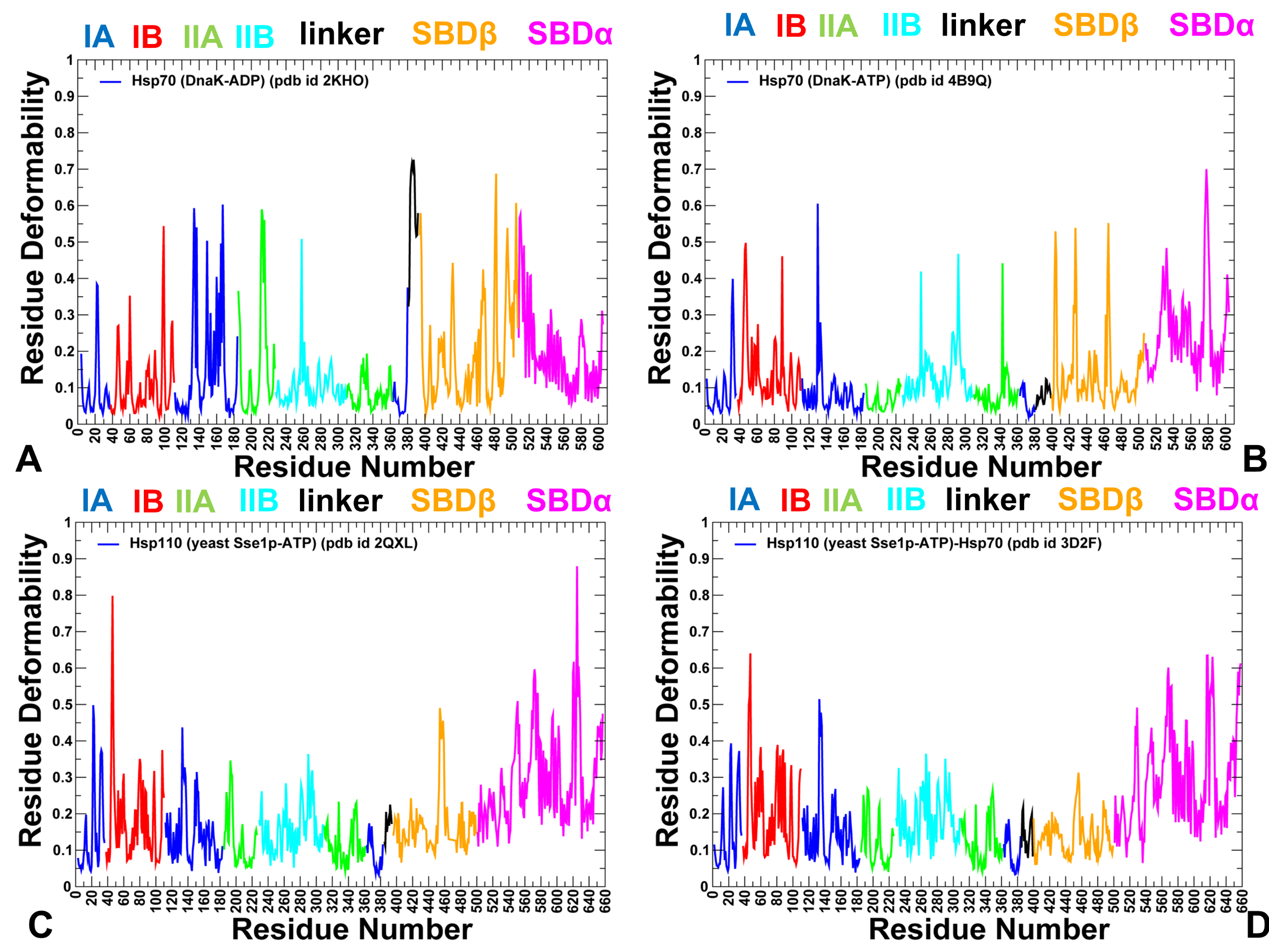

Supplement: S5 Fig — The residue-based deformability profiles of the solution structure of an ADP-bound DnaK, pdb id 2KHO (A); the crystal structure of an ATP-bound DnaK, pdb id 4B9Q (B); the crystal structure of a Sse1p-ATP (C); and the crystal structure of Sse1p in a complex with the NBD of hHsp70 (D). In (D) deformability profile is shown only for Sse1p residues. The deformability profiles are annotated and colored according to the adopted coloring scheme of the chaperone subdomains: IA (in blue), IB (in red), IIA (in green), IIB (in cyan), the inter-domain linker (in black), SBD-α (in magenta), and SBD-β (in orange). (TIF) [file pone.0143752.s005.tif]

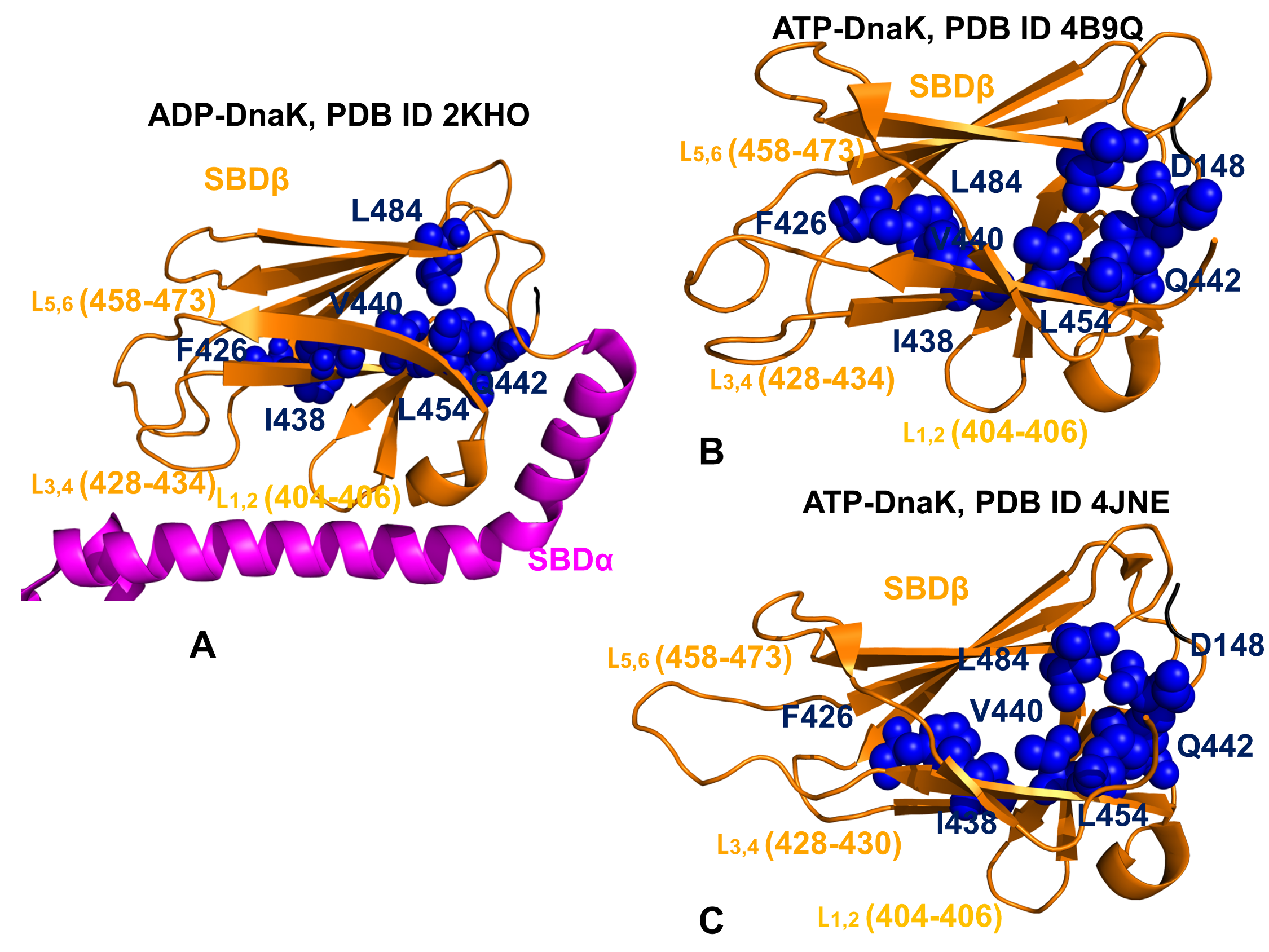

Supplement: S6 Fig — The hydrophobic core residues (V440, L454, L484) are mapped along with the F426 and I438 (substrate binding site hinge points), Q442 and D148(NBD) residues in the ADP-DnaK (A, pdb id 2KHO) and the ATP-DnaK structures (B, pdb id 4B9Q), (C, pdb id 4JNE). The SBD-β domain is shown in orange ribbons and the substrate binding loops are annotated: L1,2 (residues 404–406), L3,4 (residues 428–434), and L5,6 (residues 458–473). In the ADP-DnaK form (A) the SBD-α lid is also shown (in magenta ribbons). Residues are shown in blue spheres and annotated. Note the formation of the interacting clusters in the ATP-DnaK structures that link the substrate binding site (F426 and I438) with the hydrophobic core (V440, L454, L484) and the inter-domain interface (Q442, D148). (TIF) [file pone.0143752.s006.tif]

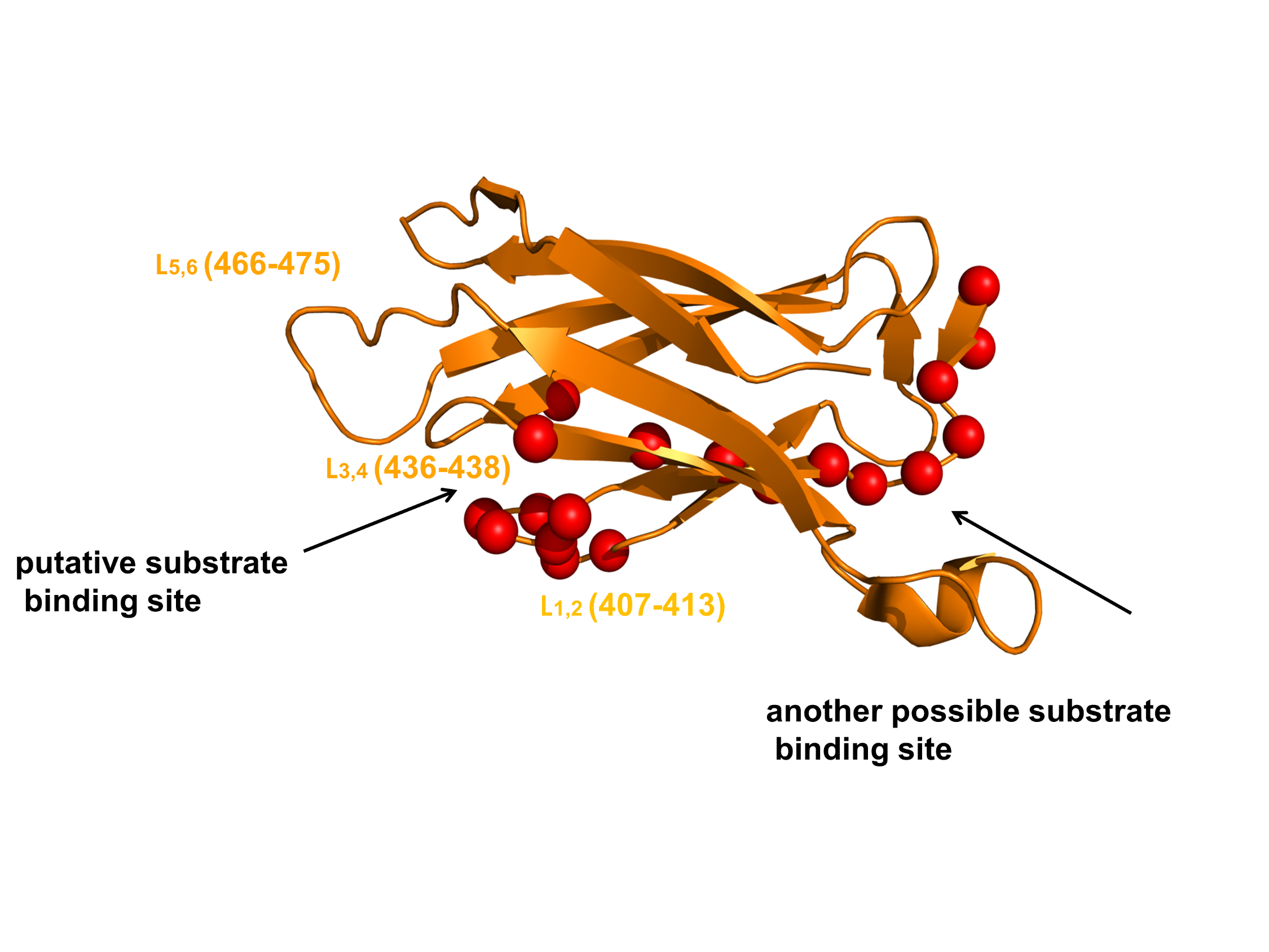

Supplement: S7 Fig — The SBD-β residues with the small RD values (and low protection level) are shown in red spheres. The SBD-β domain is shown in orange ribbons and the substrate binding loops are annotated: L1,2 (residues 407–413), L3,4 (residues 436–438), and L5,6 (residues 466–475). The locations of the putative substrate recognition site are indicated by arrow. (TIF) [file pone.0143752.s007.tif]

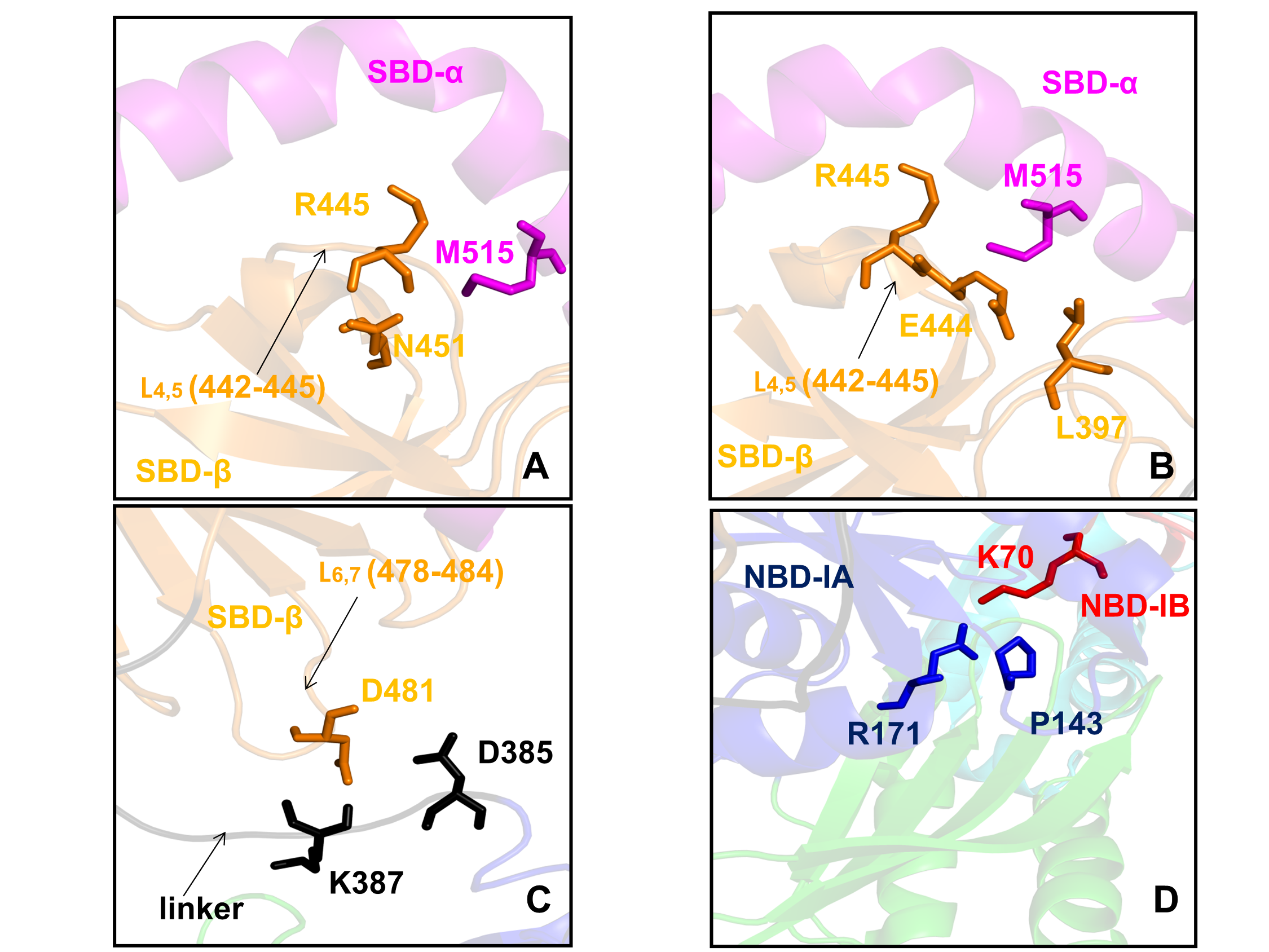

Supplement: S8 Fig — Structural mapping of principal local communities in the ADP-bound DnaK. A close-up of interactions in local communities formed between SBD-β and SBD-α residue (R445-N451-M515) (A) and (L397-E444-M515) (B). A community (D481-K387-L385) of residues from the SBD-β and the linker regions (C). A community (K70-E171-P143) connects regulatory residues in NBD. The interacting residues are shown in colored sticks, the DnaK domains are shown in ribbons with a reduced transparency. Annotation and coloring are according to the adopted scheme: IA (in blue), IB (in red), IIA (in green), IIB (in cyan), the inter-domain linker (in black), SBD-α (in magenta), and SBD-β (in orange). The residue numbering is in accordance with the solution structure of an ADP-bound DnaK, pdb id 2KHO. (TIF) [file pone.0143752.s008.tif]

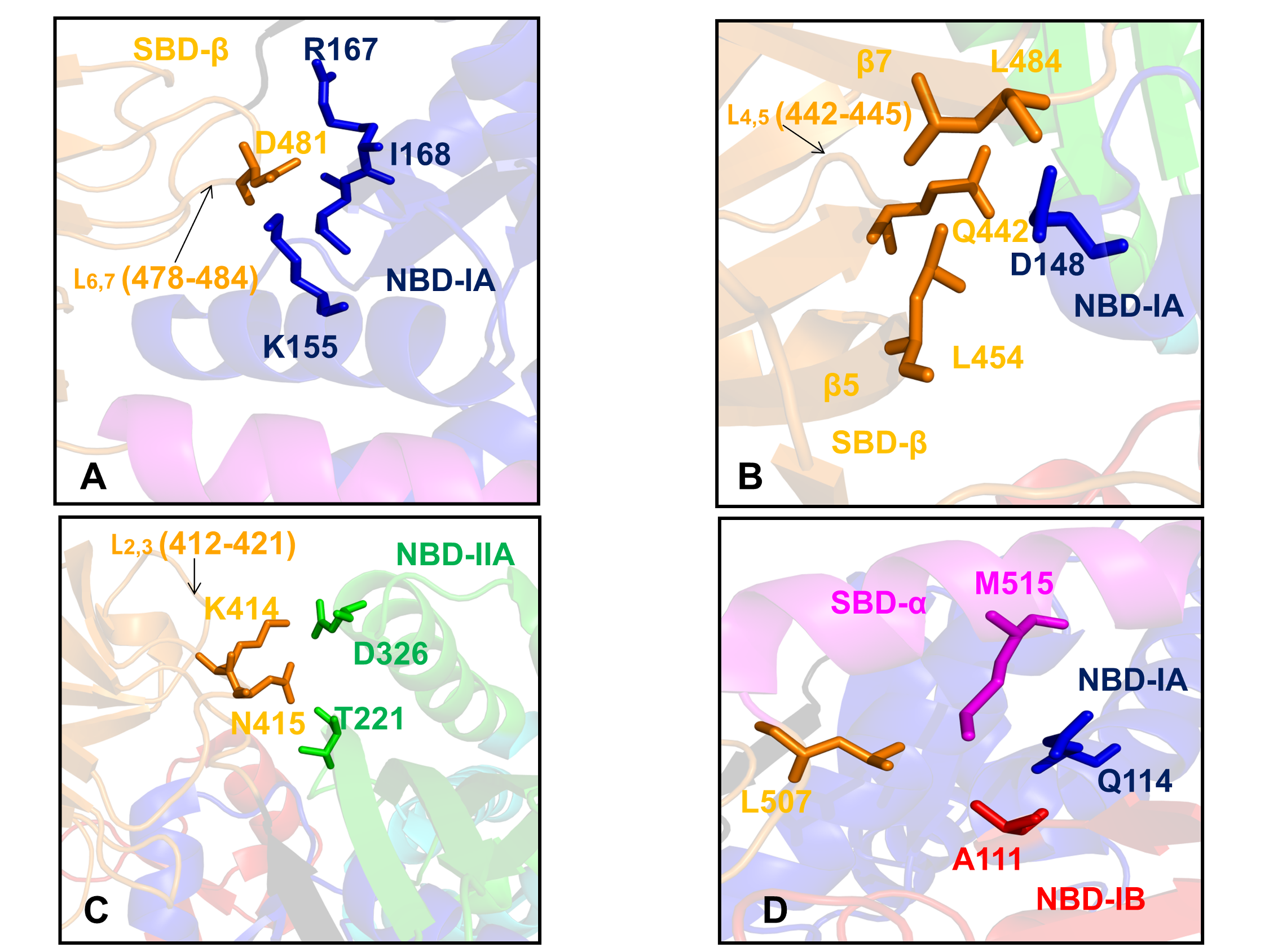

Supplement: S9 Fig — Structural mapping of principal local communities in the ATP-bound DnaK. A close-up view of interactions in a local community (R167-I168-K155-D481) that couples the subdomain IA with the SBD-β (A). An overview of interactions in a local community (Q442-D148-L454-L484) that links L4,5 loop in the SBD-β, hydrophobic core of the SBD-β and subdomain IA of NBD (B). A community (K414-N415-D326-T221) couples residues from L2,3 loop with the subdomain IIA (C). A close-up of interactions in a community (A111-Q114-L507-M515) formed at the NBD-SBD-α interface (D). The interacting residues are shown in colored sticks, the DnaK domains are shown in ribbons with a reduced transparency. Annotation and coloring are according to the adopted scheme. The residue numbering is in accordance with the crystal structure of an ATP-bound DnaK, pdb id 4B9Q. (TIF) [file pone.0143752.s009.tif]

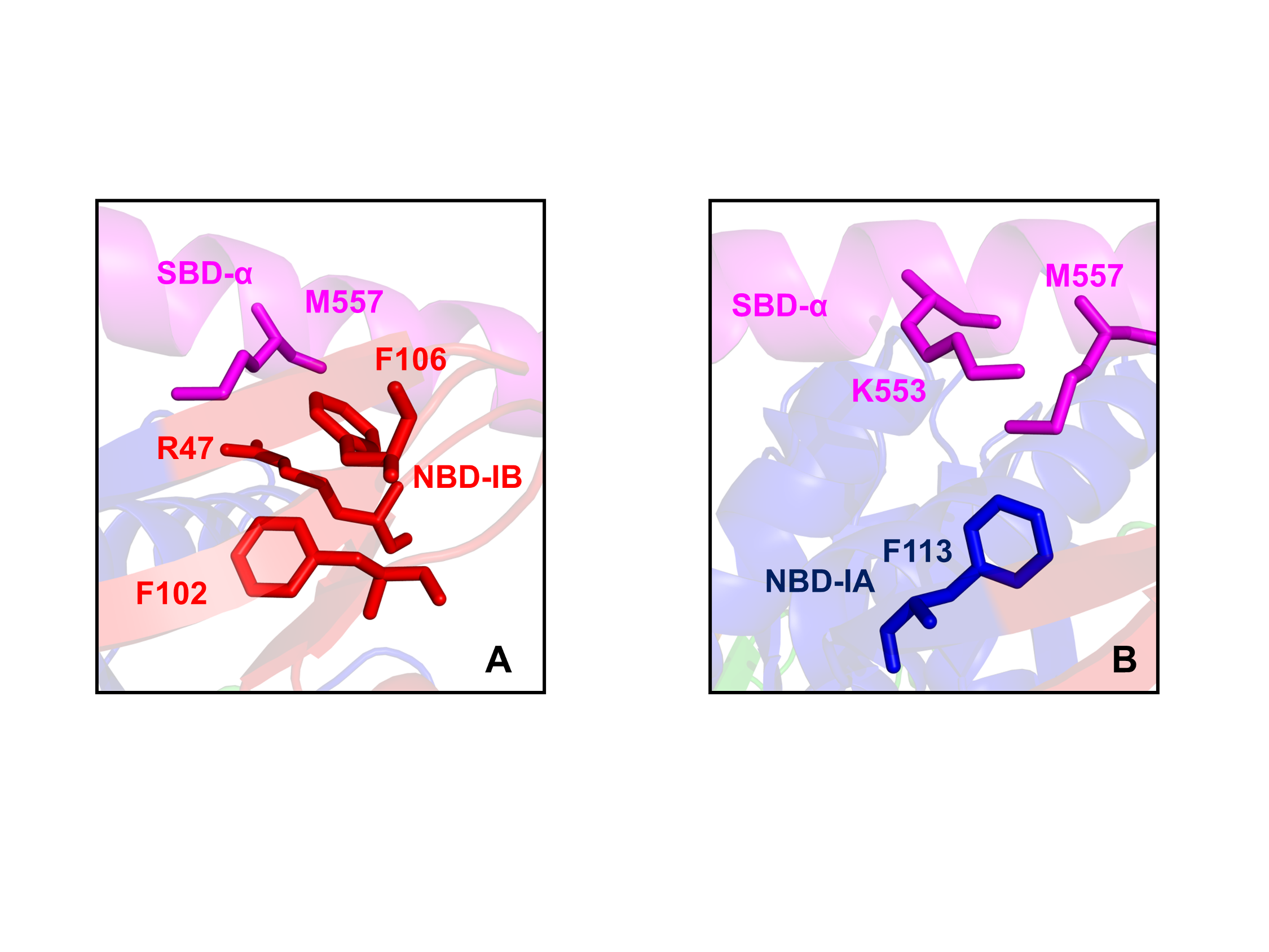

Supplement: S10 Fig — Structural mapping of l local communities in the Sse1p-ATP. A close-up view of interactions in the inter-domain communities involving NBD and SBD-α residues (F42-F106-R47-M557) (A) and (F113-K553-M557) (B). The interacting residues are shown in colored sticks. Sse1p domains are shown in ribbons with a reduced transparency. Annotation and coloring are according to the adopted scheme The residue numbering is in accordance with the crystal structure of an Sse1p-ATP, pdb id 2QXL. (TIF) [file pone.0143752.s010.tif]

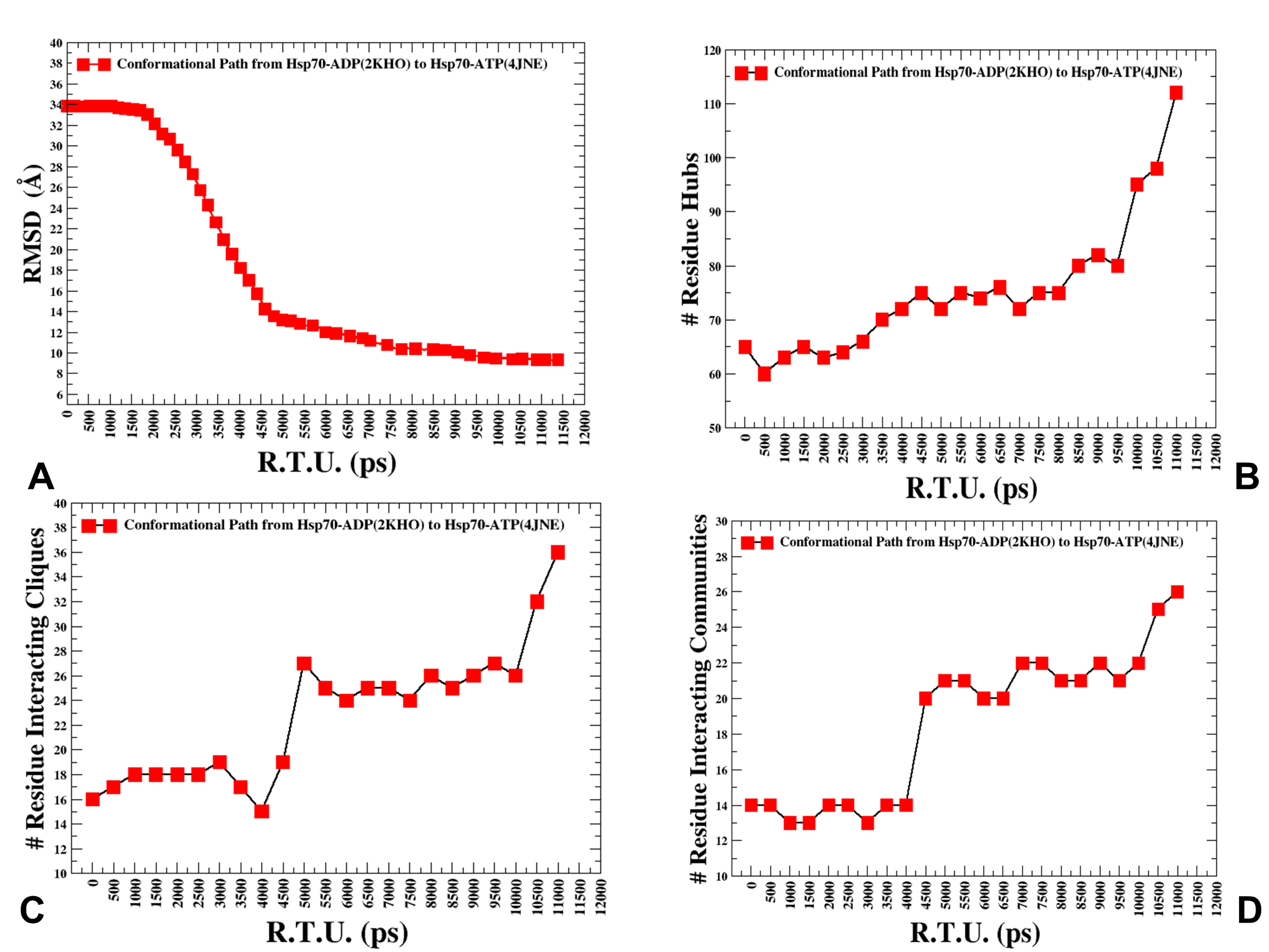

Supplement: S11 Fig — Structural and network parameters of conformational changes between the initial (closed ADP-bound DnaK conformation) and targeted structure (open ATP-bound DnaK conformation). (A) The RMSD between the initial and targeted structures as a function of reduced simulation time units (R.T.U). The RTU parameter is defined according to [152,153] as a ratio of the number of total collisions (scaled by 0.15) to the number of residues in the system at T = 300K. In this model, RTU typically corresponds to 20–50 ps time in conventional equilibrium dynamics. (B-D) Evolution of the network parameters during conformational transitions: number of hubs (B), cliques (C), and communities (D). (TIF) [file pone.0143752.s011.tif]

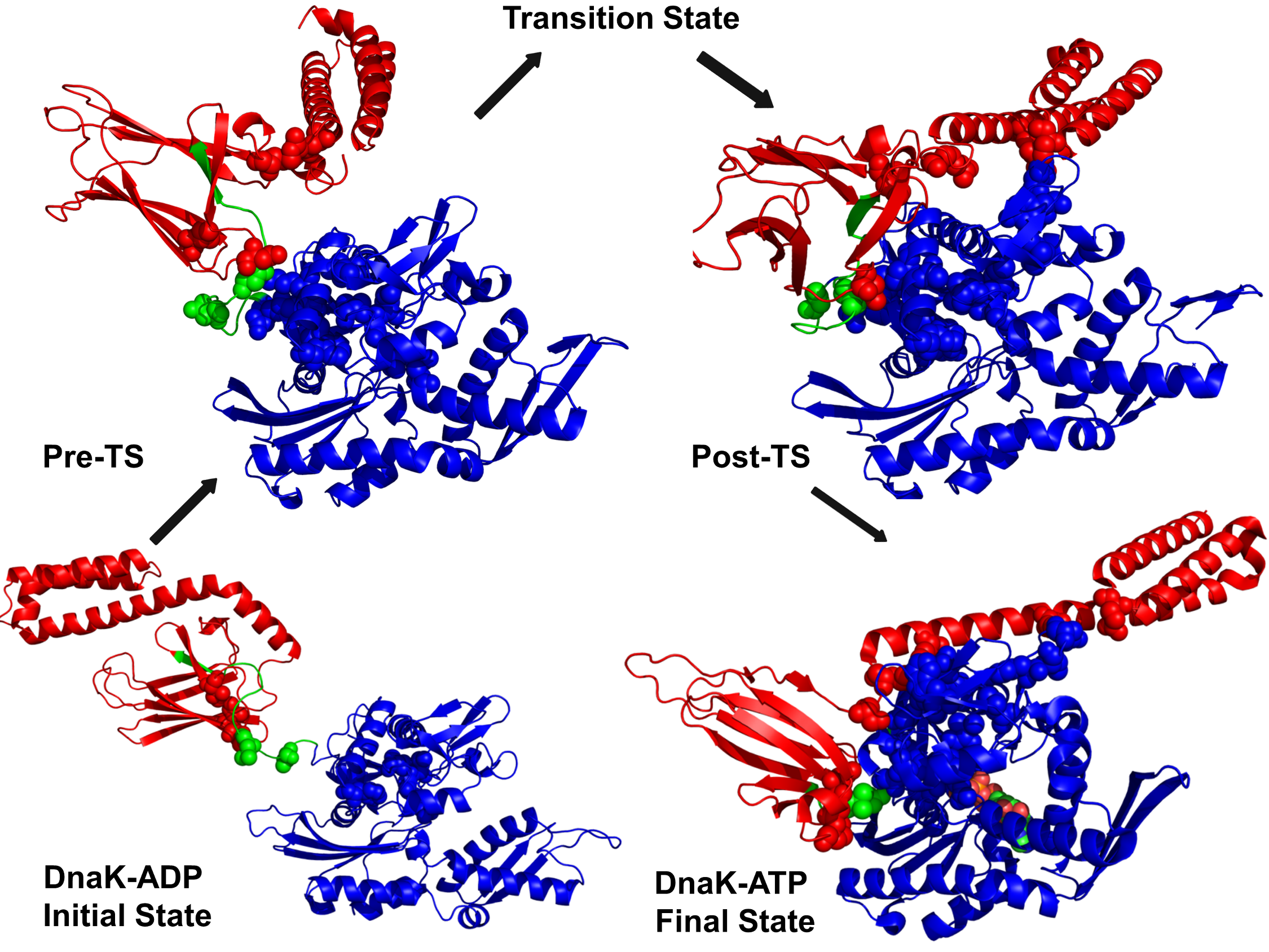

Supplement: S12 Fig — A schematic overview of the conformational transition between the initial (closed ADP-bound DnaK conformation). The initial structure (the solution structure of an ADP-bound DnaK, pdb id 2KHO) and the targeted structure (the crystal structure of an ATP-bound DnaK, pdb id 4B9Q) are shown in ribbons and subdomains are colored as follows: NBD (in blue), SBD (in red), and the inter-domain linker (in yellow). The high centrality inter-domain residues are shown in spheres. The depicted pre-transition state and post-transitional state are representative structures from ensembles of conformations sampled immediately prior and after a major transition. The transition region is approximately defined from the first-order sigmoidal curve. The residues that form the inter-domain contacts in these states are shown in spheres and colored according to their subdomains. (TIF) [file pone.0143752.s012.tif]
